# Light-tunable charge dynamics enable ultralow-power bidirectional lead-free perovskite synapses for intelligent vision

**DOI:** 10.1126/sciadv.aeh3751

**Published:** 2026-09-02

**Authors:** Tingting Mei, Bowei Zhang, Tanzeela Yousaf, Jan Seidel, Dawei Su, Zijian Feng, Tianxu Huang, Fandi Chen, Zhi Li, Chun Hui Wang, Wenlong Cheng, Yuerui Lu, Yichen Liu, Tom Wu, Long Hu, Miaoqiang Lyu, Chun-Ho Lin, Lianzhou Wang, Dewei Chu

**Affiliations:** ^1^ School of Materials Science and Engineering, University of New South Wales, Sydney, NSW, 2052, Australia.; ^2^ Australian Institute for Bioengineering and Nanotechnology, The University of Queensland, St Lucia, Queensland 4072, Australia.; ^3^ School of Chemical Engineering, The University of Queensland, St Lucia, QLD 4072, Australia.; ^4^ Applied Chemistry & Environmental Science, School of Science, RMIT University, Melbourne, VIC 3000, Australia.; ^5^ School of Mechanical and Manufacturing Engineering, University of New South Wales, Sydney, NSW 2052, Australia.; ^6^ School of Biomedical Engineering, University of Sydney, Darlington 2008, NSW, Australia.; ^7^ School of Engineering, Australian National University, Canberra, ACT, 2601, Australia.; ^8^ School of Materials Science and Engineering, Southeast University, Nanjing, 211189, China.; ^9^ Department of Applied Physics, The Hong Kong Polytechnic University, Hong Kong, 999077, China.; ^10^ Department of Applied Biology and Chemical Technology, The Hong Kong Polytechnic University, Kowloon, Hong Kong, 999077, China.

## Abstract

Advanced vision systems for autonomous robotics require high efficiency, ultralow power consumption, and real-time decision-making. However, conventional vision sensors that rely on single-mode optical excitation fundamentally constrain their versatility. Here, we report a fully light-tunable optoelectronic synaptic device based on perovskite PEA_2_SnI_4_/C_60_ heterostructures that uniquely supports dual-mode bidirectional synaptic behaviour with potentiation under visible light and depression under near-infrared illumination, enabled by a synergistic mechanism of sub-bandgap absorption and interfacial carrier trapping-detrapping for wavelength-selective control. The heterostructure device features long-lasting synaptic plasticity, with excitatory and inhibitory retention times of 350 s and over 8000 s, respectively, and an ultralow energy consumption of ∼1 fJ per event. These exceptional characteristics enable unprecedented performance in neuromorphic vision tasks, including attention-enhanced traffic sign recognition, physical reservoir computing, and dynamic target detection using a 7 × 7 array device, which establishes a powerful and energy-efficient platform for next-generation robotic eyes and advances all-optical neuromorphic perceptions toward intelligent autonomy.

## INTRODUCTION

Humanoid robots are evolving rapidly from experimental prototypes into practical systems, with the potential to transform healthcare, mobility, industry, and everyday life. As these technologies mature, they are expected to become indispensable partners that enhance human productivity, wellbeing and social interaction (*1*–*3*). Despite remarkable advances to date, a critical challenge remains: current computing architectures impose a high energy burden because sensing, data-processing, and storage are separated, leading to substantial data transfer and power dissipation (*4*–*6*).

In contrast, biological neural systems can achieve energy-efficient and adaptive learning by dynamically balancing excitatory and inhibitory synaptic plasticity within a tightly integrated perception-computation-memory framework (*7*–*10*). Inspired by these principles, optoelectronic synapses have emerged as a promising platform for neuromorphic hardware for vision-driven perception (*9*, *11*). Advances in materials engineering and device architectures have recently led to encouraging breakthroughs (*12*–*15*). However, most reported optoelectronic synapses have predominantly focused on emulating excitatory plasticity, while giving less attention to equally important inhibitory plasticity (*16*–*18*) (table S3).

In vision cognition, inhibitory synaptic plasticity is indispensable for suppressing redundant inputs, enhancing selectivity, and enabling attentional control, thereby facilitating more efficient information processing in complex interactive environments (*19*, *20*). However, most devices rely on photogenerated carriers to enhance channel conductance through charge accumulation or photogating under light stimulation (*21*, *22*). This intrinsic bias toward excitation renders optical depression far more challenging, as it requires the controllable reduction rather than buildup of carriers. Consequently, most of the reported systems resort to electrical erasure to discharge accumulated photocarriers (*23*–*25*), rather than optically driven inhibitory plasticity.

Recent studies suggest that coupling optical stimuli with electrical gate control can induce depression, where ferroelectric polarization reversal (*26*) or bias-induced field redistribution in heterojunctions (*27*) enables bidirectional synaptic responses across different spectral bands within a single device. However, compared with electro-optical hybrid schemes, fully light-driven operation is far more appealing for achieving ultralow power consumption and seamless photonic integration. Existing all-optical approaches face key limitations: defect ionization (*13*) or photothermal heating (*26*) are inherently energy-intensive, while defect-mediated recombination in hybrid material systems suffers from poor reproducibility and trap saturation (*28*–*30*) (table S4). These limitations highlight the need for a new working mechanism that can deliver robust, energy-efficient inhibitory control without relying on stochastic defects or thermal processes.

**Fig. 1.**
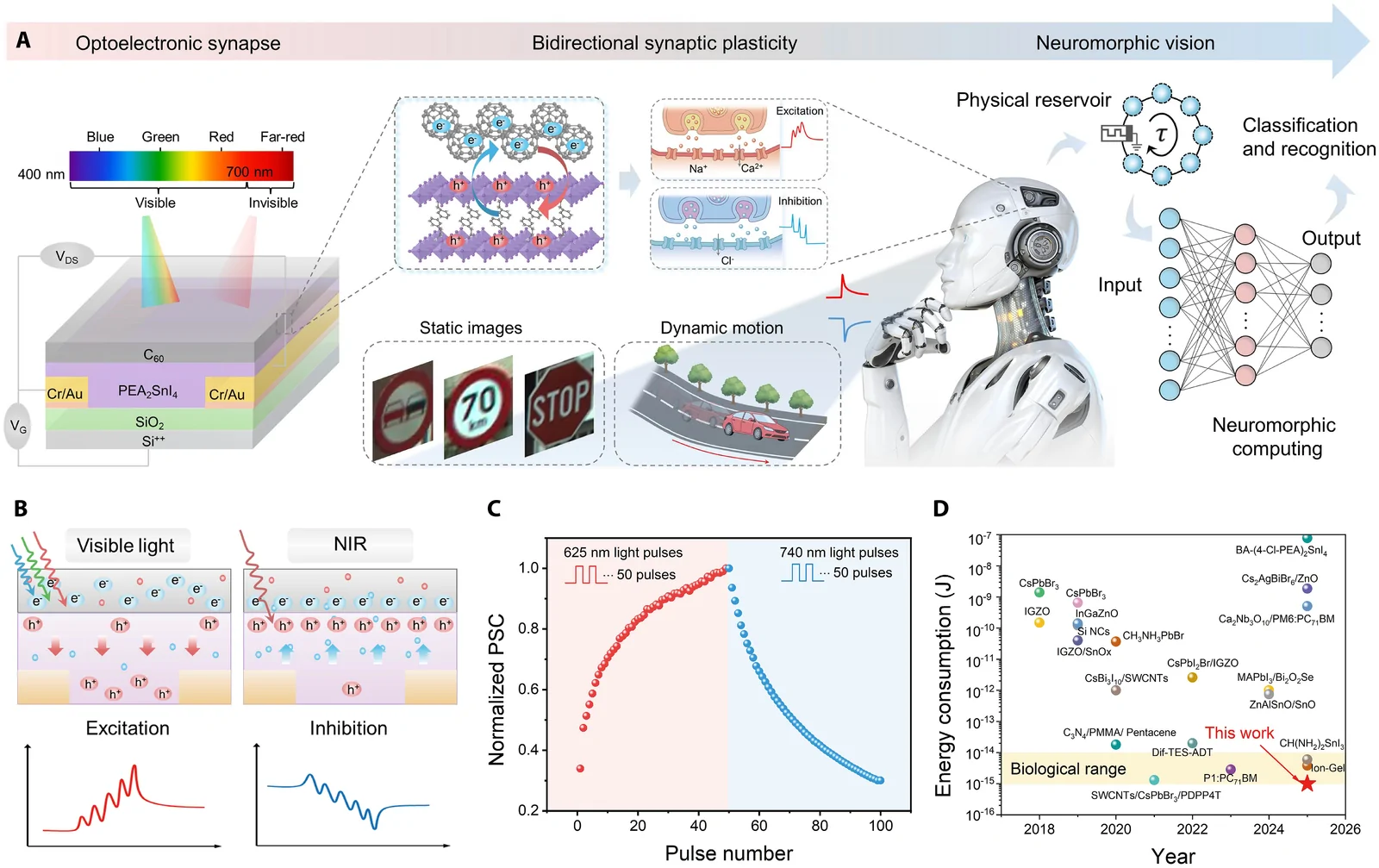
All-optical bidirectional synaptic plasticity in a PEA_2_SnI_4_/C_60_ heterojunction and neuromorphic vision demonstrations. (**A**) Schematic illustration of a heterojunction-based optoelectronic synapse with bidirectional synaptic plasticity and its applications in neuromorphic vision. Robot image: iStock.com / iLexx. (**B**) Schematic of working mechanism of excitation and inhibition synaptic behaviour. (**C**) Excitation synaptic behaviour under 625 nm light pulses and inhibitory synaptic behaviour under 740 nm light pulses. (**D**) Energy consumption comparison with recently reported optoelectronic synapses (*18*, *22*, *58*–*76*).

Herein, we report a new working principle of all-optical modulation of interfacial carrier transfer dynamics to achieve controllable excitatory and inhibitory responses within a single device. By the deliberate use of sub-bandgap excitation in conjunction with engineered interfacial charge dynamics in a lead-free halide perovskite/C_60_ heterostructure, we realise an infrared-driven inhibitory response enabled by interfacial photocarrier transfer, which can be combined with its visible-light excitatory function, achieving spectrally decoupled excitation and inhibition with ultralow energy consumption of ∼1 fJ (Fig. 1, A and D). Unlike above-bandgap excitation, infrared photons deliver lower energy per event, minimize thermal loss, and selectively trigger interfacial charge transfer pathways. By leveraging wavelength-dependent carrier transport at the heterojunction interface, the device enables bidirectional synaptic modulation, exhibiting excitatory learning under visible light while producing inhibitory relaxation under near-infrared illumination (Fig. 1, B and C). Benefiting from spectral-dependent synaptic modulation, we further validate the multifunctional neuromorphic performance of the heterostructure in three visual cognition tasks: (i) attention-enhanced traffic sign recognition, (ii) reservoir computing for digit classification, and (iii) spatiotemporal pattern recognition using a 7 × 7 dynamic array. This work establishes, to the best of our knowledge, the first all-optical synaptic mechanism that intentionally exploits sub-bandgap excitation for wavelength-selective control and demonstrates device-level emulation of complex visual functions with high fidelity and ultralow energy consumption, thus paving the way for next-generation neuromorphic vision systems for robotic perception applications.

## RESULTS

### Design of 2D perovskite photonic synapse

**Fig. 2.**
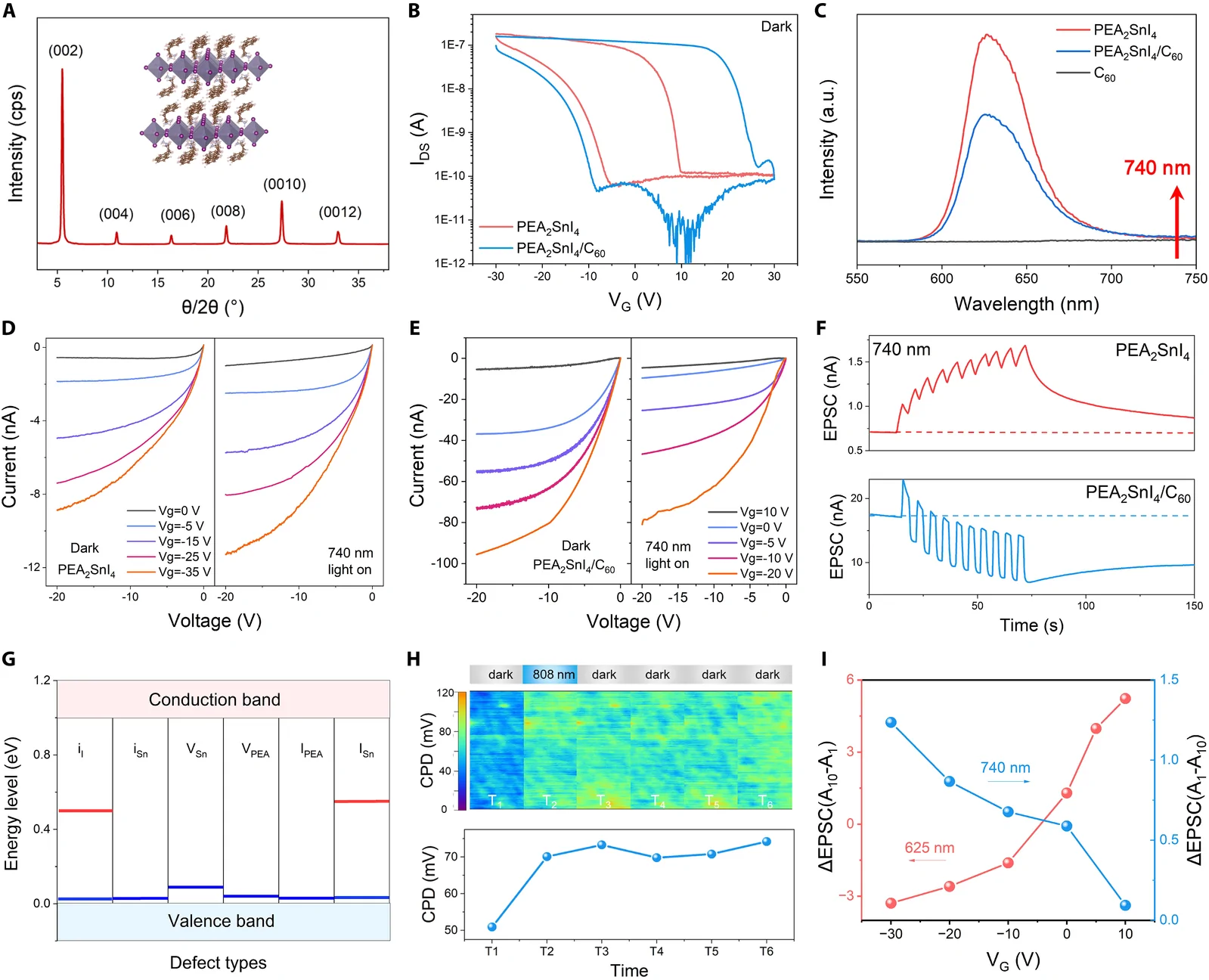
Characteristics of PEA_2_SnI_4_ and PEA_2_SnI_4_/C_60_ devices and mechanism analysis. (**A**) XRD of PEA_2_SnI_4_ (inset: the crystal structure of PEA_2_SnI_4_). (**B**) The transfer curve of PEA_2_SnI_4_ device and PEA_2_SnI_4_/C_60_ device under V_DS_ = 5 V. (**C**) PL spectra of PEA_2_SnI_4_, PEA_2_SnI_4_/C_60_, and C_60_ thin films. (**D**) Output curve of PEA_2_SnI_4_ device under dark environment and 740 nm light illumination. (**E**) Output curve of PEA_2_SnI_4_/C_60_ devices under dark environment and 740 nm light illumination. (**F**) Post-synaptic current of both PEA_2_SnI_4_ and PEA_2_SnI_4_/C_60_ devices under 740 nm light pulses with 3 s on and 3 s off. (**G**) Defect energy levels by DFT calculation. (**H**) KPFM measurement of PEA_2_SnI_4_/C_60_ device under 808 nm. (**I**) Summary of EPSC (under 625 nm) and IPSC (under 740 nm) change with different V_G_.

The optoelectronic synaptic device is constructed based on a bottom-gate bottom-contact transistor structure using a lead-free two-dimensional (2D) tin-based halide perovskite, phenethylammonium tin iodide (PEA_2_SnI_4_), as a photoactive layer. The PEA_2_SnI_4_ thin films were prepared with a solution-based spin-coating process using toluene as the anti-solvent and L-glutathione (GSH) as the additive (see Experimental Section for details) (*31*). The x-ray diffraction (XRD) pattern (Fig. 2A) exhibits a series of characteristic (00 l) diffraction peaks (l = 2, 4, 6, 8, and 12), confirming the formation of a layered 2D perovskite structure and a high degree of crystallinity (*32*). The elemental ratio of Sn^4+^/Sn^2+^ determined from the integrated peak areas of x-ray photoelectron spectroscopy (XPS) (fig. S2) is only 0.36, lower than the references (*31*, *33*), suggesting reduced Sn(II) oxidation based on our synthetic protocol. Tauc plot from UV-vis absorption spectroscopy (fig. S3) revealed an optical bandgap of approximately 1.96 eV. Combining this bandgap with the ultraviolet photoelectron spectroscopy (UPS) measurements (fig. S4), the valence band maximum (VBM) and conduction band minimum (CBM) of the perovskite were determined to be −5.24 eV and −3.28 eV, respectively. To facilitate synaptic functionality, a thin C_60_ layer (10 nm), a widely used electron-accepting material (*34*, *35*), was deposited atop the PEA_2_SnI_4_ film (45 nm) to capture and retain photo-induced carriers, thereby prolonging conductance states and mimicking synaptic memory behaviours (figs. S5 and S6).

Two types of thin-film transistor architectures, pristine PEA_2_SnI_4_ and PEA_2_SnI_4_/C_60_, were subsequently fabricated following the procedures detailed in the Methods section, and their electrical performance was systematically evaluated. Both devices exhibited an on/off ratio exceeding 10^3^ and pronounced hysteresis in their transfer characteristics, indicating the history-dependent conductance memory effect (Fig. 2B). The extracted field-effect mobilities were around 1.5 cm^2^ V^−1^ s^−1^ for both devices. Notably, the PEA_2_SnI_4_/C_60_ device displayed a markedly enlarged hysteresis window, suggesting enhanced charge trapping because of the heterostructure design. This distinction is further corroborated by the retention tests under continuous bias stress (fig. S7), where the PEA_2_SnI_4_ device exhibits a rapid decrease in on/off ratio to ∼10 after 1000 s, while the PEA_2_SnI_4_/C_60_ device maintains a high conductivity contrast exceeding 10^3^, confirming its superior charge retention capability.

To further investigate the optoelectronic characteristics of the perovskite films, we performed photoluminescence (PL) spectroscopy, in which the PEA_2_SnI_4_ film exhibits a pronounced emission peak centred at approximately 623 nm, consistent with its direct bandgap of 1.96 eV (Fig. 2C). In the PEA_2_SnI_4_/C_60_ heterostructure, the PL emission (tested at the perovskite side) is obviously quenched. As C_60_ shows no intrinsic photoluminescence, this quenching signifies the efficient transfer of photoexcited carriers from perovskite to C_60_. Owing to the strong photoresponse across the visible spectrum, we found that both perovskite transistors could not be completely turned off under illumination at 470 nm, 554 nm, or 625 nm within the studied gate voltage window (−30 to +30 V) (fig. S8), and exhibited desirable excitatory postsynaptic current (EPSC) behavior upon light stimulation (figs. S9 and S10) that is beneficial for excitatory synaptic applications. Notably, the PEA_2_SnI_4_/C_60_ device demonstrates markedly prolonged EPSC relaxation after the light is switched off, suggesting enhanced charge trapping and longer-term plasticity enabled by the C_60_ layer. While most studies have focused on above-bandgap excitation (*36*, *37*), we further investigated the device response under sub-bandgap illumination at 740 nm to assess its potential for wavelength-dependent modulation. It is worth noting that sub-bandgap absorption in 2D lead-based perovskites has been reported previously, whereas such behaviour remains rarely explored in tin-based perovskite systems (*38*–*40*).

For the two types of PEA_2_SnI_4_ device configurations investigated here, interestingly, the transfer characteristics show divergent hysteresis trends under 740 nm excitation, which are typically associated with charge-trapping phenomena (fig. S11) (*41*). The pristine PEA_2_SnI_4_ device exhibits a broadened hysteresis window with increasing light intensity, indicative of an additional photogating effect induced by long-lived trapped charges generated from sub-bandgap photon excitation, which shifts the threshold voltage of the backward sweep (*39*). By contrast, the PEA_2_SnI_4_/C_60_ device shows an intrinsically wide hysteresis window originating from interfacial charge transfer. Under increasing near-infrared (NIR) illumination, the forward sweep curve shifts toward more positive gate voltages, indicating enhanced electron transfer of photoexcited electrons to C_60_ with simultaneous hole accumulation in the channel, thereby requiring a more positive gate bias to fully switch off the device and confirming the electron-trapping mechanism in the heterostructure (*42*, *43*).

This heterostructure design gives rise to a distinct photo-inhibitory behaviour, as revealed by the output characteristics (Fig. 2, D and E), where the source-drain current of the pristine PEA_2_SnI_4_ device increases under 740 nm illumination, whereas that of the PEA_2_SnI_4_/C_60_ device decreases. The photoresponse dynamics are more clearly resolved in the repeated light-pulse measurements (Fig. 2F), in which the PEA_2_SnI_4_/C_60_ device shows a sharp initial rise followed by a decay to below its initial level. These results indicate that the incorporation of the C_60_ interlayer fundamentally alters the sub-bandgap photoresponse, converting the phototransistor mechanism from excitatory to inhibitory mode. Remarkably, the inhibitory synaptic response of PEA_2_SnI_4_/C_60_ device persists even under 850 nm light pulses (fig. S12), well beyond the material’s intrinsic absorption range. The long-wavelength inhibition reflects the distinct photo-modulatory behaviors and interfacial charge dynamics of the heterostructure. To elucidate the underlying mechanisms governing these distinct photoresponses, two key questions must be addressed: (i) why the devices exhibit photoactivity at wavelengths beyond their optical absorption range, and (ii) how the introduction of the C_60_ layer enables inhibitory synaptic behavior.

### Mechanism of 2D perovskite photonic synapses

To systematically address the two central questions raised above, we conducted a series of targeted experiments to unravel the underlying physical mechanisms. Although PEA_2_SnI_4_ exhibits a well-defined absorption onset in the visible range, UV-vis spectroscopy (fig. S13) revealed a residual absorption tail extending into the near-infrared region, indicative of a non-negligible sub-bandgap absorption. The pronounced absorption tail suggests the presence of optically active in-gap states that broaden the spectral response beyond the band edge. Furthermore, the PL spectrum also exhibits a broad emission feature in the 800–900 nm range (fig. S14), indicative of radiative recombination via sub-bandgap trap states. These results suggest that defect states within the bandgap can serve as intermediate levels for sub-bandgap photon absorption and carrier generation (*44*).

Further insight into the origin of these sub-bandgap states was obtained through density functional theory (DFT) calculations, which considered a comprehensive set of intrinsic point defects, including interstitials (i_I_, i_Sn_), vacancies (V_PEA_, V_Sn_, V_I_), cation substitutions (Sn_PEA_), and anti-site configurations (I_PEA_, I_Sn_, Sn_I_) (Text S1, fig. S15). The calculated defect-resolved density of states (DOS) reveals that intrinsic defects perturb the electronic structure in a configuration-dependent manner (Fig. 2G). Interstitial defects introduce additional localized states, with i_Sn_ mainly coupling to the valence-band edge to form shallow levels, whereas i_I_ generates both shallow and deep defect states (fig. S16). Vacancy defects also show species-dependent effects, where V_I_ and V_Sn_ modify the Sn-I framework and introduce gap states, whereas V_PEA_ has a weaker band-edge influence owing to the negligible contribution of PEA to the pristine VBM and CBM (fig. S17). Substitutional and anti-site defects further reconstruct the local coordination environment, with Sn_PEA_, I_PEA_ and I_Sn_ generating localized in-gap states (fig. S18). Notably, the deep states associated with i_I_ and I_Sn_ are particularly relevant, as they can serve as sub-bandgap excitation centers and non-radiative trapping sites that mediate the observed NIR-assisted optoelectronic response. Previous studies have found that halide interstitial and B-site cation vacancy are the most possible and stable defects due to their low defect formation energy (DFE) values, while the halide-on-metal substitutions generally possess relatively high DFE and thus exist at low equilibrium concentrations under typical synthesis conditions (*45*–*47*). In our system, the formation of i_I_ may generate occupied in-gap states that act as deep electron traps, giving rise to pronounced sub-bandgap absorption features (*48*, *49*). The Urbach energy of the pristine PEA_2_SnI_4_ film (73.9 meV, fig. S19) further corroborates the presence of in-gap trap states that contribute to the observed sub-bandgap absorption. Consequently, under sub-bandgap illumination (740 nm and 850 nm), the pure perovskite device exhibits a pronounced excitatory behavior, consistent with photoexcitation through defect-assisted trap-to-band transitions (*50*, *51*).

In contrast, the PEA_2_SnI_4_/C_60_ heterostructure displays a distinct wavelength-dependent response. When illuminated with above-bandgap photons (625 nm), abundant photocarriers are generated within the perovskite layer (6.35 × 10^15^ cm^−3^ in the present case under 0.08 mW cm^−2^ illumination for 1 s; fig. S20, Text S2), which dominates charge transport and leads to excitatory synaptic potentiation similar to the pristine perovskite. However, under sub-bandgap excitation (740 nm), a markedly different trend emerges, as the heterostructure’s influence becomes more pronounced owing to the reduced photocarrier generation (1.63 × 10^14^ cm^−3^ in case under 2.7 mW cm^−2^ illumination for 1 s) via trap-assisted absorption.

Although a positive photocurrent is initially generated, the EPSC rapidly decreases upon removal of illumination, indicating an inhibitory synaptic response. As the C_60_ layer (∼10 nm) does not show any response under NIR illumination (fig. S21), this bidirectional photoresponse originates from interfacial charge transfer dynamics between the perovskite and C_60_ layer. Under near-infrared illumination, the in-gap trap states in the perovskite layer absorb sub-bandgap photons and generate trap-to-band excited electrons. Although this weak photogeneration gives rise to a transient current increase immediately after light-on, the subsequent current decay reveals a progressive interfacial transfer process with an apparent transfer rate at 6.9 × 10^13^ cm^−3^·s^−1^ (fig. S22). These electrons are efficiently transferred to and captured by the C_60_ overlayer, which functions as an electron-trapping layer. After the removal of light, these trapped electrons remain localized in the C_60_, generating a persistent interfacial electric field that attracts holes away from the p-type perovskite channel. The resultant depletion of hole carriers suppresses the channel current, producing the inhibitory synaptic behavior. To formulate a quantitative criterion for the excitatory-to-inhibitory transition, we define a dimensionless competition factor: $T=\alpha p_{\mathrm{ph}}/R_{n\_\mathrm{ct}}t,$ where *p*_ph_ is the effective photogenerated carrier population, *R*_n_ct_ is the apparent interfacial transfer rate, *t* is the illumination duration time, and α represents the electrostatic coupling efficiency between mobile and trapped charges. Under visible light, photogeneration outweighs interfacial trapping, resulting in *T* > 1 and the device operating in an excitatory regime; under NIR light, the markedly reduced photocarrier generation allows trapping-induced carrier depletion to dominate, leading to *T* < 1 and the device switches to an inhibitory regime. This quantitative criterion provides a predictive framework for understanding the polarity switching in the heterojunction synapse.

Kelvin probe force microscopy (KPFM) measurements directly confirm this interfacial charge-trapping process (Fig. 2H, fig. S23). Under near-infrared illumination (808 nm), the perovskite/C_60_ heterostructure exhibits a substantial increase in surface contact potential difference (CPD) on the C_60_, indicating efficient electron extraction from the perovskite layer and accumulation on the C_60_ surface. Notably, the elevated CPD persists even after the light is turned off, evidencing long-lived electron trapping within the C_60_ film. These trapped electrons thus create an internal electrostatic field across the heterointerface, leading to channel-current inhibition in the p-type perovskite transistor.

To further probe the directionality of charge transfer under different wavelengths, gate-dependent measurements were performed on the PEA_2_SnI_4_/C_60_ transistor during optical stimulation. At 625 nm, positive gate bias enhances EPSC, while negative bias suppresses it, consistent with perovskite-dominated photogeneration and the fact that negative gating promotes electron capture in the C_60_ trapping layer (fig. S24). Under 740 nm illumination, where the transistor exhibits inhibitory synaptic behavior, the negative gate promotes the inhibitory postsynaptic current (IPSC) response, while the positive gate weakens it (Fig. 2I, fig. S25). The temperature-dependent single- (fig. S26) and multi-pulse (fig. S27) measurements reveal a wavelength-specific thermal activation behaviour. While 625 nm excitation remains excitatory but shows suppressed pulse-to-pulse potentiation at elevated temperatures, the 740 nm sub-bandgap response becomes increasingly inhibitory and accumulative with increasing temperature. These trends indicate that visible excitation is dominated by near-band-edge photogeneration, where elevated temperatures promote carrier transfer to C_60_, thereby slightly suppressing the potentiation effect. In contrast, NIR inhibition arises from thermally assisted, deep-level-mediated carrier release and electron transfer to C_60_, which strengthens interfacial hole depletion in the perovskite channel at high temperature.

These results confirm that the bidirectional synaptic behavior arises from wavelength-governed dominance in carrier dynamics: under visible (above-bandgap) excitation, photogeneration in the perovskite channel dominates and yields excitatory potentiation, while moderate electron trapping in the C_60_ layer plays only a minor role; under near-infrared (sub-bandgap) excitation, the carrier population is limited to trap-to-band transitions, allowing C_60_-mediated electron trapping to dominate and produce the inhibitory response (fig. S28).

Beyond elucidating the underlying mechanism, the demonstrated gate-tunable synaptic response offers an additional degree of freedom for dynamically regulating the learning rate and synaptic weight in optoelectronic neuromorphic systems. Combined with the wavelength-dependent excitatory and inhibitory plasticity, electrical gating on PEA_2_SnI_4_/C_60_ synaptic transistor enables fine control over both the amplitude and polarity of synaptic responses. By modulating the balance between carrier generation and trapping, the device can flexibly tune the strength, direction, and persistence of photoinduced plasticity. Such multifunctional controllability is highly desirable for implementing adaptive learning rules and real-time weight updates in future hardware-based neural networks.

### Excitatory and inhibitory synaptic plasticity

**Fig. 3.**
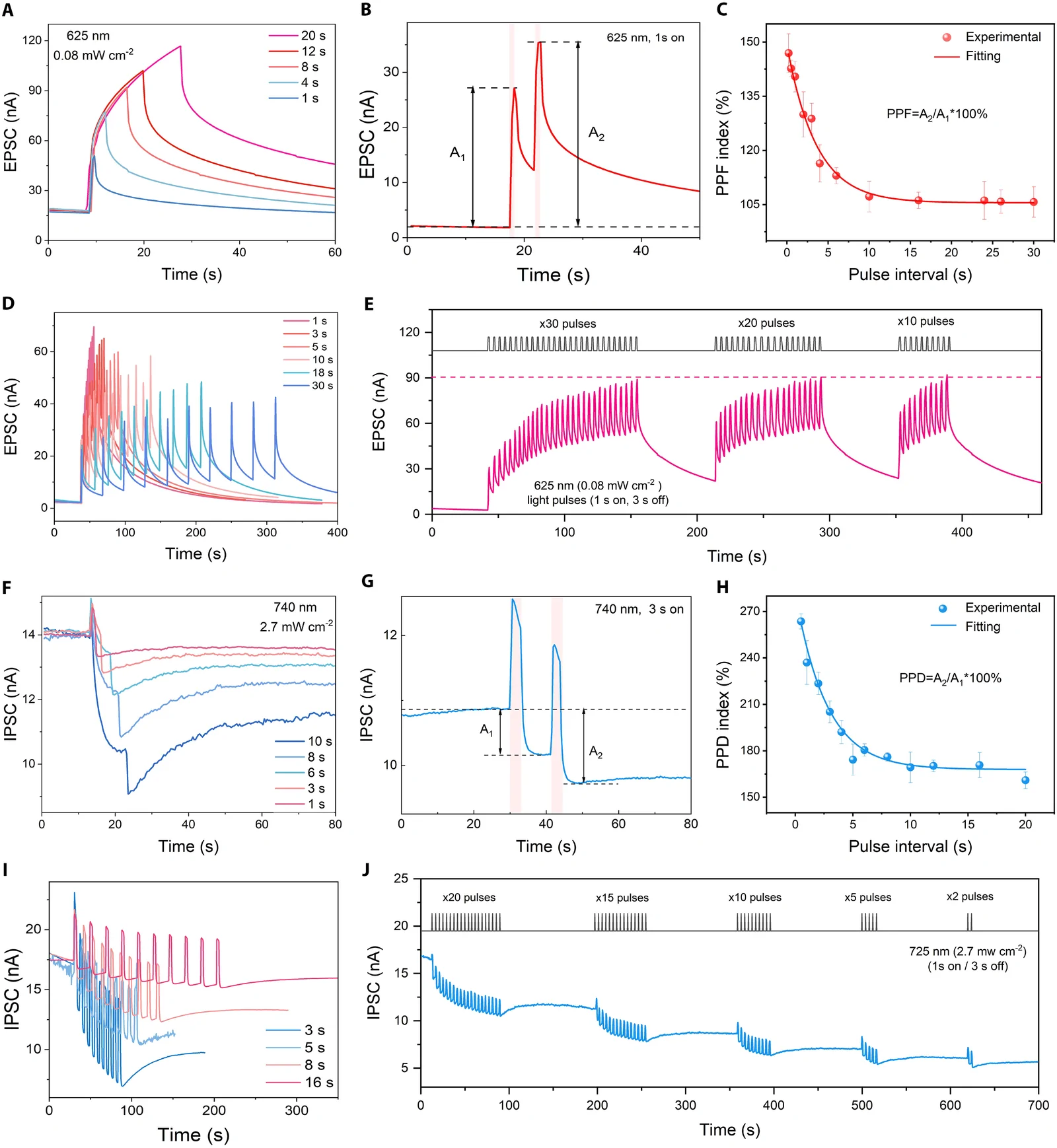
Excitatory and inhibitory synaptic behaviours of PEA_2_SnI_4_/C_60_ device under 625 nm (light intensity: 0.08 mW/cm^2^), and 740 nm light pulses (light intensity: 2.7 mW/cm^2^), respectively (V_DS_ = 1 V, V_G_ = 0 V). (**A**) Excitatory postsynaptic current (EPSC) under single light pulse with different duration time. (**B**) Pair-pulse facilitation (PPF) under two light pulses (1 s on). (**C**) PPF index on different pulse interval. Error bars indicate SD (mean ± SD, *n* = 3). (**D**) 10 light pulses with different time interval (3 s on). (**E**) Learning experience including learning, forgetting, and relearning under light pulses (1 s on, 3 s off). (**F**) Inhibitory postsynaptic current (IPSC) under single light pulse with different duration time. (**G**) Pair-pulse depression (PPD) under two light pulses (3 s on). (**H**) PPD index on different pulse intervals. Error bars indicate SD (mean ± SD, *n* = 3). (**I**) 10 light pulses with different time intervals (3 s on). (**J**) Learning experience including learning, forgetting, and relearning under light pulses (1 s on, 3 s off).

Having elucidated the origin of sub-bandgap photoresponse and wavelength-dependent synaptic behavior, we then investigated the functional emulation of synaptic plasticity using our devices. A hallmark of biological synapses is their bidirectional control of transmission via excitatory and inhibitory signalling, enabling efficient and adaptive information processing. In our system, visible-light (RGB) stimulation elicits an abrupt conductance increase, an EPSC analogous to excitatory neurotransmission, whereas NIR illumination induces a current decrease consistent with an IPSC. Under 625 nm, increasing the light pulse duration can effectively tune the synaptic retention time from a few seconds to several hundred seconds (e.g., 345 s under 20 s illumination, Fig. 3A, fig. S29), closely resembling the temporal window of short-term plasticity (STP) observed in biological neurons (*52*). In contrast, 740 nm NIR produces duration-dependent IPSC suppression with markedly longer memory: a single 20 s pulse yields >8000 s of inhibitory retention (Fig. 3F, fig. S30). To quantify the interval-dependent memory, we evaluated the paired-pulse facilitation (PPF) and depression (PPD) index ($\frac{A_{2}}{A_{1}}\times 100\%$) versus Δt (Fig. 3, B and G), where A_1_ and A_2_ are the current changes in response to the first and second light pulses, respectively. The excitatory PPF yields a facilitation index of 148% under 625 nm illumination (Fig. 3C) and can be well fitted by a single exponential decay function ($\mathrm{PPF}\;\text{index}=Ae^{-∆t/\tau }+C$), where Δ*t* represents the time interval between two consecutive light pulses, A is the amplitude of overall current change, τ is the decay time constant. This single exponential (τ = 3.52 s) indicates a dominant fast relaxation, consistent with band-to-band recombination or release from shallow traps, thus confirming excitation to short-term potentiation. In contrast, the inhibitory PPD shows a high index of 265% (Fig. 3H) and requires a bi-exponential fit with (τ_1_ = 2.26 s, τ_2_ = 8275.05 s) in decay function: $\mathrm{PPD}\;\text{index}=A_{1}e^{-∆t/\tau_{1}}+A_{2}e^{-∆t/\tau_{2}}+C$, revealing the coexistence of fast and slow components. The magnitude of τ_2_ quantitatively rationalizes the long-term depression that is attributable to deep-trap and interfacial charge retention under sub-bandgap NIR photo-response.

For excitatory operation, higher pulse frequency promotes both EPSC/IPSC summation (Fig. 3, D and I), culminating in a post-tetanic potentiation/depression (PTP/PTD = $\left(A_{10}-A_{1}\right)/A_{1}\times 100\%$) index of 260% and 265%, respectively. Spike-number-dependent plasticity (SNDP) reveals a cumulative transition from short-term to long-term, with more light pulses progressively extending retention (figs. S31 and S32). This cumulative transition mirrors the progressive synaptic strengthening and memory consolidation observed in neural circuits, where repetitive presynaptic stimulation reinforces postsynaptic efficacy according to Hebbian learning principles (*53*). As illustrated in Fig. 3E, fewer light pulses are required to recover a previously trained state after repeated learning-forgetting cycles, reflecting a progressive memory consolidation. Specifically, after the initial training with 30 pulses, only 20 pulses were needed to retrieve the memory, and the third learning session required just 10 pulses, demonstrating a light-driven, biologically analogous memory consolidation pathway. Complementarily, inhibitory operation exhibits multi-level depression under trains of identical NIR pulses, with the suppressed conductance persisting without rapid recovery (fig. S33). Consistent with long-term depression characteristics, continuous NIR pulses further suppress the conductance state (Fig. 3J).

One of the most distinctive features of human brain computation is its ability to achieve high-performance information processing at ultra-low energy consumption. To evaluate the energy efficiency of the synaptic device, the energy consumption per spike event was calculated by integrating the transient EPSC response under a constant bias, according to the equation

$$E=V\times \int_{0}^{T}I\left(t\right)\mathrm{dt}$$

where *V* is the voltage bias, and *T* is the spike duration. To investigate the low-voltage operating feasibility of the device, different voltages were applied. As depicted in fig. S34, typical EPSC responses under 625 nm light stimulation can still be reliably elicited with a gradually reduced bias down to 1 mV, whereas IPSC behaviors under 740 nm light remain discernible even at an ultra-low bias of 0.1 mV (fig. S35). The corresponding energy consumption for a single excitatory event and inhibitory event was decreased to 8.9 fJ and 0.99 fJ, respectively, approaching the energy scale of biological synapses (1–10 fJ) and extremely lower than previously reported optical synapses (Fig. 1D). Notably, the particularly low energy consumption of the infrared-driven inhibitory events reflects the advantage of deliberately using sub-bandgap excitation over above-bandgap absorption, as the lower photon energy inherently enables a reduced energy cost per synaptic event. Moreover, the device demonstrates excellent electrical stability, maintaining an on/off current ratio exceeding 10^3^ over 100 consecutive transfer curve cycles (fig. S36). In addition, the EPSC response of the perovskite/C_60_ heterostructure remains highly stable over time, showing negligible degradation after 120 h of storage (fig. S37), in sharp contrast to the pristine perovskite device, which becomes fully degraded after 72 h at the same conditions. These results demonstrate that the heterostructure design enables not only bidirectional synaptic modulation but also substantially improves operational stability via hydrophobic C_60_ protection. This combination of purely optical control, low energy consumption, and stability makes the device a promising candidate for in-sensor computing, particularly in intelligent robot vision, where such integrated optoelectronic synapses could enable efficient, continuous visual perception and adaptive learning directly at the sensory front-end.

### Attention-weight enhanced traffic sign recognition

**Fig. 4.**
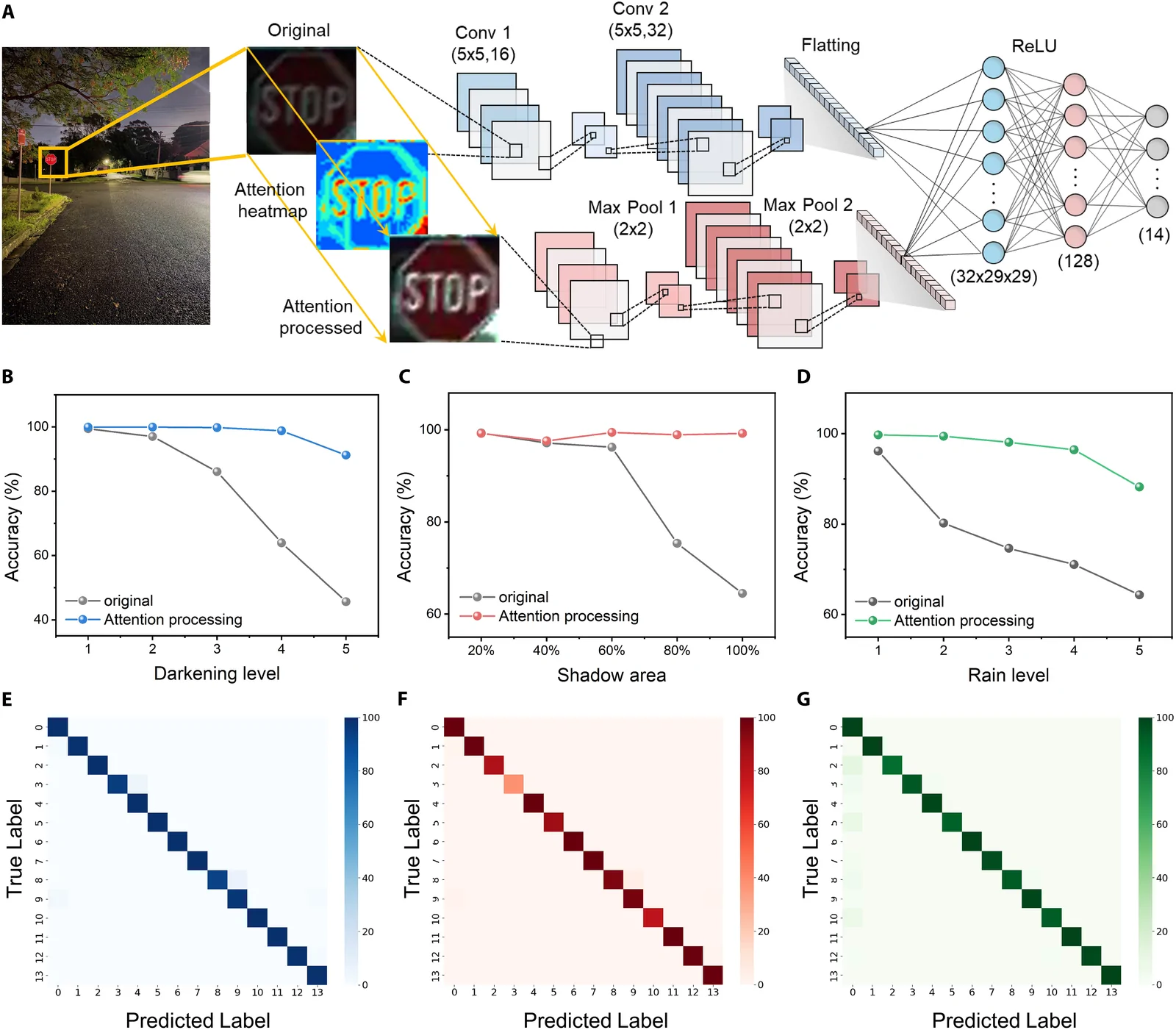
Attention weight enhanced traffic sign recognition. (**A**) The diagram of attention processing and structure of convolutional neural network for recognition task. Images adapted from the CURE-TSR dataset (*77*, *78*). Licensed under CC BY 4.0 (https://creativecommons.org/licenses/by/4.0/deed.en). The recognition accuracy of original traffic sign and the attention-processed traffic sign under different levels of environmental challenges: (**B**) darkening, (**C**) shadow, (**D**) rain. The confusion matrix of attention processed traffic sign under environmental challenges: (**E**) darkening at level 4, (**F**) shadow area at 60%, (**G**) rain at level 4.

In biological visual systems, attention-driven synaptic plasticity is essential for efficiently processing complex visual scenes. Instead of processing all incoming sensory information equally, the brain selectively enhances neural responses to salient regions or features, such as areas with higher brightness or contrast. Neurons corresponding to these attended regions exhibit more vigorous firing activity and form stronger synaptic associations, which amplify the representation of relevant stimuli. Through this mechanism, the visual system can allocate limited computational resources to the most informative inputs, a strategy critical for sustaining high recognition performance in real-world environments, as required in intelligent robotics and autonomous systems. Inspired by this attentional enhancement, we designed a hardware-assisted weighting strategy that employs our perovskite/C_60_ synaptic system in the first layer to pre-amplify salient image features before digital inference. This enables the vision system to prioritize task-relevant information, reducing network complexity and improving robustness and reliability in challenging environments. Specifically, our optoelectronic synaptic device exhibits a linear EPSC response under varying light intensities (fig. S38), analogous to the way human vision responds more strongly to brighter or more prominent cues. The experimentally obtained EPSC values were adopted as the attention weights and applied to traffic sign images from the CURE-TSR dataset (figs. S39 and S40), effectively enhancing salient features. Both the attention-weighted images produced by the perovskite/C_60_ front end and the unweighted baseline images (conventional pipeline) were fed into the Convolutional Neural Networks (CNN) (Fig. 4A). The trained classifier achieved 99% accuracy with ideal and challenge-free image testing sets (fig. S41). To evaluate the robustness of the optoelectronic synaptic system, we simulated five graded levels of environmental degradation, including dark, shadow, and rain scenarios (figs. S42 to S44). In the conventional system without device-assisted attention weighting, recognition accuracy deteriorated substantially with increasing challenge severity, decreasing to 45.6%, 64.5%, and 64.3% under the most severe dark, shadow, and rainy conditions, respectively (Fig. 4, B to D). By contrast, the perovskite/C_60_ optoelectronic synaptic system, endowed with hardware-assisted attention weighting, exhibited markedly enhanced recognition robustness, reaching accuracies of 91.2% in dark (Fig. 4E), 99.2% in shadow (Fig. 4F), and 88.2% in rainy (Fig. 4G) environments.

### Wavelength-dependent plasticity enables efficient physical reservoir classification

**Fig. 5.**
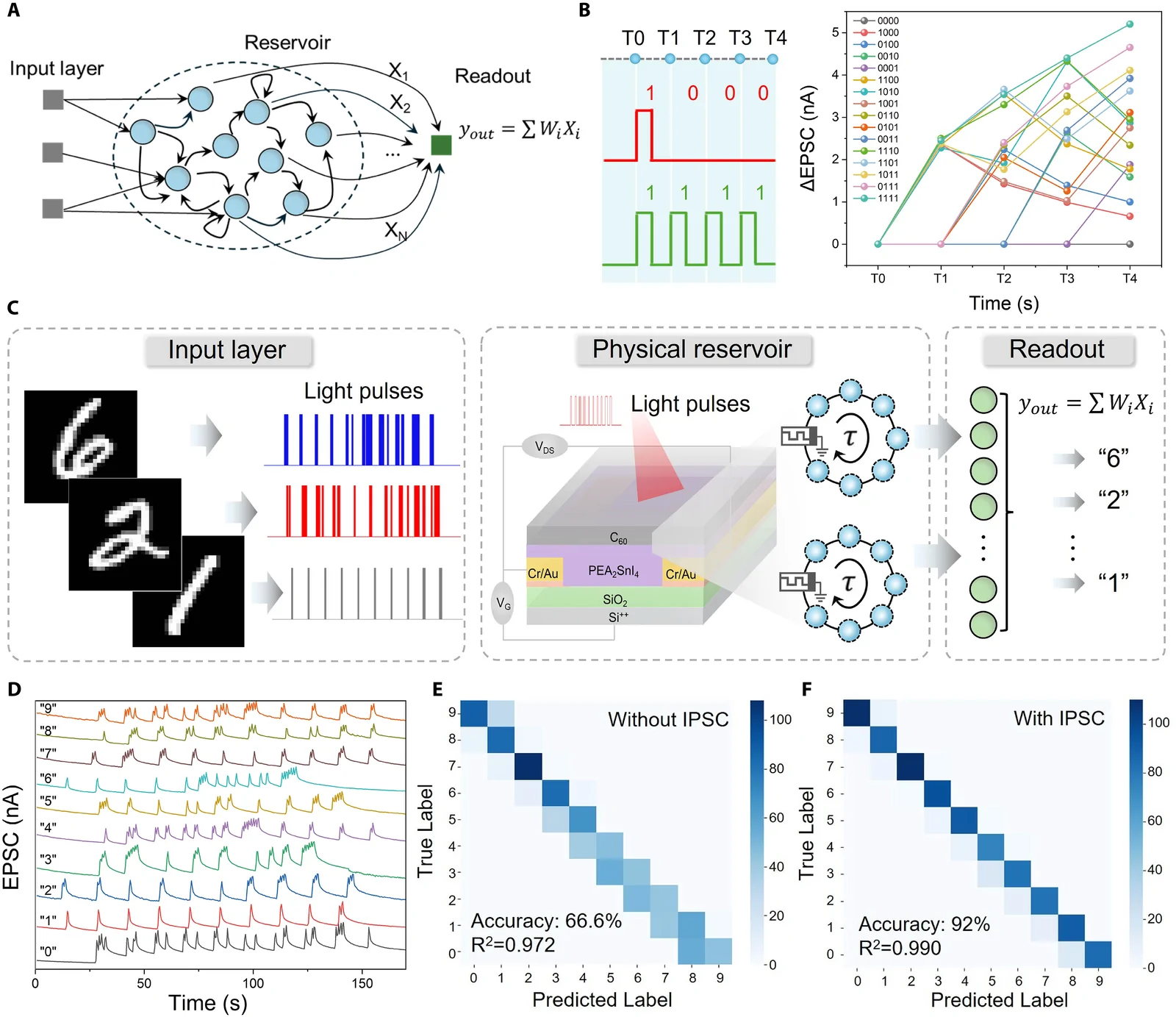
Physical reservoir computing with optoelectronic synapse for image recognition. (**A**) Illustration of physical reservoir computing system. (**B**) Examples to show pulse coding rule and evolution of response EPSC during different pulse streams. (**C**) Process of physical reservoir computing. The input light pulses are coded from MNIST digit figures, then are applied to the synaptic device. The readout results are processed with regression. (**D**) The response current corresponding to 10 different light pulse sequences of the digital images from “0” to “9”. Digital image recognition accuracy without (**E**) and with (**F**) IPSC suppression on background.

In parallel with excitation mechanisms, biological visual systems also rely critically on synaptic suppression to prevent overload from irrelevant or redundant inputs and maintain a high processing efficiency. These complementary excitatory and inhibitory synaptic interactions filter background noise while selectively amplifying salient features, thus enabling efficient extraction of key information from both static scenes and dynamic environments, which is a capability essential for reliable perception in autonomous robots (fig. S45). Translating this concept into hardware, we report a light-programmable physical reservoir based on the optoelectronic synaptic device with wavelength-dependent plasticity to support both excitation and suppression (Fig. 5A). Such a reservoir architecture enables efficient temporal encoding of input stimuli and provides a compact computational framework that performs task-specific learning through simple output readout. To evaluate the physical reservoir’s capability, we first used a simple four-bit binary input space (0000 to 1111), where each bit pattern was encoded into a sequence of short-wavelength optical pulses (Fig. 5B). The resulting synaptic weights exhibited distinct, separable output states for each input pattern, confirming the device’s ability to nonlinearly map input variations into high-dimensional physical states (fig. S46). Building on this preliminary validation, we extended the encoding scheme to more complex input data by converting handwritten digital images into temporally modulated optical sequences (figs. S47 and S48). These image-derived optical pulses triggered excitatory synaptic plasticity across the device, forming a dynamic state representation analogous to memory formation (Fig. 5, C and D). Subsequently, long-wavelength-induced inhibitory plasticity was applied globally to the resulting reservoir readouts, suppressing low-weight or noisy responses, functionally resembling synaptic pruning. This two-stage process enhanced the discriminability of the reservoir output. The final reservoir state was then processed using a simple ridge regression model, which successfully classified digits 0–9. For the perovskite/C_60_ system, conventional reservoir computing without IPSC suppression yielded 66% accuracy (Fig. 5E), while the inclusion of the IPSC phase improved performance to 92% (Fig. 5F), demonstrating the superiority of spectrally decoupled excitation and pruning processing. Notably, the present single-device implementation relies on a time-multiplexed protocol, where spatial pixels are sequentially converted into optical pulses, inevitably introducing serialization latency. Such a latency-complexity trade-off is a known feature of single-node physical reservoir computing (*54*–*56*). In future array-level implementations, this limitation can be mitigated by using spatially distributed nonlinear nodes or parallel optoelectronic synaptic channels to process multiple pixels simultaneously. Combined with ultralow energy consumption of ∼1 fJ for the perovskite/C_60_ device, this highlights the effectiveness of biologically inspired spectral plasticity in enabling efficient, low-latency, on-chip neuromorphic computation through hardware-based reservoir processing.

### Dynamic trajectory detection on a 7 × 7 synaptic array

**Fig. 6.**
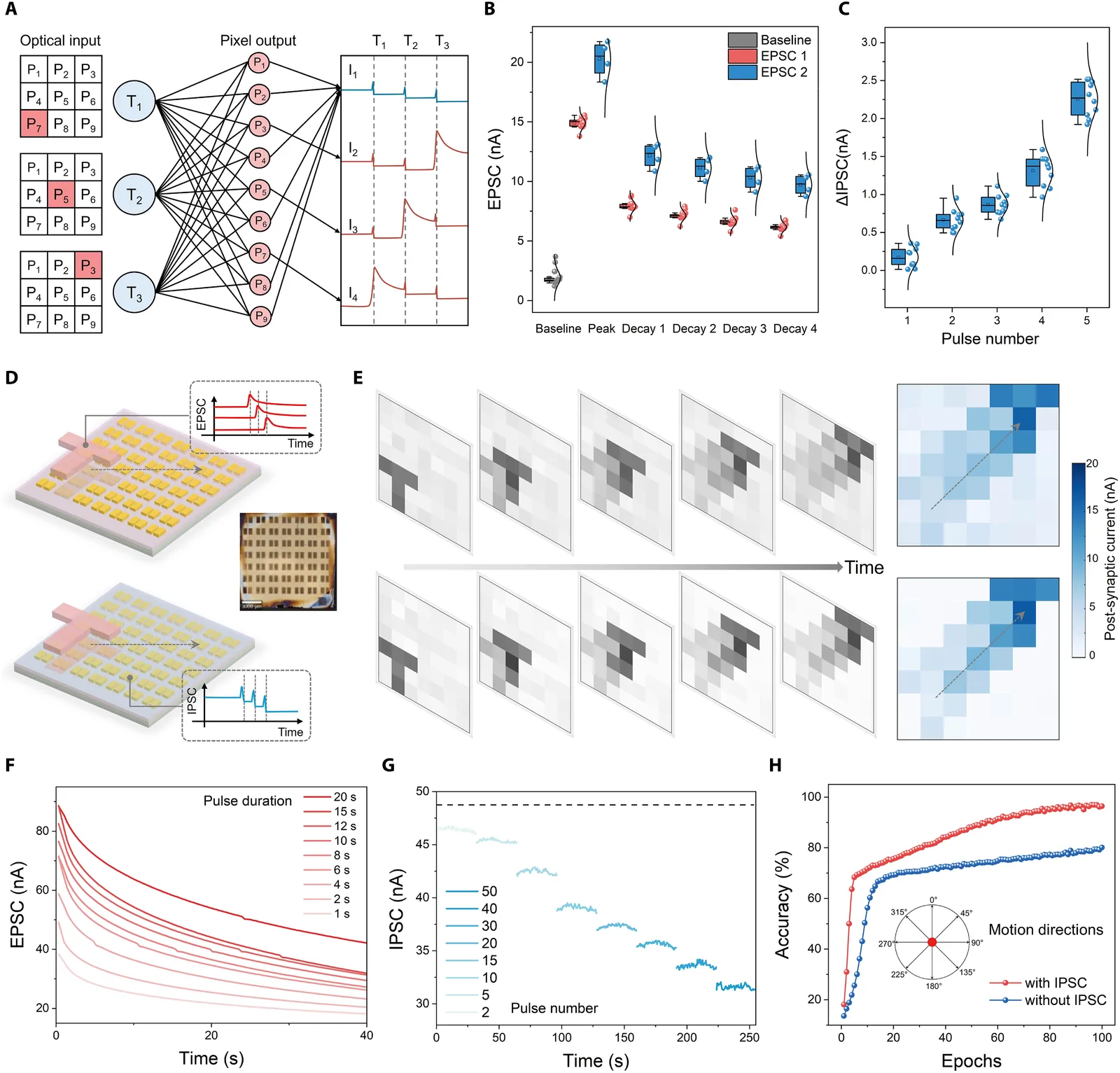
Dynamic trajectory detection with an optoelectronic synapse array. (**A**) Schematic of time-division multiplexed protocol. (**B**) Statistics of the EPSC amplitude across 17 devices under a 3 s, 625 nm pulse, and across 4 devices under a 6 s, 625 nm pulse. (**C**) Statistics of the IPSC change across 10 devices under 2 s on/1 s off, 740 nm inhibition with pulse number of 1–5. (**D**) T-shape motion pattern applied on the array: the target pixels receive 625-nm excitation, while the non-target pixels receive 740-nm NIR inhibition; inset, photograph of the 7 × 7 device array. (**E**) Reconstructed 7 × 7 frames along the T-shape trajectory: top row, excitation only (625 nm); bottom row, with background suppression (740-nm NIR), showing enhanced contrast. (**F**) EPSC relaxation segments following 625 nm pulses of different durations. (**G**) IPSC relaxation segments following 740 nm pulse trains with different pulse counts. (**H**) Simulated motion-direction recognition accuracy on a 28 × 28 array with and without background IPSC suppression; inset, the eight motion directions.

Leveraging the array’s spatiotemporal plasticity under programmable optical drive, we implemented trajectory recognition using a time-division multiplexed protocol (Fig. 6A). In dynamic vision, moving objects can be distinguished from static background through temporal contrast. Specifically, pixels undergoing frame-to-frame intensity variations are identified as motion-salient events, whereas temporally invariant pixels constitute redundant background. To emulate this event-driven processing principle, we generated a time-ordered sequence of event masks from a programmed moving T-shaped object and used these masks to address the 7 × 7 synaptic array. At frame *T*_i_, the pixel *P*_i_ within the event mask was excitatory written by a 625 nm visible pulse, whereas pixels outside the event mask were addressed by 740 nm pulses as a suppressive control input to emulate active background reset. The device-to-device response uniformity was tested on the array under the same stimulus parameters, which shows high consistency (Fig. 6, B and C). A time-ordered stimulus schedule derived from the moving object was assigned to each pixel, and per-pixel readout at a fixed latency yielded frame-resolved conductance maps (Fig. 6D). Within each frame, newly illuminated pixels encoded excitation, while previously activated regions underwent gradual relaxation, producing a spatiotemporally evolving conductance pattern from which the trajectory was inferred (Fig. 6E). Targeted NIR inhibition further enhanced contrast by attenuating unstimulated background, sharpening object contours and improving recognition clarity. Such an array-level implementation of dynamic perception paves the way for low-power, real-time sensing in intelligent mobility systems.

To demonstrate the recognition of real-world object motion direction, an enlarged 28 × 28 synaptic array was further simulated to classify the dynamic object directions, with each synapse corresponding to a pixel. By fitting the relaxation segments of the EPSC curve as shown in Fig. 6F, a function curve representing synapse weight decay over time was obtained, which is correlated with the duration of light pulses and validated with a 95% confidence interval: $I\left(t,t_{0}\right)=\left(0.4449t_{0}+16.693\right)e^{\frac{-t}{4.1305}}+\left(2.2175t_{0}+21.9716\right)e^{\frac{-t}{58.5949}}+7.1117$, where *t*_0_ represents the duration of the light pulse (*57*). The high confidence interval ensures the accuracy and reliability of the model, making the input features in the neural network processing more predictive and stable. This feature was used to construct object motion in the array model. The relaxation segments of the IPSC under NIR pulses with different pulse numbers are shown in Fig. 6G, which was used to simulate signal suppression in the human vision system for a static background. A dataset consisting of 30000 samples representing eight moving directions (0°, 45°, 90°, 135°, 180°, 225°, 270°, 315°) was created and divided into training and testing sets in an 8:2 ratio. The baseline of the synaptic current was set to be the background noise. Using this data, the output synaptic current of the array during 5-step object motion was simulated with and without IPSC suppression. Subsequently, these signals were trained by a five-layer artificial neural network. After 100 training epochs, the classification accuracy of the motion direction recognition increased from 80% without IPSC to 97.1% with IPSC (Fig. 6H). The combination of spatially selective excitation and background suppression within the array embodies a form of sparse and efficient coding, echoing biological strategies for motion tracking.

## DISCUSSION

In summary, we report an optoelectronic synapse based on a lead-free 2D halide perovskite PEA_2_SnI_4_/C_60_ heterojunction that exhibits unique wavelength-dependent excitatory and inhibitory plasticity. The device exhibits long conductance retention (∼350 s for potentiation and >8000 s for depression) while operating at an ultralow energy of ∼1 fJ per synaptic event, approaching the efficiency of biological synapses. Mechanistically, we attribute the bidirectional response to sub-bandgap absorption mediated by deep in-gap states together with asymmetric interfacial charge-transfer dynamics across the heterojunction. We demonstrate that C_60_ can function not merely as a passive electron-accepting layer, but as an actively programmable interface, in which sub-bandgap excitation unlocks distinct interfacial charge-transfer pathways and achieves wavelength-selective inhibition. This wavelength-dependent control converts sub-bandgap absorption from an unintended effect into an active control handle for precise and reversible synaptic modulation. More broadly, our work paves the way for exploiting wide-bandgap, lead-free 2D tin perovskites as a viable platform for narrow-bandgap and even NIR optoelectronic functionalities, extending the potential applications while taking advantage of superior stability over their 3D counterparts.

Leveraging this spectrally decoupled excitatory-inhibitory dual mode, the proposed optoelectronic synapse enables device-level implementation of biologically inspired attention, suppression, and adaptive learning within a unified platform. Importantly, the wavelength-encoded balance between potentiation and depression provides a compact and interpretable means to regulate synaptic weights, offering a viable route toward scalable, energy-efficient in-sensor computing architectures. Building on this capability, we demonstrate a unified neuromorphic-vision workflow that spans attention-enhanced traffic sign recognition, reservoir computing digit classification, and frame-resolved spatiotemporal trajectory sensing on a device array. By co-locating sensing and processing in an ultralow-power element, this work advances the hardware foundation for neuromorphic vision in autonomous vehicles, wearable electronics, and intelligent edge systems operating under complex conditions.

## MATERIALS AND METHODS

### Materials

Iodine (I_2_, 99.7%), tin powder (Sn, 99.8%), dimethyl formamide (DMF, anhydrous, 99.8%), dimethyl sulfoxide (DMSO, anhydrous, 99.9%), and L-glutathione (GSH, 98%) were purchased from Sigma-Aldrich. Phenethylammonium iodide (PEAI) was purchased from Greatcell Solar. Fullerene (C_60_) were purchased from 1-Material. P-type doped silicon wafers (<0.0015 Ω·cm) with thermally grown 100 nm oxide layer were purchased from AME Energy. All the chemicals were used as received without further purification.

### Preparation of the perovskite precursor solution

I_2_ (0.15 mmol, 38.07 mg) was dissolved in DMF/DMSO (v/v = 4:1, 1.5 ml) with an excess amount of Sn powder added. The solution was kept stirring until the color changed to pale yellow indicating the conversion into SnI_2_. PEAI (0.3 mmol, 74.73 mg) and GSH (3 mg) were dissolved in the as prepared SnI_2_ solution to yield PEA_2_SnI_4_ precursor (0.1 M, 1.5 ml). The precursor was filtered with 0.22 μm polytetrafluoroethylene (PTFE) filters before spin coating.

### Film deposition and device assembly

Contacts were deposited on SiO_2_/Si wafer by sequentially evaporating Cr (5 nm) and Au (35 nm) through a shadow mask with a channel width and length of 1000 μm and 200 μm respectively. The substrates were then cleaned by sonicating in ethanol, acetone, and isopropanol for 15 min each. The substrate surface was exposed to UV-ozone for 20 min before transferring into the N_2_ glovebox for spin coating. For the film deposition, 40 μl of the precursor solution was dropped onto the substrate, which was rotated at 5000 rpm for 60 sec with a ramp of 1000 rpm/s. After 20 sec, 200 μl of toluene was dropped on the substrate. The films were annealed at 90°C for 10 min. For the PEA_2_SnI_4_/C_60_ heterostructure, C_60_ (10 nm) were thermally evaporated onto the PEA_2_SnI_4_ film under vacuum of 3 × 10^−6^ Torr.

### Film characterizations

Steady state PL was measured with Horiba FluoroMax Plus. UV-Vis-NIR absorption spectra were obtained using Shimadzu UV-3600i Plus. XRD measurements were conducted with Cu Kα radiation using Rigaku MiniFlex with scan speed of 5°/min. AFM measurements were taken using Bruker Dimension Icon. The SEM images were taken using JEOL JSM-7100F with an acceleration voltage of 3 kV. XPS and UPS spectra were measured by Kratos Axis Supra Plus XPS with an Al Kα radiation source, and the spectra were calibrated by using C 1 s (284.8 eV) as a reference.

The UPS spectra were used to evaluate the energy levels of work function (WF), valence band (VBM), and conduction band (CBM) using the following formula, where hν represents the radiative photon energy of He I α (21.22 eV) and E_g_ represents the bandgap.

$$\mathrm{WF}=\mathrm{h\nu}-E_{\text{cutoff}}$$

$$\mathrm{VBM}=-\mathrm{WF}-E_{\text{onset}}$$

$$\mathrm{CBM}=E_{g}+\mathrm{VBM}$$

### Kelvin probe force microscopy (KPFM) measurements

AIST-NT SmartSPM 1000 system has been used to perform the KPFM measurements on 2D perovskite thin films. All the KPFM measurements were performed using the platinum coated AFM tip (Mikromasch HQ:NSC35/pt) which has a 5.4 N/m force constant and approximately 150 kHz resonance frequency. To minimize the external influence and degradation of the film, a controlled nitrogen stream has been used during the measurements. Moreover, amplitude modulation was used to obtain simultaneously surface topography and CPD maps and to obtain accurate images, a 30 nm distance has been maintained between the sample and the tip. A slow scanning rate of 1 Hz with 256 × 256-pixel size was used to obtain high resolution images of the sample topography and CPD. For the light dependent KPFM measurements, an external light source (CNI FC-41) with multiwavelength has been used. The light source was positioned at 30° relative to the sample surface to avoid the shadow while performing the measurements. KPFM measurements were performed on various regions on the same sample to check the data reproducibility.

### Device measurements

The electrical outputs of the devices were systematically recorded using a semiconductor parameter analyzer (Keithley 4200). All the measurements were performed in vacuum environments (<10^−5^ torr) provided in a probe station. The light sources were equipped with optical fiber (Thorlabs).

## Supplementary Materials

### This PDF file includes:

Supplementary Text 1 to 4 Figs. S1 to S48 Tables S1 to S4 References

## References

[1] P. Slade, C. Atkeson, J. M. Donelan, H. Houdijk, K. A. Ingraham, M. Kim, K. Kong, K. L. Poggensee, R. Riener, M. Steinert, J. Zhang, S. H. Collins, On human-in-the-loop optimization of human-robot interaction. Nature 633, 779–788 (2024). 39322732 10.1038/s41586-024-07697-2

[2] C. A. Aubin, B. Gorissen, E. Milana, P. R. Buskohl, N. Lazarus, G. A. Slipher, C. Keplinger, J. Bongard, F. Iida, J. A. Lewis, R. F. Shepherd, Towards enduring autonomous robots via embodied energy. Nature 602, 393–402 (2022). 35173338 10.1038/s41586-021-04138-2

[3] H. Wang, L. Kong, Z. Fang, R. Zou, Z. Zhang, X. Zhao, Q. Zong, Z. Xie, Symbiotic energy paradigm for self-sustaining aerial robots. Nat. Rev. Electr. Eng. 2, 302–319 (2025).

[4] H. Huang, X. Liang, Y. Wang, J. Tang, Y. Li, Y. Du, W. Sun, J. Zhang, P. Yao, X. Mou, F. Xu, J. Zhang, Y. Lu, Z. Liu, J. Wang, Z. Jiang, R. Hu, Z. Wang, Q. Zhang, B. Gao, X. Bai, L. Fang, Q. Dai, H. Yin, H. Qian, H. Wu, Fully integrated multi-mode optoelectronic memristor array for diversified in-sensor computing. Nat. Nanotechnol. 20, 93–103 (2025). 39516386 10.1038/s41565-024-01794-z

[5] N. J. Tye, S. Hofmann, P. Stanley-Marbell, Materials and devices as solutions to computational problems in machine learning. Nat. Electron. 6, 479–490 (2023).

[6] F. Zhou, Y. Chai, Near-sensor and in-sensor computing. Nat. Electron. 3, 664–671 (2020).

[7] Z. Wang, T. Wan, S. Ma, Y. Chai, Multidimensional vision sensors for information processing. Nat. Nanotechnol. 19, 919–930 (2024). 38877323 10.1038/s41565-024-01665-7

[8] K. Roy, A. Jaiswal, P. Panda, Towards spike-based machine intelligence with neuromorphic computing. Nature 575, 607–617 (2019). 31776490 10.1038/s41586-019-1677-2

[9] L. Mennel, J. Symonowicz, S. Wachter, D. K. Polyushkin, A. J. Molina-Mendoza, T. Mueller, Ultrafast machine vision with 2D material neural network image sensors. Nature 579, 62–66 (2020). 32132692 10.1038/s41586-020-2038-x

[10] J. Chen, Z. Zhou, B. J. Kim, Y. Zhou, Z. Wang, T. Wan, J. Yan, J. Kang, J.-H. Ahn, Y. Chai, Optoelectronic graded neurons for bioinspired in-sensor motion perception. Nat. Nanotechnol. 18, 882–888 (2023). 37081081 10.1038/s41565-023-01379-2

[11] Y. Chai, In-sensor computing for machine vision. Nature 579, 32–33 (2020). 32132685 10.1038/d41586-020-00592-6

[12] Y. Hou, J. Li, J. Yoon, A. M. Knoepfel, D. Yang, L. Zheng, T. Ye, S. Ghosh, S. Priya, K. Wang, Retina-inspired narrowband perovskite sensor array for panchromatic imaging. Sci. Adv. 9, eade2338 (2023). 37058567 10.1126/sciadv.ade2338 PMC10104461

[13] Z. Long, X. Qiu, C. L. J. Chan, Z. Sun, Z. Yuan, S. Poddar, Y. Zhang, Y. Ding, L. Gu, Y. Zhou, W. Tang, A. K. Srivastava, C. Yu, X. Zou, G. Shen, Z. Fan, A neuromorphic bionic eye with filter-free color vision using hemispherical perovskite nanowire array retina. Nat. Commun. 14, 1972 (2023). 37031227 10.1038/s41467-023-37581-y PMC10082761

[14] R. A. John, Y. Demirağ, Y. Shynkarenko, Y. Berezovska, N. Ohannessian, M. Payvand, P. Zeng, M. I. Bodnarchuk, F. Krumeich, G. Kara, I. Shorubalko, M. V. Nair, G. A. Cooke, T. Lippert, G. Indiveri, M. V. Kovalenko, Reconfigurable halide perovskite nanocrystal memristors for neuromorphic computing. Nat. Commun. 13, 2074 (2022). 35440122 10.1038/s41467-022-29727-1 PMC9018677

[15] H. Cui, Y. Xiao, Y. Yang, M. Pei, S. Ke, X. Fang, L. Qiao, K. Shi, H. Long, W. Xu, P. Cai, P. Lin, Y. Shi, Q. Wan, C. Wan, A bioinspired in-materia analog photoelectronic reservoir computing for human action processing. Nat. Commun. 16, 2263 (2025). 40050621 10.1038/s41467-025-56899-3 PMC11885466

[16] F. Zhou, Z. Zhou, J. Chen, T. H. Choy, J. Wang, N. Zhang, Z. Lin, S. Yu, J. Kang, H. S. P. Wong, Y. Chai, Optoelectronic resistive random access memory for neuromorphic vision sensors. Nat. Nanotechnol. 14, 776–782 (2019). 31308498 10.1038/s41565-019-0501-3

[17] K. Chen, H. Hu, I. Song, H. B. Gobeze, W.-J. Lee, A. Abtahi, K. S. Schanze, J. Mei, Organic optoelectronic synapse based on photon-modulated electrochemical doping. Nat. Photonics 17, 629–637 (2023).

[18] B. Pradhan, S. Das, J. Li, F. Chowdhury, J. Cherusseri, D. Pandey, D. Dev, A. Krishnaprasad, E. Barrios, A. Towers, A. Gesquiere, L. Tetard, T. Roy, J. Thomas, Ultrasensitive and ultrathin phototransistors and photonic synapses using perovskite quantum dots grown from graphene lattice. Sci. Adv. 6, eaay5225 (2020). 32095529 10.1126/sciadv.aay5225 PMC7015692

[19] L. Wang, H. Wang, J. Liu, Y. Wang, H. Shao, W. Li, M. Yi, H. Ling, L. Xie, W. Huang, Negative photoconductivity transistors for visuomorphic computing. Adv. Mater. 36, e2403538 (2024). 39040000 10.1002/adma.202403538

[20] Z. Wang, L. Lu, J. Meng, T. Wang, Emerging negative photoconductivity effect-based synaptic device for optoelectronic in-sensor computing. Adv. Mater. 37, e2504710 (2025). 40417972 10.1002/adma.202504710

[21] Y. Wang, Y. Zha, C. Bao, F. Hu, Y. Di, C. Liu, F. Xing, X. Xu, X. Wen, Z. Gan, B. Jia, Monolithic 2D perovskites enabled artificial photonic synapses for neuromorphic vision sensors. Adv. Mater. 36, e2311524 (2024). 38275007 10.1002/adma.202311524

[22] T. Liu, Z. Yuan, L. Wang, C. Shan, Q. Zhang, H. Chen, H. Wang, W. Wu, L. Huang, Y. Chai, X. Meng, Chelated tin halide perovskite for near-infrared neuromorphic imaging array enabling object recognition and motion perception. Nat. Commun. 16, 4261 (2025). 40335551 10.1038/s41467-025-59624-2 PMC12059062

[23] L. Guo, H. Sun, L. Min, M. Wang, F. Cao, L. Li, Two-terminal perovskite optoelectronic synapse for rapid trained neuromorphic computation with high accuracy. Adv. Mater. 36, e2402253 (2024). 38553842 10.1002/adma.202402253

[24] F. Zhang, M. Shao, C. Wang, W. Wen, W. Shi, M. Qin, H. Huang, X. Wei, Y. Guo, Y. Liu, Photoinduced nonvolatile memory transistor based on lead-free perovskite incorporating fused π-conjugated organic ligands. Adv. Mater. 36, e2307326 (2024). 37849381 10.1002/adma.202307326

[25] Z. Guo, J. Zhang, B. Yang, L. Li, X. Liu, Y. Xu, Y. Wu, P. Guo, T. Sun, S. Dai, H. Liang, J. Wang, Y. Zou, L. Xiong, J. Huang, Organic high-temperature synaptic phototransistors for energy-efficient neuromorphic computing. Adv. Mater. 36, e2310155 (2024). 38100140 10.1002/adma.202310155

[26] Y. Lian, J. Han, M. Yang, S. Peng, C. Zhang, C. Han, X. Zhang, X. Liu, H. Zhou, Y. Wang, C. Lan, J. Gou, Y. Jiang, Y. Liao, H. Yu, J. Wang, Tunable bi-directional photoresponse in hybrid PtSe_2-*x*_ thin films based on precisely controllable selenization engineering. Adv. Funct. Mater. 32, 2205709 (2022).

[27] B. Ouyang, J. Wang, G. Zeng, J. Yan, Y. Zhou, X. Jiang, B. Shao, Y. Chai, Bioinspired in-sensor spectral adaptation for perceiving spectrally distinctive features. Nat. Electron. 7, 705–713 (2024).

[28] C.-M. Yang, T.-C. Chen, D. Verma, L.-J. Li, B. Liu, W.-H. Chang, C.-S. Lai, Bidirectional all-optical synapses based on a 2D Bi_2_O_2_Se/graphene hybrid structure for multifunctional optoelectronics. Adv. Funct. Mater. 30, 2001598 (2020).

[29] Y. Wang, Y. Zhu, Y. Li, Y. Zhang, D. Yang, X. Pi, Dual-modal optoelectronic synaptic devices with versatile synaptic plasticity. Adv. Funct. Mater. 32, 2107973 (2022).

[30] B. Cai, Y. Huang, L. Tang, T. Wang, C. Wang, Q. Sun, D. W. Zhang, L. Chen, All-optically controlled retinomorphic memristor for image processing and stabilization. Adv. Funct. Mater. 33, 2306272 (2023).

[31] W. Bai, M. Liang, T. Xuan, T. Gong, L. Bian, H. Li, R.-J. Xie, Ligand engineering enables efficient pure red tin-based perovskite light-emitting diodes. Angew. Chem. Int. Ed. 135, e202312728 (2023). 10.1002/anie.202312728 37888877

[32] H. Zhou, H. Rao, Y. Jin, W. Zhu, Z. Deng, Y. Zhong, W. Sheng, G. Liu, L. Tan, Y. Chen, Controlled crystallization kinetics via conjugated organic spacers enables ordered epitaxial growth of two-step deposited tin-based perovskite solar cells. Angew. Chem. Int. Ed. 64, e202513416 (2025). 10.1002/anie.202513416 40711385

[33] S. Zhang, M. Qu, J. Duan, H. Dai, T. Xuan, R. Xie, H. Li, Improving the performance of pure-red 2D tin-based perovskite light-emitting diodes through N-Methylthiourea ligand engineering. J. Mater. Chem. C 12, 14368–14375 (2024).

[34] S. You, H. Zhu, Z. Shen, X. Wang, B. Shao, Q. Wang, J. Lu, Y. Yuan, B. D. Dou, E. M. Sanehira, T. Russell, A. Lorenz, Y. Dong, L. Chen, M. Casareto, N. Rolston, M. C. Beard, J. J. Berry, M. Freitag, Y. Yan, O. M. Bakr, K. Zhu, C_60_-based ionic salt electron shuttle for high-performance inverted perovskite solar modules. Science 388, 964–968 (2025). 40245190 10.1126/science.adv4701

[35] Y.-T. Yang, Y.-H. Shih, Q.-G. Chen, C.-H. Chen, M.-H. Yu, C.-H. Nieh, B.-H. Lin, W.-C. Chen, W.-Y. Lee, C.-C. Chueh, Revealing the potential of perovskite transistors for dual-modulated synaptic behavior through heterojunction design. ACS Energy Lett. 9, 4564–4571 (2024).

[36] F. Yuan, X. Zheng, A. Johnston, Y.-K. Wang, C. Zhou, Y. Dong, B. Chen, H. Chen, J. Z. Fan, G. Sharma, P. Li, Y. Gao, O. Voznyy, H.-T. Kung, Z.-H. Lu, O. M. Bakr, E. H. Sargent, Color-pure red light-emitting diodes based on two-dimensional lead-free perovskites. Sci. Adv. 6, eabb0253 (2020). 33055155 10.1126/sciadv.abb0253 PMC7556835

[37] X. Huang, Q. Li, W. Shi, K. Liu, Y. Zhang, Y. Liu, X. Wei, Z. Zhao, Y. Guo, Y. Liu, Dual-mode learning of ambipolar synaptic phototransistor based on 2D perovskite/organic heterojunction for flexible color recognizable visual system. Small 17, e2102820 (2021). 34319659 10.1002/smll.202102820

[38] S. B. Anantharaman, J. Lynch, C. E. Stevens, C. Munley, C. Li, J. Hou, H. Zhang, A. Torma, T. Darlington, F. Coen, K. Li, A. Majumdar, P. J. Schuck, A. Mohite, H. Harutyunyan, J. R. Hendrickson, D. Jariwala, Dynamics of self-hybridized exciton–polaritons in 2D halide perovskites. Light Sci. Appl. 13, 1 (2024). 38161209 10.1038/s41377-023-01334-9 PMC10757995

[39] E. Choi, Y. Zhang, A. M. Soufiani, M. Lee, R. F. Webster, M. E. Pollard, P. J. Reece, W. Lee, J. Seidel, J. Lim, J.-H. Yun, J. S. Yun, Exploration of sub-bandgap states in 2D halide perovskite single-crystal photodetector. npj 2D Mater. Appl. 6, 43 (2022).

[40] J. Zhang, X. Zhu, M. Wang, B. Hu, Establishing charge-transfer excitons in 2D perovskite heterostructures. Nat. Commun. 11, 2618 (2020). 32457289 10.1038/s41467-020-16415-1 PMC7250833

[41] Y. Meng, F. Li, C. Lan, X. Bu, X. Kang, R. Wei, S. Yip, D. Li, F. Wang, T. Takahashi, T. Hosomi, K. Nagashima, T. Yanagida, J. C. Ho, Artificial visual systems enabled by quasi–two-dimensional electron gases in oxide superlattice nanowires. Sci. Adv. 6, eabc6389 (2020). 33177088 10.1126/sciadv.abc6389 PMC7673733

[42] J. Pan, Y. Wu, X. Zhang, J. Chen, J. Wang, S. Cheng, X. Wu, X. Zhang, J. Jie, Anisotropic charge trapping in phototransistors unlocks ultrasensitive polarimetry for bionic navigation. Nat. Commun. 13, 6629 (2022). 36333339 10.1038/s41467-022-34421-3 PMC9636252

[43] T. Ochiai, T. Akazawa, Y. Miyatake, K. Sumita, S. Ohno, S. Monfray, F. Boeuf, K. Toprasertpong, S. Takagi, M. Takenaka, Ultrahigh-responsivity waveguide-coupled optical power monitor for Si photonic circuits operating at near-infrared wavelengths. Nat. Commun. 13, 7443 (2022). 36494365 10.1038/s41467-022-35206-4 PMC9734153

[44] F. Zhou, I. Abdelwahab, K. Leng, K. P. Loh, W. Ji, 2D perovskites with giant excitonic optical nonlinearities for high-performance sub-bandgap photodetection. Adv. Mater. 31, e1904155 (2019). 31592567 10.1002/adma.201904155

[45] D. Meggiolaro, S. G. Motti, E. Mosconi, A. J. Barker, J. Ball, C. A. R. Perini, F. Deschler, A. Petrozza, F. De Angelis, Iodine chemistry determines the defect tolerance of lead-halide perovskites. Energ. Environ. Sci. 11, 702–713 (2018).

[46] D. J. Keeble, J. Wiktor, S. K. Pathak, L. J. Phillips, M. Dickmann, K. Durose, H. J. Snaith, W. Egger, Identification of lead vacancy defects in lead halide perovskites. Nat. Commun. 12, 5566 (2021). 34552098 10.1038/s41467-021-25937-1 PMC8458286

[47] X. Zhang, M. E. Turiansky, J.-X. Shen, C. G. Van de Walle, Iodine interstitials as a cause of nonradiative recombination in hybrid perovskites. Phys. Rev. B 101, 140101 (2020).

[48] G. J. W. Aalbers, T. P. A. van der Pol, K. Datta, W. H. M. Remmerswaal, M. M. Wienk, R. A. J. Janssen, Effect of sub-bandgap defects on radiative and non-radiative open-circuit voltage losses in perovskite solar cells. Nat. Commun. 15, 1276 (2024). 38341428 10.1038/s41467-024-45512-8 PMC10858920

[49] T. Lu, Z. Dang, Y. Luo, Y. Li, X. Rao, Z. Zhang, Y. Hu, Z. Chen, Z. Li, U. Petralanda, J. Xie, J. Yu, P. Gao, Lattice expansion of hybrid perovskite inhibits halogen interstitial generation and enhances solar cell performance. Nat. Commun. 16, 8591 (2025). 41022831 10.1038/s41467-025-63693-8 PMC12480675

[50] O. J. Sandberg, C. Kaiser, S. Zeiske, N. Zarrabi, S. Gielen, W. Maes, K. Vandewal, P. Meredith, A. Armin, Mid-gap trap state-mediated dark current in organic photodiodes. Nat. Photonics 17, 368–374 (2023).

[51] N. Zarrabi, O. J. Sandberg, S. Zeiske, W. Li, D. B. Riley, P. Meredith, A. Armin, Charge-generating mid-gap trap states define the thermodynamic limit of organic photovoltaic devices. Nat. Commun. 11, 5567 (2020). 33149193 10.1038/s41467-020-19434-0 PMC7642445

[52] R. S. Zucker, W. G. Regehr, Short-term synaptic plasticity. Annu. Rev. Physiol. 64, 355–405 (2002). 11826273 10.1146/annurev.physiol.64.092501.114547

[53] J. Ko, C. Ock, H. Gim, K. Hong, Y. Lee, K. C. Kwon, Two-dimensional materials for artificial sensory devices: Advancing neuromorphic sensing technology. npj 2D Mater. Appl. 9, 35 (2025).

[54] R. Chen, H. Yang, R. Li, G. Yu, Y. Zhang, J. Dong, D. Han, Z. Zhou, P. Huang, L. Liu, X. Liu, J. Kang, Thin-film transistor for temporal self-adaptive reservoir computing with closed-loop architecture. Sci. Adv. 10, eadl1299 (2024). 38363846 10.1126/sciadv.adl1299 PMC10871528

[55] S. Stepney, Physical reservoir computing: A tutorial. Nat. Comput. 23, 665–685 (2024).

[56] Y. Zhong, J. Tang, X. Li, B. Gao, H. Qian, H. Wu, Dynamic memristor-based reservoir computing for high-efficiency temporal signal processing. Nat. Commun. 12, 408 (2021). 33462233 10.1038/s41467-020-20692-1 PMC7814066

[57] H. Zhang, S. Wang, L. Wang, S. Li, H. Liu, X. Zhu, Y. Chen, G. Xu, M. Zhang, Q. Liu, R. Wang, K. Xiao, Bio-inspired retina by regulating ion-confined transport in hydrogels. Adv. Mater. 37, e2500809 (2025). 40072321 10.1002/adma.202500809

[58] J. Sun, S. Oh, Y. Choi, S. Seo, M. J. Oh, M. Lee, W. B. Lee, P. J. Yoo, J. H. Cho, J.-H. Park, Optoelectronic synapse based on IGZO-alkylated graphene oxide hybrid structure. Adv. Funct. Mater. 28, 1804397 (2018).

[59] Y. Wang, Z. Lv, J. Chen, Z. Wang, Y. Zhou, L. Zhou, X. Chen, S.-T. Han, Photonic synapses based on inorganic perovskite quantum dots for neuromorphic computing. Adv. Mater. 30, 1802883 (2018). 10.1002/adma.201802883 30063261

[60] J. J. Yu, L. Y. Liang, L. X. Hu, H. X. Duan, W. H. Wu, H. L. Zhang, J. H. Gao, F. Zhuge, T. C. Chang, H. T. Cao, Optoelectronic neuromorphic thin-film transistors capable of selective attention and with ultra-low power dissipation. Nano Energy 62, 772–780 (2019).

[61] N. Duan, Y. Li, H.-C. Chiang, J. Chen, W.-Q. Pan, Y.-X. Zhou, Y.-C. Chien, Y.-H. He, K.-H. Xue, G. Liu, T.-C. Chang, X.-S. Miao, An electro-photo-sensitive synaptic transistor for edge neuromorphic visual systems. Nanoscale 11, 17590–17599 (2019). 31461106 10.1039/c9nr04195h

[62] K. Wang, S. Dai, Y. Zhao, Y. Wang, C. Liu, J. Huang, Light-stimulated synaptic transistors fabricated by a facile solution process based on inorganic perovskite quantum dots and organic semiconductors. Small 15, e1900010 (2019). 30740892 10.1002/smll.201900010

[63] L. Yin, C. Han, Q. Zhang, Z. Ni, S. Zhao, K. Wang, D. Li, M. Xu, H. Wu, X. Pi, D. Yang, Synaptic silicon-nanocrystal phototransistors for neuromorphic computing. Nano Energy 63, 103859 (2019).

[64] H.-L. Park, H. Kim, D. Lim, H. Zhou, Y.-H. Kim, Y. Lee, S. Park, T.-W. Lee, Retina-inspired carbon nitride-based photonic synapses for selective detection of UV light. Adv. Mater. 32, e1906899 (2020). 31984573 10.1002/adma.201906899

[65] L. Yin, W. Huang, R. Xiao, W. Peng, Y. Zhu, Y. Zhang, X. Pi, D. Yang, Optically stimulated synaptic devices based on the hybrid structure of silicon nanomembrane and perovskite. Nano Lett. 20, 3378–3387 (2020). 32212734 10.1021/acs.nanolett.0c00298

[66] Z. Liu, S. Dai, Y. Wang, B. Yang, D. Hao, D. Liu, Y. Zhao, L. Fang, Q. Ou, S. Jin, J. Zhao, J. Huang, Photoresponsive transistors based on lead-free perovskite and carbon nanotubes. Adv. Funct. Mater. 30, 1906335 (2020).

[67] T. Chen, X. Wang, D. Hao, S. Dai, Q. Ou, J. Zhang, J. Huang, Photonic synapses with ultra-low energy consumption based on vertical organic field-effect transistors. Adv. Opt. Mater. 9, 2002030 (2021).

[68] J. Shi, J. Jie, W. Deng, G. Luo, X. Fang, Y. Xiao, Y. Zhang, X. Zhang, X. Zhang, A fully solution-printed photosynaptic transistor array with ultralow energy consumption for artificial-vision neural networks. Adv. Mater. 34, e2200380 (2022). 35243701 10.1002/adma.202200380

[69] Y. Cao, X. Sha, X. Bai, Y. Shao, Y. Gao, Y.-M. Wei, L. Meng, N. Zhou, J. Liu, B. Li, X.-F. Yu, J. Li, Ultralow light-power consuming photonic synapses based on ultrasensitive perovskite/indium-gallium-zinc-oxide heterojunction phototransistors. Adv. Electron. Mater. 8, 2100902 (2022).

[70] S. Wang, H. Chen, T. Liu, Y. Wei, G. Yao, Q. Lin, X. Han, C. Zhang, H. Huang, Retina-inspired organic photonic synapses for selective detection of SWIR light. Angew. Chem. Int. Ed. 62, e202213733 (2023). 10.1002/anie.202213733 36418239

[71] L. Xu, J. Liu, X. Guo, S. Liu, X. Lai, J. Wang, M. Yu, Z. Xie, H. Peng, X. Zou, X. Wang, R. Huang, M. He, Ultrasensitive dim-light neuromorphic vision sensing via momentum-conserved reconfigurable van der Waals heterostructure. Nat. Commun. 15, 9011 (2024). 39424814 10.1038/s41467-024-53268-4 PMC11489728

[72] R. Yang, Y. Wang, S. Li, D. Hu, Q. Chen, F. Zhuge, Z. Ye, X. Pi, J. Lu, All-optically controlled artificial synapse based on full oxides for low-power visible neural network computing. Adv. Funct. Mater. 34, 2312444 (2024).

[73] Z. Zhao, Z. Hu, M. Deng, E. Hong, P. Wang, Z. Li, X. Fang, Bias-switchable photodetection and photosynapse dual-functional devices based on 2D perovskite/organic heterojunction for imaging-to-recognition conversion. Adv. Mater. 37, e2416033 (2025). 39648569 10.1002/adma.202416033

[74] T. Liu, H. Wang, C. Sun, Z. Yuan, X. Wang, L. Wang, J. Wang, S. Wang, Q. Zhang, L. Huang, W. Wu, L. Li, X. Meng, Suppression of tin oxidation via Sn→B bonding interactions for high-resolution lead-free perovskite neuromorphic imaging sensors. Adv. Mater. 37, e2502015 (2025). 40341612 10.1002/adma.202502015

[75] Z. Cheng, T. Wang, J. Zhu, Y. He, S. Liu, M.-Y. Li, H. Lu, X. Wen, J. Lee, S. Liu, S. Mao, All-inorganic lead-free Cs_2_AgBiBr_6_/ZnO artificial retina synapse based on photoelectric synergistic dual-mechanism for neuromorphic computing. Small 21, e2411129 (2025). 39895204 10.1002/smll.202411129

[76] Y. Chen, J. Xia, Y. Qu, H. Zhang, T. Mei, X. Zhu, G. Xu, D. Li, L. Wang, Q. Liu, K. Xiao, Ephaptic coupling in ultralow-power ion-gel nanofiber artificial synapses for enhanced working memory. Adv. Mater. 37, e2419013 (2025). 40059495 10.1002/adma.202419013

[77] D. Temel, G. Kwon, M. Prabhushankar, G. AlRegib, CURE-TSR: Challenging unreal and real environments for traffic sign recognition, Zenodo (2020); 10.5281/zenodo.3903066.

[78] D. Temel, G. Kwon, M. Prabhushankar, G. AlRegib, CURE-TSR: Challenging unreal and real environments for traffic sign recognition. arXiv:1712.02463 [cs.CV] (2017).

[79] G. Kresse, J. Hafner, Ab initio molecular dynamics for liquid metals. Phys. Rev. B 47, 558–561 (1993). 10.1103/physrevb.47.558 10004490

[80] G. Kresse, J. Hafner, Ab initio molecular-dynamics simulation of the liquid-metal–amorphous-semiconductor transition in germanium. Phys. Rev. B 49, 14251–14269 (1994). 10.1103/physrevb.49.14251 10010505

[81] G. Kresse, J. Furthmüller, Efficiency of ab-initio total energy calculations for metals and semiconductors using a plane-wave basis set. Comput. Mater. Sci. 6, 15–50 (1996).

[82] G. Kresse, J. Furthmüller, Efficient iterative schemes for ab initio total-energy calculations using a plane-wave basis set. Phys. Rev. B 54, 11169–11186 (1996). 10.1103/physrevb.54.11169 9984901

[83] J. P. Perdew, M. Ernzerhof, K. Burke, Rationale for mixing exact exchange with density functional approximations. J. Chem. Phys. 105, 9982–9985 (1996).

[84] Y. Hinuma, G. Pizzi, Y. Kumagai, F. Oba, I. Tanaka, Band structure diagram paths based on crystallography. Comput. Mater. Sci. 128, 140–184 (2017).

[85] A. Togo, K. Shinohara, I. Tanaka, Spglib: A software library for crystal symmetry search. Sci. Technol. Adv. Mater. Methods 4, 2384822 (2024).

[86] V. Wang, N. Xu, J.-C. Liu, G. Tang, W.-T. Geng, VASPKIT: A user-friendly interface facilitating high-throughput computing and analysis using VASP code. Comput. Phys. Commun. 267, 108033 (2021).

[87] S. Fu, X. Huang, G. Gao, P. St. Petkov, W. Gao, J. Zhang, L. Gao, H. Zhang, M. Liu, M. Hambsch, W. Zhang, J. Zhang, K. Li, U. Kaiser, S. S. P. Parkin, S. C. B. Mannsfeld, T. Zhu, H. I. Wang, Z. Wang, R. Dong, X. Feng, M. Bonn, Unveiling high-mobility hot carriers in a two-dimensional conjugated coordination polymer. Nat. Mater. 24, 1457–1464 (2025). 40360871 10.1038/s41563-025-02246-2 PMC12404993

[88] J. Song, Y. Zhou, N. P. Padture, B. D. Huey, Anomalous 3D nanoscale photoconduction in hybrid perovskite semiconductors revealed by tomographic atomic force microscopy. Nat. Commun. 11, 3308 (2020). 32620841 10.1038/s41467-020-17012-y PMC7335063

[89] L. Bernstein, A. Sludds, C. Panuski, S. Trajtenberg-Mills, R. Hamerly, D. Englund, Single-shot optical neural network. Sci. Adv. 9, eadg7904 (2023). 37343096 10.1126/sciadv.adg7904 PMC10284542

[90] V. Tripathi, B. Murmann, An 8-bit 450-MS/s single-bit/cycle SAR ADC in 65-nm CMOS, in 2013 Proceedings of the ESSCIRC (ESSCIRC), Bucharest, Romania, 16–20 September 2013 (IEEE, 2013), pp. 117–120.

[91] J. Song, J. Meng, C. Lu, T. Wang, C. Wan, H. Zhu, Q. Sun, D. W. Zhang, L. Chen, Self-powered optoelectronic synaptic device for both static and dynamic reservoir computing. Nano Energy 134, 110574 (2025).

[92] Y.-C. Yao, C.-J. Lee, Y.-J. Chen, J.-Z. Feng, H. Oh, C.-S. Lue, J.-K. Sheu, Y.-J. Lee, All-inorganic perovskite quantum-dot optical neuromorphic synapses for near-sensor colored image recognition. Adv. Sci. 12, e2409933 (2025). 10.1002/advs.202409933 PMC11791932 39680661

[93] W. Liu, J. Wang, J. Guo, L. Wang, Z. Gu, H. Wang, H. Fang, Efficient carbon-based optoelectronic synapses for dynamic visual recognition. Adv. Sci. 12, e2414319 (2025). 10.1002/advs.202414319 PMC11923932 39840530

[94] J. Chen, Y. Huang, H. Wen, Y. Wang, H. Li, X. Zheng, X. Wang, Z. Ma, T. Wang, S. Yan, K. Wang, L. Zhao, High sensitivity optoelectronic artificial synapse based on GaN porous nanocone array for neuromorphic computing. Adv. Opt. Mater. 13, 01078 (2025).

[95] Y. Yuan, Z. Xu, D. Zhang, C. Huang, W. Xu, Air-stable hydrogen-substituted graphdiyne/2D halide perovskite heterojunction for self-powered neuromorphic vision. Adv. Funct. Mater. 35, 2504542 (2025).

[96] W. Qiu, Y. Huang, L.-A. Kong, Y. Chen, W. Liu, Z. Wang, J. Sun, Q. Wan, J. H. Cho, J. Yang, Y. Gao, Optoelectronic In-Ga-Zn-O memtransistors for artificial vision system. Adv. Funct. Mater. 30, 2002325 (2020).

[97] C. Qian, S. Oh, Y. Choi, J.-H. Kim, J. Sun, H. Huang, J. Yang, Y. Gao, J.-H. Park, J. H. Cho, Solar-stimulated optoelectronic synapse based on organic heterojunction with linearly potentiated synaptic weight for neuromorphic computing. Nano Energy 66, 104095 (2019).

[98] J. Zhang, P. Guo, Z. Guo, L. Li, T. Sun, D. Liu, L. Tian, G. Zu, L. Xiong, J. Zhang, J. Huang, Retina-inspired artificial synapses with ultraviolet to near-infrared broadband responses for energy-efficient neuromorphic visual systems. Adv. Funct. Mater. 33, 2302885 (2023).

[99] J. Zhang, Z. Guo, T. Sun, P. Guo, X. Liu, H. Gao, S. Dai, L. Xiong, J. Huang, Energy-efficient organic photoelectric synaptic transistors with environment-friendly CuInSe_2_ quantum dots for broadband neuromorphic computing. SmartMat 5, e1246 (2024).

[100] S. Das, V. Pal, S. Mukherjee, S. Das, C. S. Tiwary, S. K. Ray, Multi-wavelength optoelectronic synaptic transistors based on transition metal telluride-sulfide heterostructures. Adv. Opt. Mater. 12, 2400037 (2024).

[101] J.-L. Chen, T.-C. Chiang, P.-T. Liu, All-metal-oxide heterojunction optoelectronic synapses with multilevel memory for artificial visual perception applications. Small 21, e2502271 (2025). 40317666 10.1002/smll.202502271 PMC12199123

[102] K. Chen, Q. Dai, Y. Tian, W. Chen, C. Ai, Y. Cao, X. Yuan, Q. Wang, Y. Shi, Y. Li, Ultra-low-power vertical organic synaptic phototransistors for neuromorphic vision preprocessing. Adv. Funct. Mater. 35, 2508673 (2025).

[103] Y. Rong, D. Yu, X. Zhang, T. Wang, J. Wang, Y. Li, T. Zhao, R. He, Y. Gao, C. Huang, S. Xiao, J. Qin, S. Bai, H. Zhu, A. Liu, Y. Chen, Q. Song, Perovskite thin-film transistors for ultra-low-voltage neuromorphic visions. Adv. Sci. 11, e2410015 (2024). 10.1002/advs.202410015 PMC11672285 39501928

[104] J. Han, H. Mao, Y. Lin, K. Peng, L. Meng, Y. Ding, X. Shan, Y. Tao, J. Shi, Z. Wang, H. Zhu, X. Zhao, H. Xu, Y. Liu, Near-infrared optoelectronic memristor with all-optical modulation for microscopic biological motion recognition. Adv. Mater. 37, e10185 (2025). 40874436 10.1002/adma.202510185

[105] J. Han, M. He, M. Yang, Q. Han, F. Wang, F. Zhong, M. Xu, Q. Li, H. Zhu, C. Shan, W. Hu, X. Chen, X. Wang, J. Gou, Z. Wu, J. Wang, Light-modulated vertical heterojunction phototransistors with distinct logical photocurrents. Light Sci. Appl. 9, 167 (2020). 33042530 10.1038/s41377-020-00406-4 PMC7509774

[106] Y.-X. Hou, Y. Li, Z.-C. Zhang, J.-Q. Li, D.-H. Qi, X.-D. Chen, J.-J. Wang, B.-W. Yao, M.-X. Yu, T.-B. Lu, J. Zhang, Large-scale and flexible optical synapses for neuromorphic computing and integrated visible information sensing memory processing. ACS Nano 15, 1497–1508 (2021). 33372769 10.1021/acsnano.0c08921

[107] T. Ahmed, M. Tahir, M. X. Low, Y. Ren, S. A. Tawfik, E. L. H. Mayes, S. Kuriakose, S. Nawaz, M. J. S. Spencer, H. Chen, M. Bhaskaran, S. Sriram, S. Walia, Fully light-controlled memory and neuromorphic computation in layered black phosphorus. Adv. Mater. 33, e2004207 (2021). 33205523 10.1002/adma.202004207

[108] Y.-C. Mi, C.-H. Yang, L.-C. Shih, J.-S. Chen, All-optical-controlled excitatory and inhibitory synaptic signaling through bipolar photoresponse of an oxide-based phototransistor. Adv. Opt. Mater. 11, 2300089 (2023).

[109] S. Ge, F. Huang, J. He, Z. Xu, Z. Sun, X. Han, C. Wang, L.-B. Huang, C. Pan, Bidirectional photoresponse in perovskite-ZnO heterostructure for fully optical-controlled artificial synapse. Adv. Opt. Mater. 10, 2200409 (2022).

[110] L. Hu, J. Yang, J. Wang, P. Cheng, L. O. Chua, F. Zhuge, All-optically controlled memristor for optoelectronic neuromorphic computing. Adv. Funct. Mater. 31, 2005582 (2021).

