# Supplementary material for: Light-tunable charge dynamics enable ultralow-power bidirectional lead-free perovskite synapses for intelligent vision
Source: Sci Adv. 2026 Sep 2;12(36):eaeh3751. doi: 10.1126/sciadv.aeh3751 (PMC13537266; doi:10.1126/sciadv.aeh3751)
Supplement: Supplementary file 1 [file sciadv.aeh3751_sm.pdf]

Science Advances
AAAS

Supplementary Materials for

Light-tunable charge dynamics enable ultralow-power bidirectional lead-free perovskite synapses for intelligent vision

Tingting Mei *et al.*

Corresponding author: Long Hu, long.hu@unsw.edu.au; Miaoqiang Lyu, m.lyu@uq.edu.au; Chun-Ho Lin, chun-ho.lin@unsw.edu.au; Dewei Chu, d.chu@unsw.edu.au

Sci. Adv. **12**, eaeh3751 (2026)
DOI: 10.1126/sciadv.aeh3751

This PDF file includes:

Supplementary Text 1 to 4
Figs. S1 to S48
Tables S1 to S4
References

Supplementary Note

1 DFT Calculation

The Density Functional Theory (DFT) calculation was conducted by the Vienna Ab initio Simulation Package (VASP)(*79-82*). The generalised gradient approximation (GGA) with the Perdew-Burke-Ernzerhof (PBE)(*83*) function as the exchange-correlation energy function was used. The conjugate gradient scheme optimises the atom coordinates until the force is less than 0.01 eV Å-1. The cutoff energy is 500 eV and the D3 correction was applied for all the layer structure calculations to consider the Van der Waals effect.

The bulk structure was relaxed first according to the refined XRD lattice parameters (a=8.6592 Å, b=8.606 Å, c=16.3779 Å, α=94.7521°, β=99.9586°, γ=89.9649°) with the 4 × 4 × 2 of k-points for Monkhorst-Pack BZ calculations to achieve energy convergence.

The band structure calculation was based on the above refined lattice parameters. With the aP Bravais Lattice as shown in **Figure S1**.

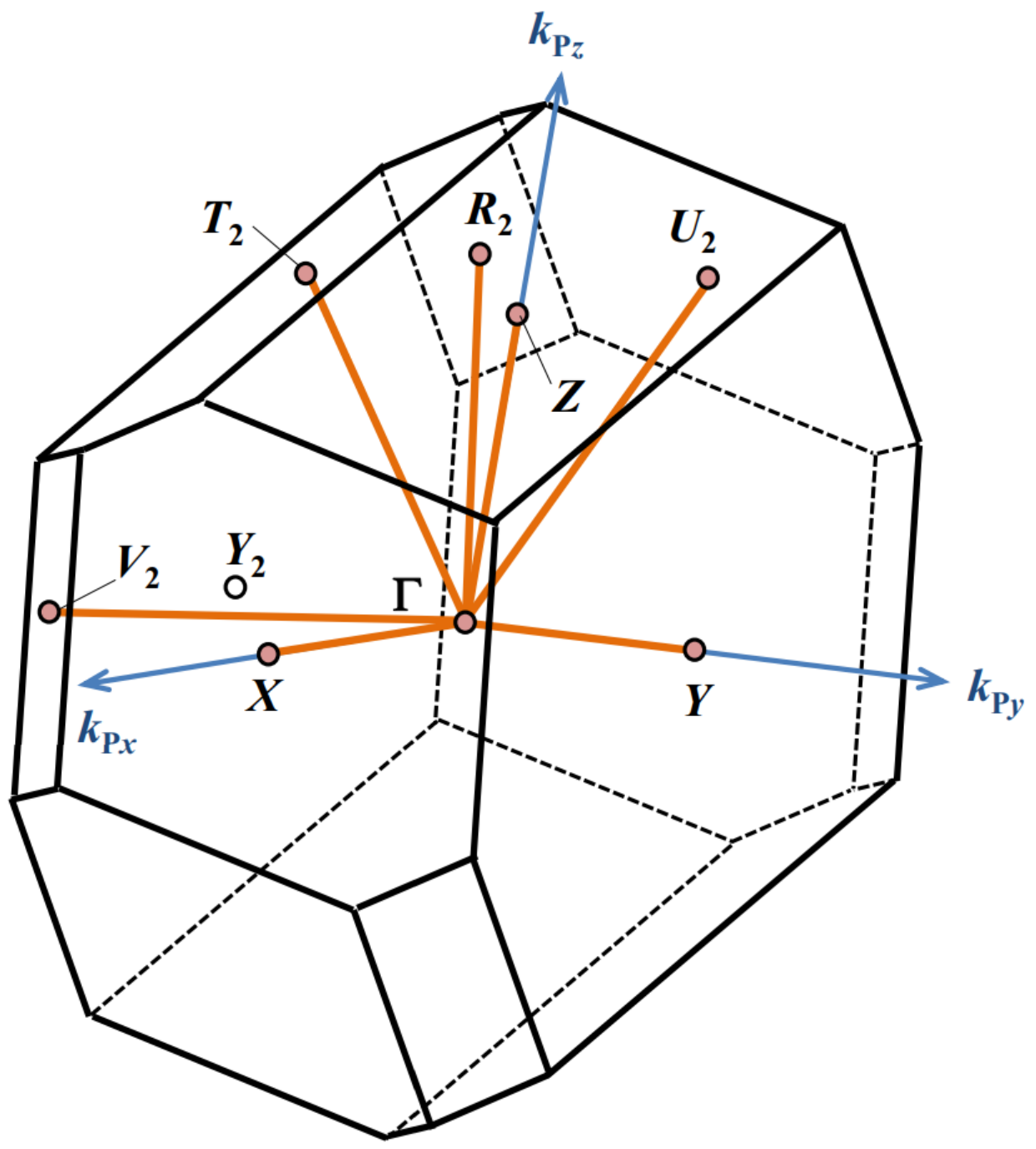

Figure S1. The BZ, special BZ points, and recommended band path for aP when the interaxial angles of the reciprocal "reduced" cell are all acute. Filled red circles indicate one representative of each special k-vector point in the highest symmetry reciprocal space group type. The bold lines indicate segments of the recommended band path, which is G-Z | Y-G-Y_2 | X-G-V_2 | U_2-G-T_2-R_2(*84*). The high symmetry points were generated via the VASPKIT (Edition 1.3.5)(*85,86*)

We have considered all the possible point defects, including three vacancies (V_{PEA}, V_{Sn}, V_I), two interstitials (i_I, i_{Sn}), one cation substitution (Sn_{PEA}), and three anti-site substitutions (I_{PEA}, I_{Sn}, Sn_I) with the 2 × 2 × 1 supercell and 2 × 2 × 2 k-points for Monkhorst-Pack BZ.

2 Quantitative calculations of effective carrier population

The effective carrier population associated with the transient excitatory or inhibitory postsynaptic current response was estimated from the photoinduced conductance modulation. Under a fixed drain bias, the transient current change was first obtained by subtracting the dark current from the light-induced current:

$$\Delta I(t) = I_{\text{light}}(t) - I_{\text{dark}}.$$

The corresponding transient conductance modulation is therefore:

$$\Delta G(t) = \frac{\Delta I(t)}{V_{\text{DS}}},$$

where V_{DS} is the drain-source bias used during the pulsed optical measurement. For a lateral transistor channel with channel length L, width W, and effective conducting thickness t, the conductance change can be converted into a conductivity change:

$$\Delta\sigma(t) = \Delta G(t)\frac{L}{Wt}.$$

This conversion follows the standard relationship between conductance and conductivity for a rectangular conduction channel:

$$G = \sigma\frac{Wt}{L}.$$

The photoinduced conductivity is related to the carrier population and carrier mobility through:

$$\Delta\sigma(t) = q\mu\Delta p_{\text{ph}}(t),$$

where q is the elementary charge, μ is the carrier mobility, and Δp_{ph} is the conductance-equivalent carrier population for p-type semiconductor. This relationship is widely used in photoconductivity analysis, where the transient photoconductivity is expressed as the product of photogenerated carrier density, elementary charge, and carrier mobility(*87, 88*). Combining the previous equations gives:

$$\Delta p_{\text{ph}}(t) = \frac{[I_{\text{light}}(t) - I_{\text{dark}}]L}{q\mu_p V_{\text{DS}} Wt}.$$

The hole mobility μ_p was extracted from the dark transfer characteristics of the transistor measured in the linear regime.

3 Attention-weight enhanced traffic sign recognition.

(a) Dataset

The dataset used for traffic sign recognition task is Challenging Unreal and Real Environments for Traffic Sign Recognition (CURE-TSR) obtained online, which is a large-scale benchmark designed to evaluate traffic sign recognition algorithms under diverse and controlled challenging conditions. It comprises over 2 million annotated images of 14 types of traffic signs (**Fig. S39**), sourced from both real-world footage (captured in Belgium) and simulated environments created using Unreal Engine 4(*77,78*). In this paper, only real-world images are used for training.

(b) Attention weight modulation

To incorporate the intensity-dependent response of the optoelectronic synaptic device into image-level attention, we constructed attention maps based on the device's excitatory postsynaptic current (EPSC) responses under 6 light intensity levels (**Fig. S38**).

For each original traffic sign image *I*, an attention-enhanced image I' was generated by element-wise multiplication with the corresponding attention weight w_i derived from the light intensity level:

$$I_i'(x,y) = (w_i + 1) \times I(x,y)$$

Where (x,y) denotes the pixel coordinates, and w_i is the normalized EPSC response at light level i.

(c) Convolutional neural network (CNN) training

To evaluate the effectiveness of the synaptic attention mechanism in traffic sign recognition, we implemented a lightweight convolutional neural network based on a simplified LeNet architecture. The input image is processed by two convolutional poolings to extract hierarchical features and reduce spatial dimensions. Then the resulting feature maps are flattened and passed through a fully connected layer with 128 neurons and ReLU activation. The final layer is a fully connected output layer that maps to the number of traffic sign classes via a softmax operation during training.

The model is trained using a cross-entropy loss function. Optimization is performed using the Adam algorithm and a learning rate scheduler is applied to facilitate stable convergence.

(d) Challenge testing

To evaluate the robustness of CNN model, three environmental challenges: dark, shadow, and rain, are simulated on the original real-world images at five different strength levels as testing sets. To demonstrate the feasibility under the assistance of the optoelectronic synaptic device, attention weight modulation was processed on environmental challenges as attention-processed testing set.

(e) System-level power consumption calculation

The total energy of the present hybrid hardware-software demonstration can be decomposed as:

$$E_{system} = E_{device-attention} + E_{data\ conversion} + E_{CNN}$$

Here, the experimentally measured device contribution can be estimated as:

$$E_{\text{device}-\text{attention}} = N_{\text{pixel}} N_{\text{pulse}} E_{\text{event}},$$

where N_{pixel} is the number of processed pixels, N_{pulse} is the number of optical weighting events per pixel, and E_{event} is the measured single-event energy. So, for a 128 × 128 attention layer with one optical event per pixel, the projected device-array attention energy is:

$$E_{\text{device}-\text{attention}} = 128 \times 128 \times 1 \text{ fJ} = 16.4 \text{ pJ/frame}$$

In this proof-of-concept demo, data conversion was implemented off-chip. So, we provide an idealized component-level estimate to clarify the additional energy required between the device sensing and the digital CNN. The data conversion is defined as the sum of the transimpedance amplifier (TIA) and Analog-to-Digital Converter (ADC):

$$E_{data\ conversion} = E_{TIA} + E_{ADC}$$

According to the system-energy model reported for a single-shot optical neural network(*89, 90*), a near-term CMOS-compatible implementation includes TIA and ADC contributions, with $E_{\mathrm{TIA}} \approx 1$ pJ per analog readout operation and $E_{\mathrm{ADC}} \approx 2$ pJ per ADC conversion. Therefore, the projected readout and digitization energy was estimated as:

$$E_{\mathrm{TIA}} = N_{\mathrm{sample}} E_{\mathrm{TIA/sample}}$$

$$E_{\mathrm{ADC}} = N_{\mathrm{sample}} E_{\mathrm{ADC/sample}}$$

For a 128×128 single-channel attention map, $N_{\mathrm{sample}} = 16384$. Therefore:

$$E_{\mathrm{TIA}} = 16384 \times 1\ \mathrm{pJ} = 16.4\ \mathrm{nJ/frame},$$
$$E_{\mathrm{ADC}} = 16384 \times 2\ \mathrm{pJ} = 32.8\ \mathrm{nJ/frame}.$$

Thus, the idealized readout and digitization overhead is:

$$E_{\mathrm{TIA+ADC}} = 49.2\ \mathrm{nJ/frame}.$$

To evaluate the energy consumption of CNN, we need to first calculate the number of multiply-and-accumulate (MACs) in each convolutional layer as:

$$N_{\mathrm{MAC,conv}} = H_{\mathrm{out}} W_{\mathrm{out}} C_{\mathrm{out}} K_h K_w C_{\mathrm{in}},$$

where H_{out}, W_{out}, and C_{out} are the height, width, and channel number of the output feature map, K_{h} and K_{w} are the convolutional kernel sizes, and C_{in} is the number of input channels. The number of MACs in each fully connected layer was calculated as:

$$N_{\mathrm{MAC,FC}} = N_{\mathrm{in}} N_{\mathrm{out}}.$$

The detailed MAC count is summarized in the following Table.

Table S1. Parameter summary of CNN model

**Layer**	**Output size**	**MACs/frame**	**Fraction of total MACs**	**FP32 energy/frame**
Conv1	**124×124×16**	18,451,200	28.41%	84.88 μJ
Conv2	**58×58×32**	43,059,200	66.29%	198.07 μJ
FC1	**26912/128**	3,444,736	5.30%	15.85 μJ
FC2	**128/14**	1,792	0.003%	0.008 μJ
**Total**	**-**	**64,956,928**	**100%**	**298.80 μJ**

The total number of MACs required for one forward inference is therefore:

$$N_{\mathrm{MAC,total}} = 6.50 \times 10^7\ \mathrm{MACs/frame}.$$

Following the commonly used 45 nm CMOS operation-energy benchmark, a 32-bit floating-point multiplication consumes approximately 3.7 pJ, and a 32-bit floating-point addition

consumes approximately 0.9 pJ. Thus, the energy of one FP32 MAC operation was approximated as:

$$E_{\text{MAC,FP32}} = E_{\text{mult,FP32}} + E_{\text{add,FP32}} = 3.7 \text{ pJ} + 0.9 \text{ pJ} = 4.6 \text{ pJ/MAC}.$$

The FP32 CNN compute energy per frame was then estimated as:

$$\begin{aligned} E_{\text{CNN}} &= N_{\text{MAC,total}} E_{\text{MAC,FP32}}, \\ &= 6.50 \times 10^{7} \times 4.6 \text{ pJ} \approx 2.99 \times 10^{-4} \text{ J/frame}, \\ &\approx 298.8\ \mu\text{J/frame}. \end{aligned}$$

Notably, this value represents only the arithmetic compute energy of the CNN forward inference; it is interpreted as a compute-only lower-bound estimate of the digital CNN contribution. Therefore, the total energy cost is estimated as:

$$\begin{aligned} E_{system} &= E_{device-attention} + E_{data\ conversion} + E_{CNN} \\ &= 16.4 \text{ pJ/frame} + 49.2 \text{ nJ/frame} + 298.8\ \mu\text{J/frame} \\ &\approx 298.85\ \mu\text{J/frame} \end{aligned}$$

Table S2. Energy consumption calculation results

Term	Device-attention operation	Data conversion	CNN compute	Total
Estimated energy consumption (per frame)	16.4 pJ	49.2 nJ	298.8 μJ	298.85 μJ

The estimated hybrid-pipeline energy is ~298.85 μJ per frame, with the digital CNN contributing > 99% of the total. The role of the synaptic device is to provide a pJ-level analog front-end attention function with negligible energy overhead, enabling a lightweight classifier to maintain high recognition robustness under challenging visual conditions. Under the same CNN architecture and training setting, the conventional system without device-assisted attention showed much lower accuracies under the highest dark, shadow, and rainy challenges, reaching only 45.6%, 64.5%, and 64.3%, respectively. In contrast, the perovskite/C_{60} optoelectronic synaptic system with hardware-assisted attention weighting achieved 91.2%, 99.2%, and 88.2%, corresponding to markedly improved performance by 45.6, 34.7, and 23.9 percentage points. This indicates that the synaptic device reduces the representational burden of the digital backend by performing task-relevant feature enhancement at the optical front end. Without such hardware-assisted preprocessing, comparable accuracy using a conventional system would likely require a substantially larger model, more training data, additional software enhancement or extended training, thereby shifting the energy burden to the digital backend.

4 Reservoir Computing (RC) system for MNIST digit recognition and synaptic pruning.

The physical reservoir computing framework implemented in this work consists of three sequential stages: (a) input encoding, (b) dynamic synaptic modulation via the optoelectronic reservoir, and (c) output readout and classification.

(a) Input encoding.

To demonstrate the basic encoding mechanism, we first employed a four-bit binary input scheme in which each bit ("0" or "1") was translated into the presence or absence of a short-wavelength optical pulse, respectively (**Figure S46**). Each pulse had a fixed width and intensity, representing a time-domain stimulus analogous to sensory spiking activity. Building upon this demonstration, we encoded image data by randomly selecting 100 handwritten digits from the MNIST dataset. Each 28 × 28 grayscale image was spatially compressed by grouping every four pixels into a single unit, resulting in a 14 × 14 feature map (**Figure S47)**. This map was then flattened into a one-dimensional sequence and converted into a temporal stream of optical pulses. Therefore, each image was converted into a structured temporal optical-pulse sequence with 196 possible pulse positions (**Figure S48)**. In this encoding, pixel intensities above a fixed threshold were translated into pulse "1" (one light pulse stimuli), while others were treated as "0" (no light pulse), generating a structured temporal signal that mimics retinal-to-cortical spiking input.

(b) Dynamic synaptic modulation via the optoelectronic reservoir

The resulting optical pulse sequences (196 nodes) were delivered to the optoelectronic synaptic device, to simulate dynamic signal propagation, and the drain current was recorded using a Keithley 4200 semiconductor parameter analyser in sampling mode with quiet-speed acquisition. For each measurement, a baseline segment was first recorded before optical stimulation to determine the initial current level. The subsequent current response during the 196-pulse input window was used for reservoir-state extraction. The effective optical-response segment contained 485 valid current data points for each image. These acquisition-defined temporal samples were used as the reservoir-state components:

$$\mathbf{x}_i = [\Delta I_i(t_1), \Delta I_i(t_2), \ldots, \Delta I_i(t_{485})] \in \mathbb{R}^{485},$$

where i denotes the i-th input image, t_j is the j-th sampling point recorded by the semiconductor parameter analyser, and $\Delta I_i(t_j)$is the baseline-corrected current response. Thus, although the physical output of the single transistor is a one-dimensional current trace, its time-discretized evolution forms a 485-dimensional temporal reservoir-state vector.

The use of these temporal states is enabled by the nonlinear short-term memory and temporal integration of the optoelectronic synaptic device. Owing to fading memory, each sampled current value is influenced not only by the instantaneous optical stimulus but also by the preceding pulse history. Therefore, the 485-point temporal trajectory encodes the cumulative spatiotemporal information of the 196-pulse input sequence and serves as the physical reservoir output for classification.

To further enhance information separability and reduce redundancy, a global suppression step was performed by illuminating the device with long-wavelength light. This induced inhibitory synaptic plasticity, effectively removing low-weight connections across the reservoir, analogous to biological synaptic pruning.

(c) Output readout and classification

Before classification, each experimentally obtained reservoir-state vector was normalized by min-max scaling to the range of 0-20:

$$\mathbf{x}_i' = 20 \times \frac{\mathbf{x}_i - \min(\mathbf{x}_i)}{\max(\mathbf{x}_i) - \min(\mathbf{x}_i)}.$$

To improve generalization and emulate experimental input variability, Gaussian noise sampled from $N(0,0.5)$was added to the normalized response vectors for data augmentation. This

procedure generated 5,000 augmented samples, with 500 examples for each digit class from 0 to 9.

The normalized temporal reservoir states were then assembled into a feature matrix:

$$\mathrm{X} \in \mathbb{R}^{N\times 485},$$

where N is the number of training samples and 485 is the dimensionality of each acquisition-defined temporal state vector. The digit labels were represented by a one-hot target matrix:

$$\mathrm{Y} \in \mathbb{R}^{N\times 10}.$$

A ridge-regression readout was trained in Python to map the temporal reservoir states to the corresponding digit labels:

$$\mathrm{W}_{out} = (\mathrm{X}^T\mathrm{X} + \lambda\mathrm{I})^{-1}\mathrm{X}^T\mathrm{Y},$$

where λ is the regularization parameter. During validation, the predicted class was determined from the maximum output score:

$$\hat{y} = \arg\max(\mathrm{x}'\mathrm{W}_{out}).$$

The dataset was divided into 80% training data and 20% validation data. Because only the linear readout weights were trained while the physical reservoir remained fixed, this protocol follows the standard reservoir-computing framework. The result demonstrates that the optoelectronic synaptic transistor can transform a time-serialized optical input sequence into a high-dimensional temporal state vector through its intrinsic nonlinear and fading-memory dynamics, enabling low-complexity digit classification with ridge-regression readout.

Table S3. Comparison of reported optoelectronic artificial synapses in the past 5 years.

Active layer	Operation mode		MAX PPF Index	MAX PPD Index	Neuromorphic applications	Model	Device array
	LTP	LTD					
$Ca_2Nb_3O_{10}$/OBHJ*(73)*	300 nm	-	308%	-	Image recognition	ANN	28 × 28
NiO/IGZO*(91)*	470 nm	Electric	112%	-	Image recognition and spoken signal recognition	Reservoir computing	no
$(BA)_2PbI_4$*(21)*	410 nm	-	126%	-	Image recognition and facial recognition	ANN	no
$Cs_{0.05}MA_{0.15}FA_{0.8}PbI_{0.85}Br_{0.15}$ *(23)*	405/550/650nm	Electric	~128%	-	Image recognition	ANN	no
$CsPbBr_3$ QDs *(92)*	325/420/525nm	-	186%	-	Image recognition	ANN	no
$FASnI_3$-QR *(22)*	785nm	-	~158%	-	Image recognition and motion perception	ANN	12 × 12
C_{60}@Go *(93)*	395/532/635 nm	-	157%		Dynamic Vision Recognition	ANN	5 × 5
BA- $(4\text{-Cl-PEA})_2SnI_4$ *(74)*	520 nm	-	240%	-	Image recognition	ANN	32 × 32
GaN *(94)*	370 nm	Electric	~157%	-	Image recognition	ANN	no
HsGDY/$(BA)_2PbI_4$ *(95)*	405/520 nm	Electric	~136%	-	Dynamic Vision Recognition	no	5 × 5
$Cs_2AgBiBr_6$/ZnO *(74)*	410 nm	Electric	~108%		Image recognition	ANN	no
$CsPbBr_3$ QDs *(59)*	365/450/520/660 nm	Electric	130%	-	no	no	no
Si-NM/$MAPbI_3$ *(65)*	532 nm	-	168%	-	no	no	no
$CsBi_3I_{10}$/SWCNTs *(66)*	450 nm	-	~152%	-	no	no	no
InGaZnO *(96)*	405 nm	Electric	177%	-	Image recognition	ANN	no
C_3N_4/PMMA/ Pentacene *(64)*	365/460/520/625nm	-	120%		no	no	no
PP/SnOx/IGZO *(60)*	400/450/530/600/650nm	Electric	~160%	-	no	no	no
CuPc/p-6P *(97)*	365/450/655 nm	Electric	-	-	Image recognition	ANN	no
PQDs *(18)*	440 nm	Electric	163%	99%	Facial recognition	ANN	no
Si NCs *(63)*	1342 nm	Electric	~150%	~190%	Image recognition	ANN	no
PbS QDs *(98)*	365/550/850nm	Electric	~129%	-	no	no	6 × 6
$CuInSe_2$ QDs *(99)*	365/500/850nm	Electric	~130%	-	Image recognition	ANN	no
DPPDTT/MI *(25)*	400 nm	Electric	187%	-	Image recognition	ANN	no
$CoTe_2$/ZnO/WS_2 *(100)*	375/570/800nm	-	~160%	-	no	no	no

WO_3/InWZnO *(101)*	460/525/650nm	Electric	176%	-	Image recognition	ANN	no
Rubrene/graphene *(102)*	365 nm	-	~132%		Image recognition	ANN	no
$(POE)_2SnI_4$ *(24)*	460/525/625nm	Electric	-	-	no	no	no
PEA_2SnI_4 *(103)*	405 nm	-	148%		Image recognition	ANN	no
PEA_2SnI_4/PEIE/C_{60} *(35)*	530 nm	Electric	~138%	-	Image recognition	ANN	no
**This work PEA_2SnI_4/C_{60}**	**470/554/625nm**	**740/850 nm**	**148%**	**265%**	**Image recognition, edge detection, motion perception**	**ANN and Reservoir computing**	**7 × 7**

Table S4. Comparison of reported all-optical optoelectronic artificial synapses in the past 5 years.

active layer	Structure	excitation	excitation mechanism	inhibition	inhibition mechanism	Energy consumption
ZnO-UCNPs *(104)*	hybrid	350 nm	accumulation	980 nm	photothermal conversion effect	
Bi_2O_2Se/graphene *(28)*	hybrid	635 nm	accumulation	365 nm	Trap-assisted recombination	100 nJ
Si NCs/P_3HT *(29)*	hybrid	532 nm	photogating	375 nm	Trap-assisted recombination	-
$PtSe_{2-x}$ *(26)*	hybrid	980 nm	accumulation	405 nm	Mobility suppression	-
$Cs_xFA_yMA_{1-x-y}Pb(IzBr_{1-z})_3$ *(30)*	hybrid	630 nm	accumulation	630 nm	Trap-assisted recombination	-
Graphene/C_{60}/Pentacene *(105)*	Double junction	808 nm, 980 nm	injection	450 nm, 650 nm	Interface electron transfer	-
Pyr-GDY/Gr/PbS-QD *(106)*	Double junction	980 nm	photogating	450 nm	Trap-assisted recombination	14 nJ
BP/P_xO_y *(107)*	Heterojunction	280 nm	accumulation	365 nm	Trap-assisted recombination	1 nJ
IGZO/ZrO_x *(108)*	Heterojunction	405 nm	accumulation	890 nm	Mobility suppression	22.9 nJ
ZnAlSnO/SnO *(72)*	Heterojunction	635 nm	accumulation	532 nm	Interface electron transfer	0.75 pJ
$MAPbBr_3$/ZnO *(109)*	Heterojunction	365 nm	accumulation	520 nm	Interface electron transfer	0.1 nJ
OD-IGZO/OR-IGZO *(110)*	Heterojunction	420 nm	accumulation	800 nm	Trap-assisted recombination	170 pJ
**This work PEA_2SnI_4/C_{60}**	**Heterojunction**	**425 nm, 554 nm, 632 nm**	**accumulation**	**740nm, 850nm**	**Sub-bandgap absorption-driven charge-transfer**	**0.99 fJ**

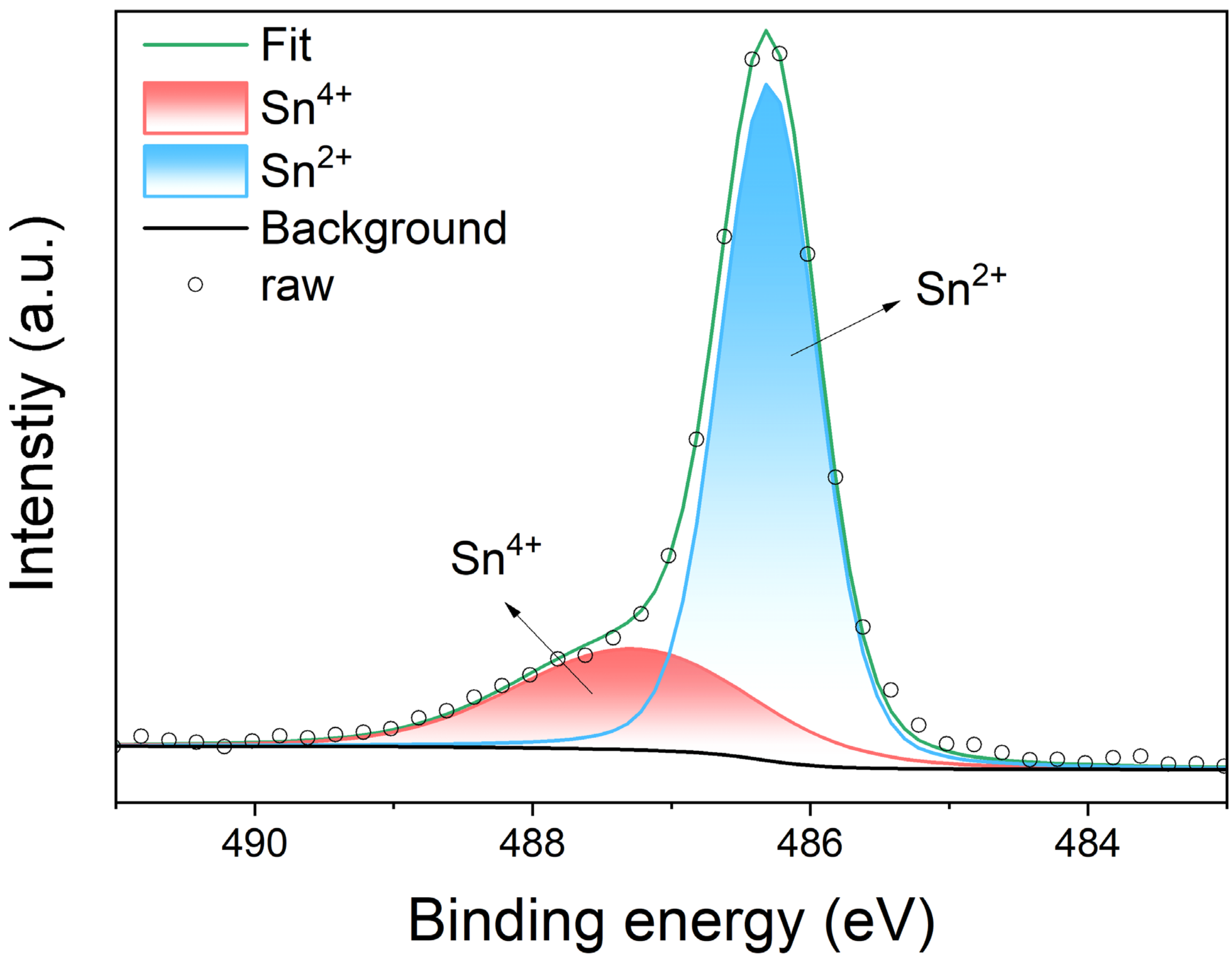

Figure S2. XPS of PEA_2SnI_4 material.

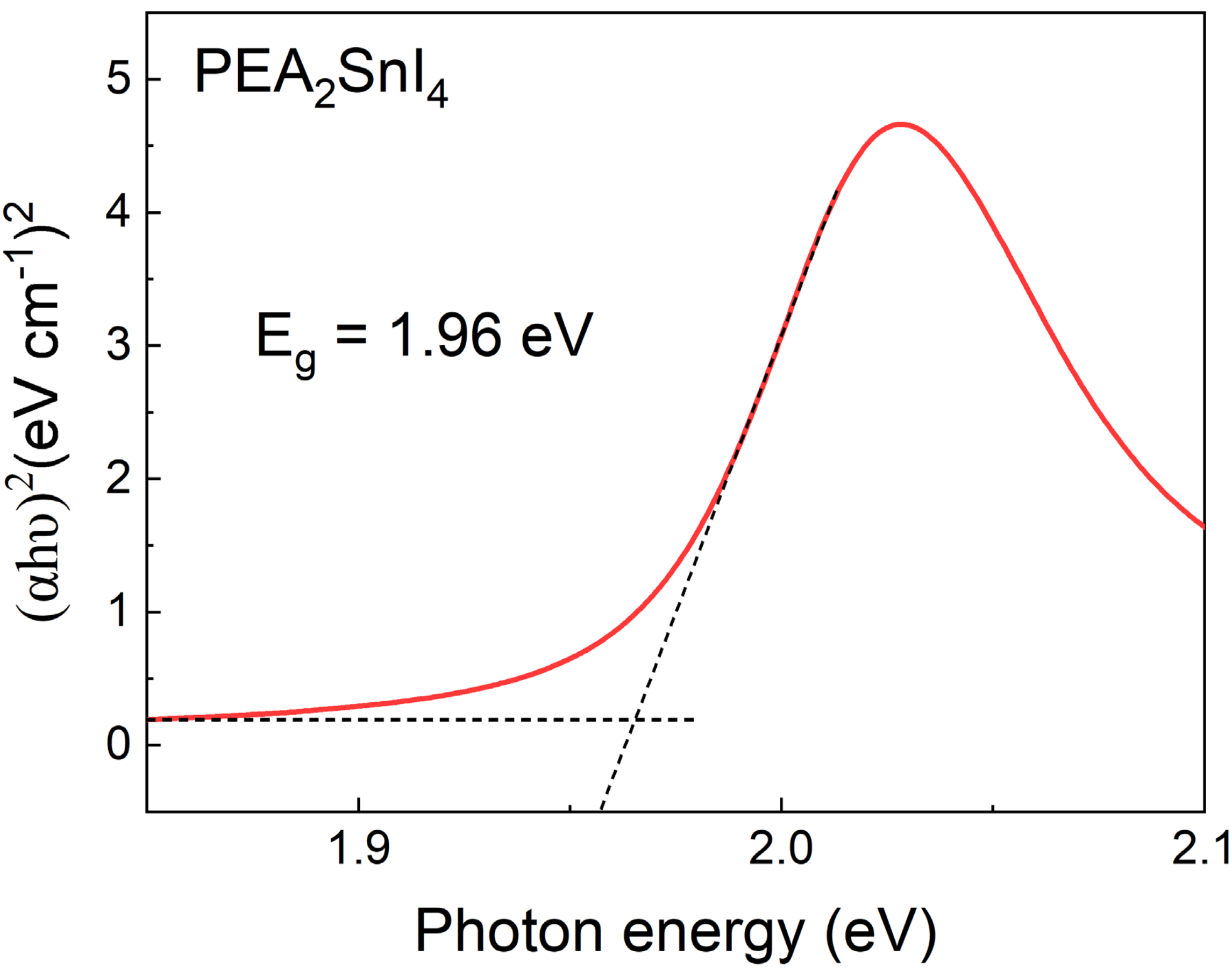

Figure S3. Tauc plot of the PEA_2SnI_4 thin film extracted from UV-vis spectrum. The band gap energy is 1.96 eV.

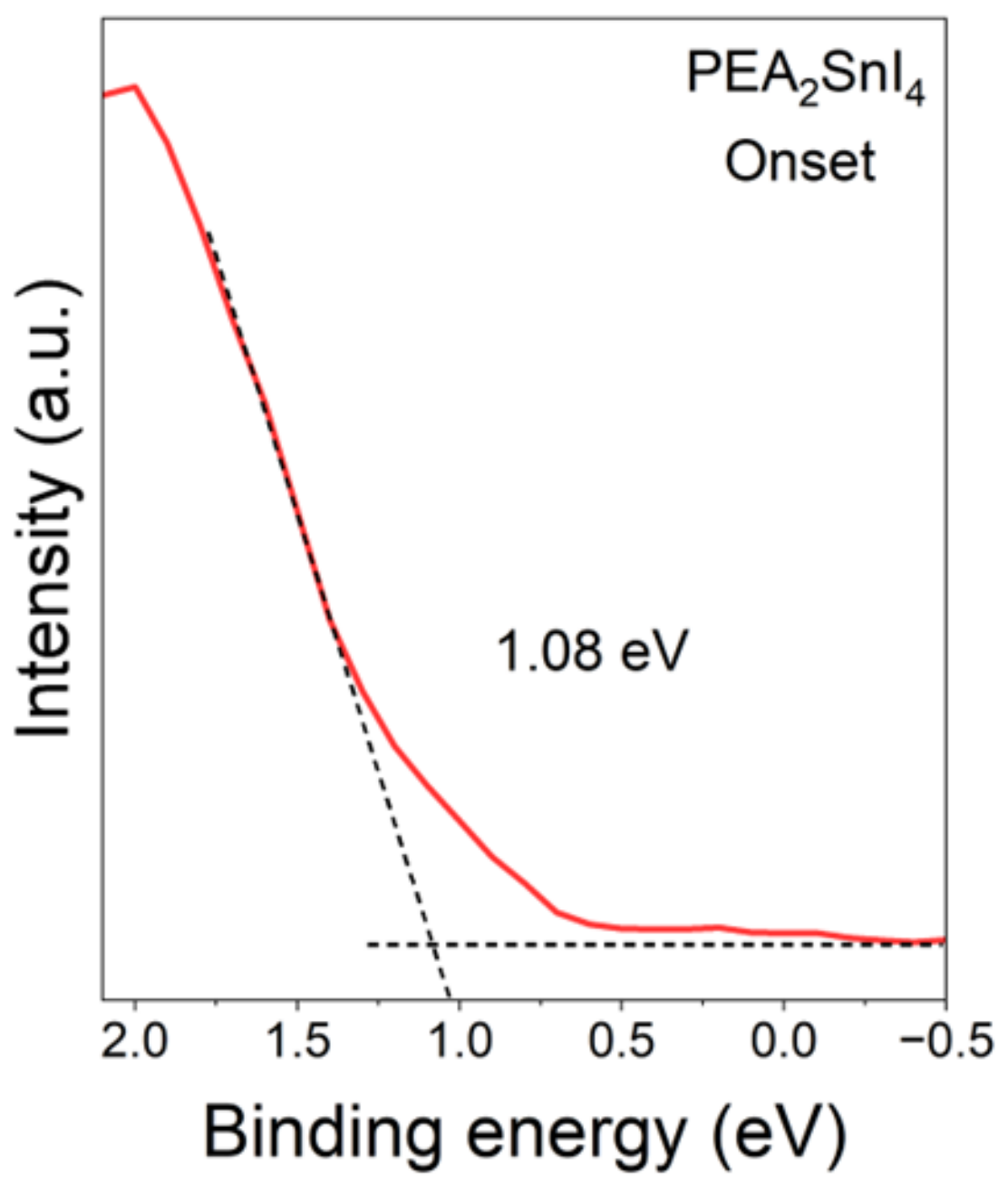

Figure S4. UPS spectra of PEA_2SnI_4 thin film in the cutoff and the onset.

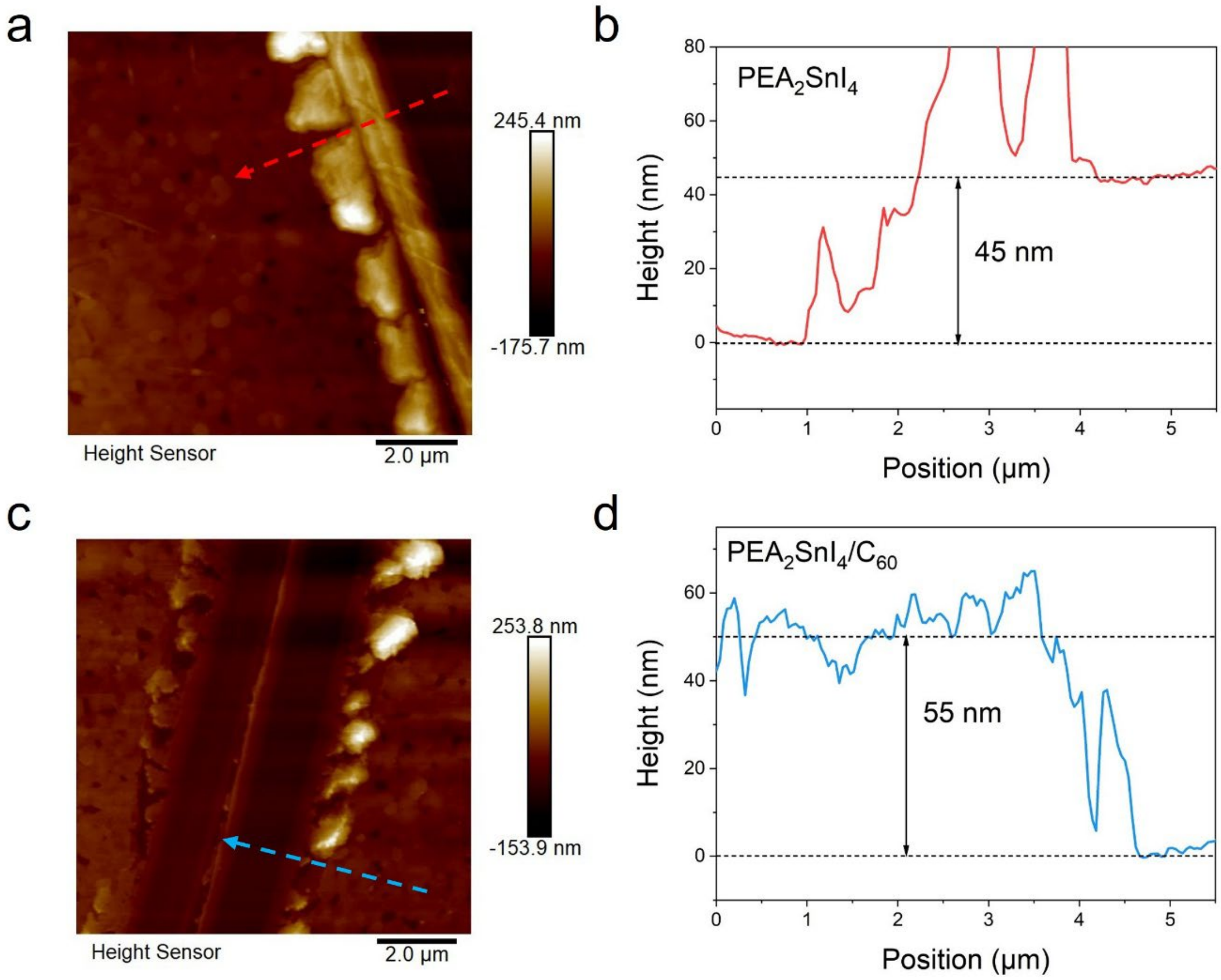

Figure S5. Thickness measurement of PEA_2SnI_4 and PEA_2SnI_4/C_{60} thin films. (a) AFM image of PEA_2SnI_4 thin film. (b) The corresponding depth profile, the thickness of PEA_2SnI_4 thin film is around 45 nm. (c) AFM image of PEA_2SnI_4/C_{60} thin film. (d) The corresponding depth profile shows that the thickness of the PEA_2SnI_4/C_{60} thin film is approximately 55 nm. The thickness of C_{60} layer can be calculated as 10 nm.

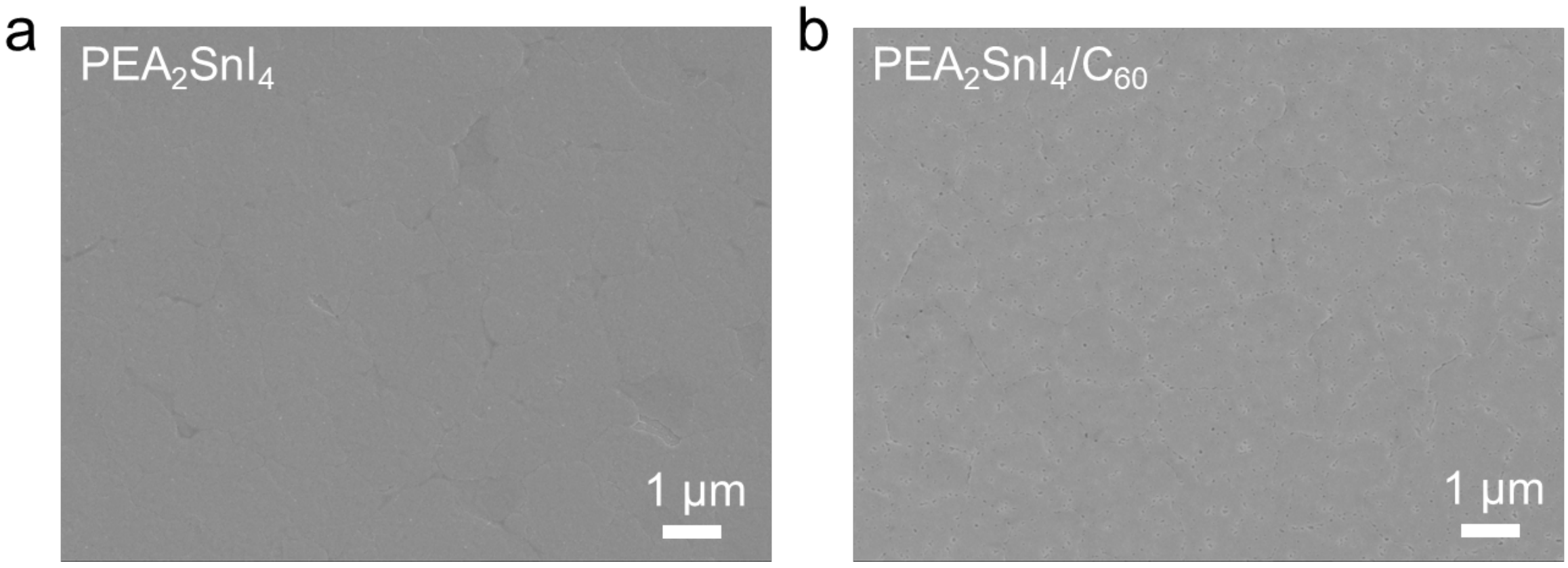

Figure S6. SEM image of (a) PEA_2SnI_4 thin film, (b) PEA_2SnI_4/C_{60} thin film.

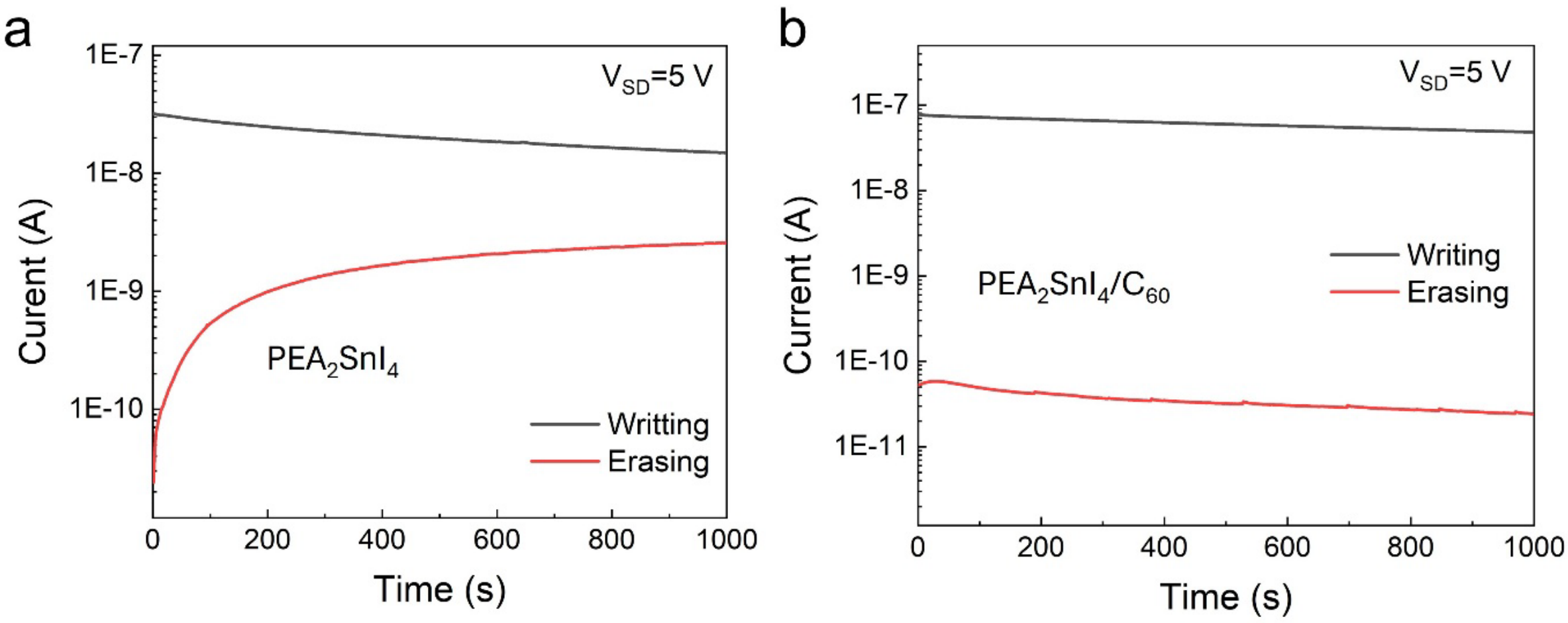

Figure S7. Writing (V_g= -20 V) and erasing (V_g= 20 V) program on (a) PEA_2SnI_4 and (b) PEA_2SnI_4/C_{60} devices. The on/off ratio of the PEA_2SnI_4 device drops to around 10 after 1000 s, while the on/off ratio of PEA_2SnI_4/C_{60} device maintain >10^3 after 1000 s.

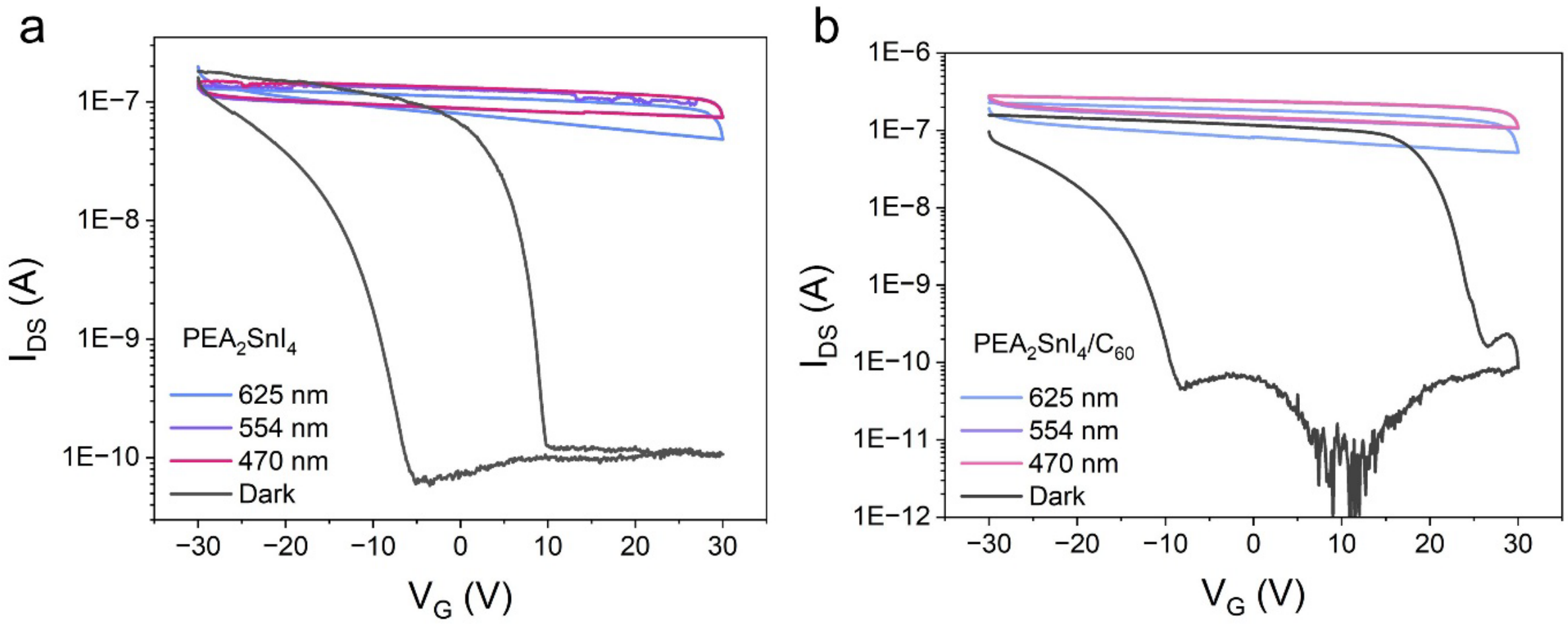

Figure S8. Transfer curve of (a) PEA_2SnI_4 device and (b) PEA_2SnI_4/C_{60} device under 470 nm, 554 nm, 625 nm illumination. Light intensity = 0.08 mW/cm^2, V_{DS} = 5 V, both the devices can not be turned off under visible light illumination.

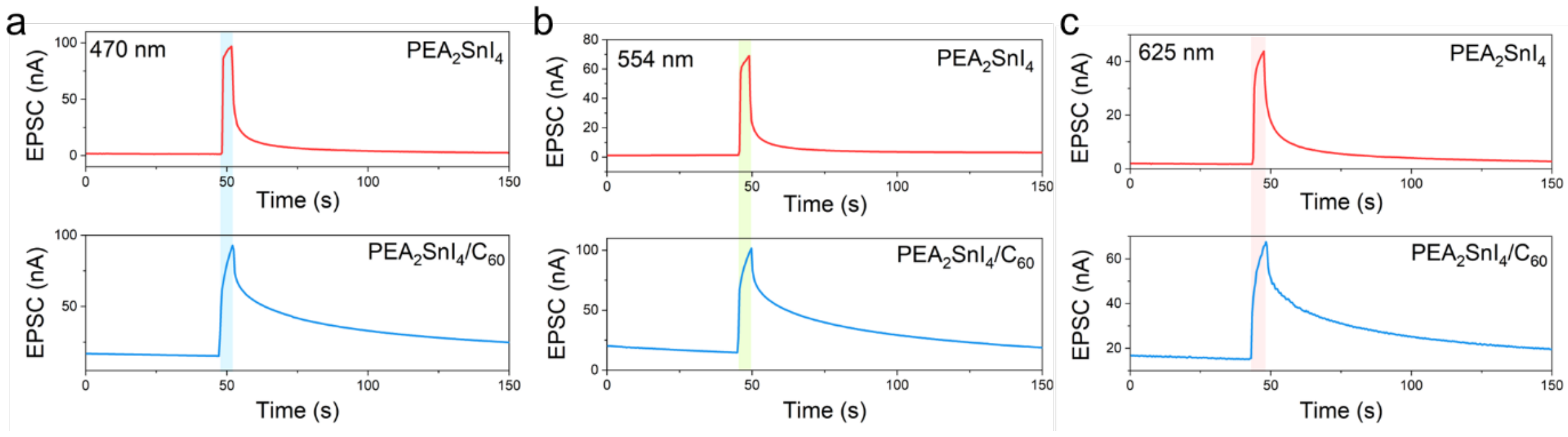

Figure S9. Synaptic performance of the PEA_2SnI_4 device and PEA_2SnI_4/C_{60} device under single light pulse (a) 470 nm, (b) 554 nm, (c) 625 nm with intensity at 2.7 mW cm^{-2} and 3 s duration. Bias = 1 V. The conductance of the PEA_2SnI_4 single-layer device decays rapidly after light illumination is turned off, showing a 90% decrease within 10 s. In contrast, the PEA_2SnI_4/C_{60} heterostructure device exhibits pronounced longer retention, with only a 53% drop over the same period. This difference indicates that the heterojunction enhances charge trapping, thereby extending the memory retention time.

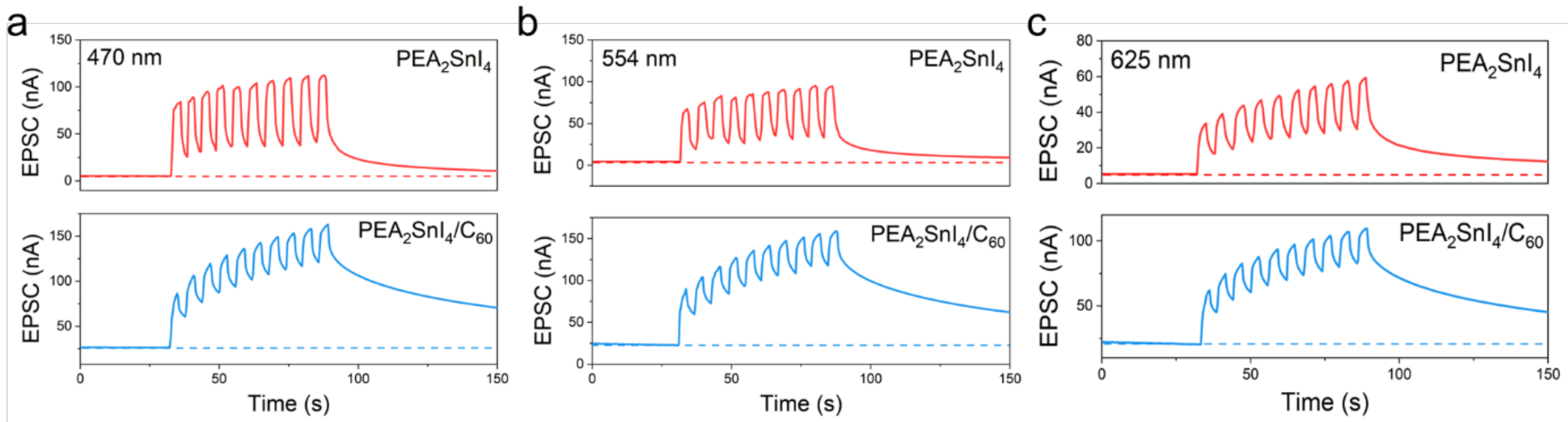

Figure S10. Synaptic performance of the PEA_2SnI_4 device and PEA_2SnI_4/C_{60} device under 10 light pulses (a) 470 nm, (b) 554 nm, (c) 625 nm with intensity at 2.7 mW cm^{-2}, 3 s on, 3 s off. Bias = 1 V. Following 10-pulse optical stimulation, the current is observed to decay to 5% of its peak in the PEA_2SnI_4 device after 60 s. Under the same conditions, the PEA_2SnI_4/C_{60} device maintains 35% of its peak current, indicating improved charge retention in the heterostructure.

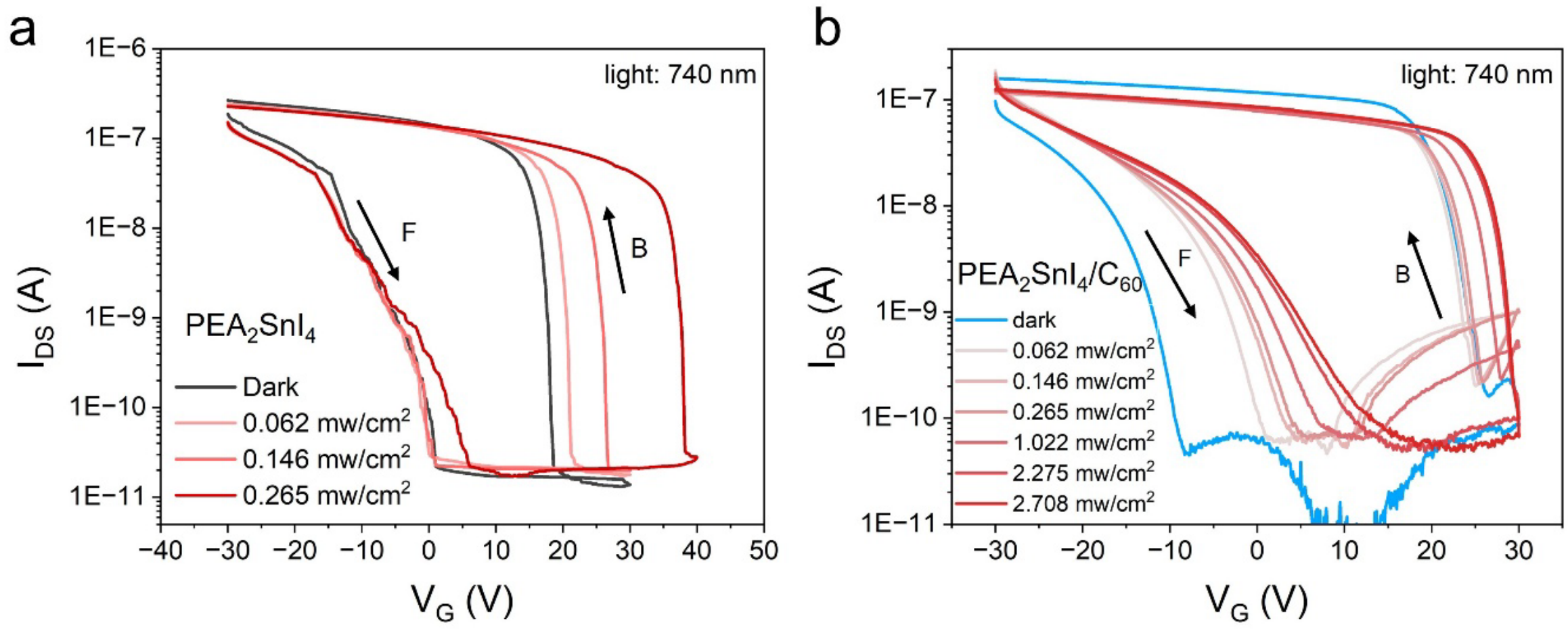

Figure S11. Transfer curve of (a) PEA_2SnI_4 device and (b) PEA_2SnI_4/C_{60} device under 740 nm light illumination with different light intensity. (F: forward sweep. B: backward sweep). V_{DS}= 5 V.

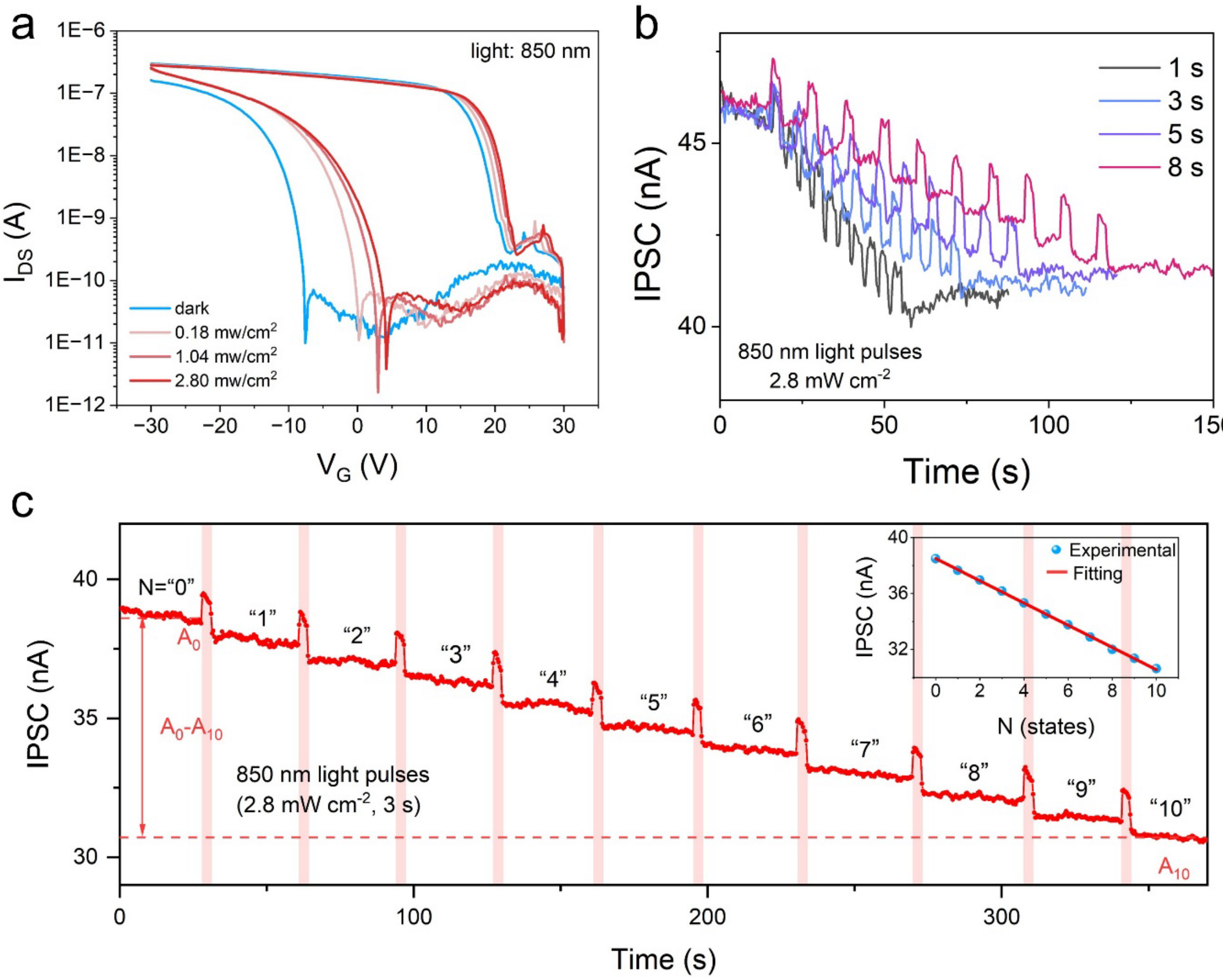

Figure S12. Synaptic performance of the PEA_2SnI_4/C_{60} device under 850 nm illumination. (a) Transfer curve at V_{DS} = 5 V under 850 nm light illumination with different light intensities. The hysteresis loop window is narrowed with the increase of 850 nm light intensity. (b) IPSC under 10 light pulses with 3s on and different off duration range from 1 s to 8 s. Bias = 1 V. (c) 10 states IPSC under light pulse (3 s on, 30 s off). Bias = 1 V.

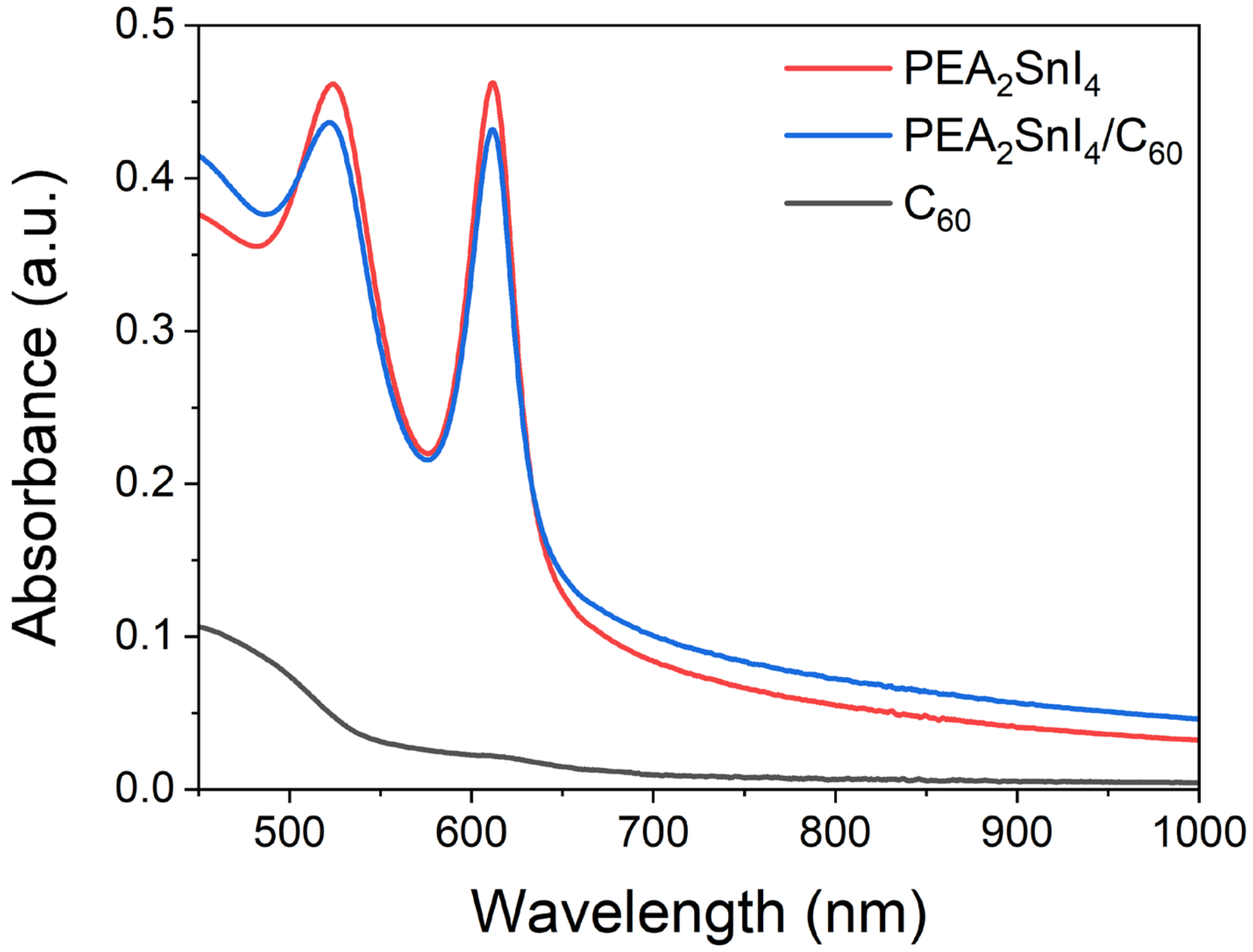

Figure S13. UV-vis spectra of PEA_2SnI_4, PEA_2SnI_4/C_{60}, and C_{60} thin films. The PEA_2SnI_4/C_{60} film shows a higher residual absorption tail in the near-infrared region, suggesting an increased sub-bandgap photo response.

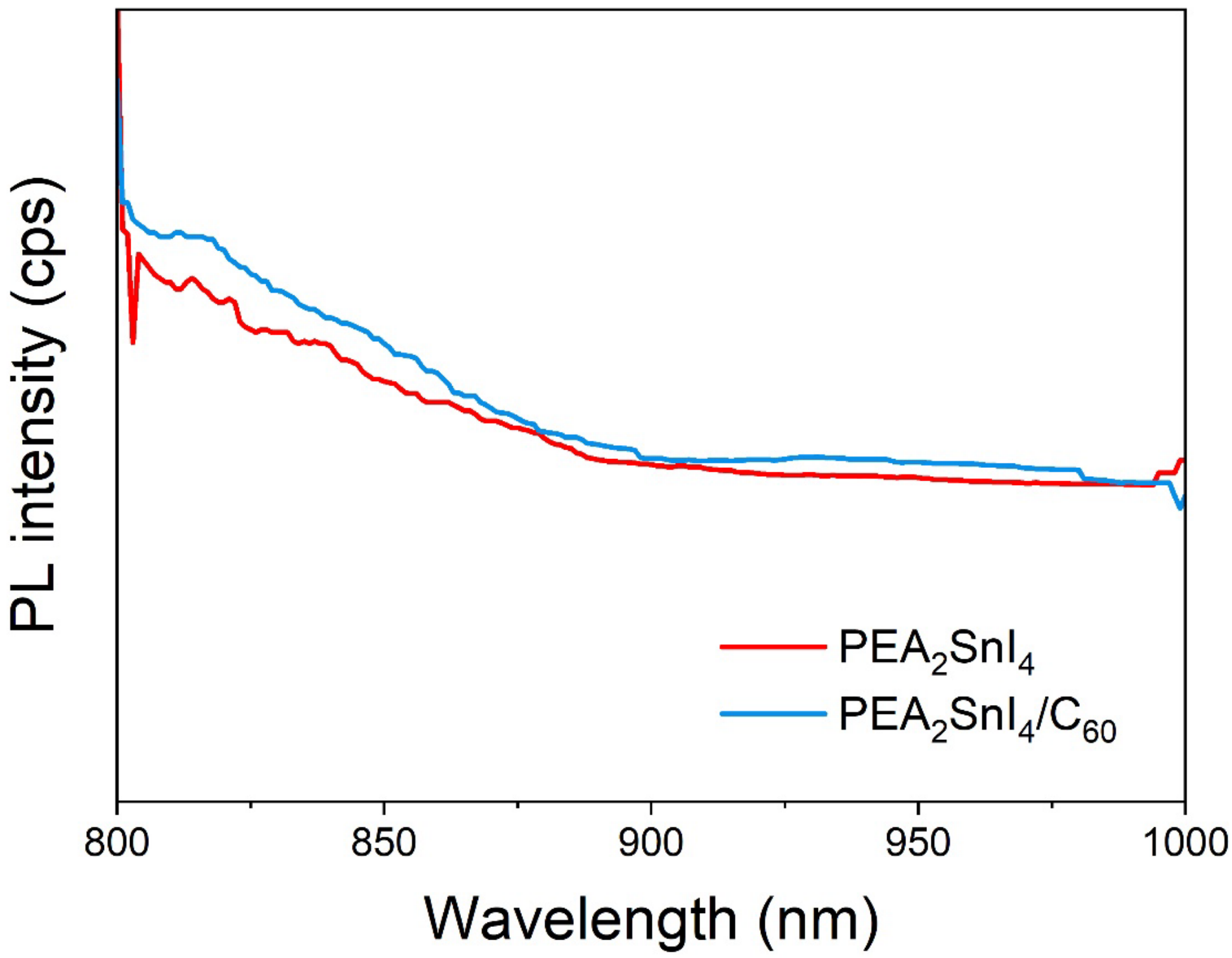

Figure S14. PL spectra in NIR region (800 nm-1000 nm).

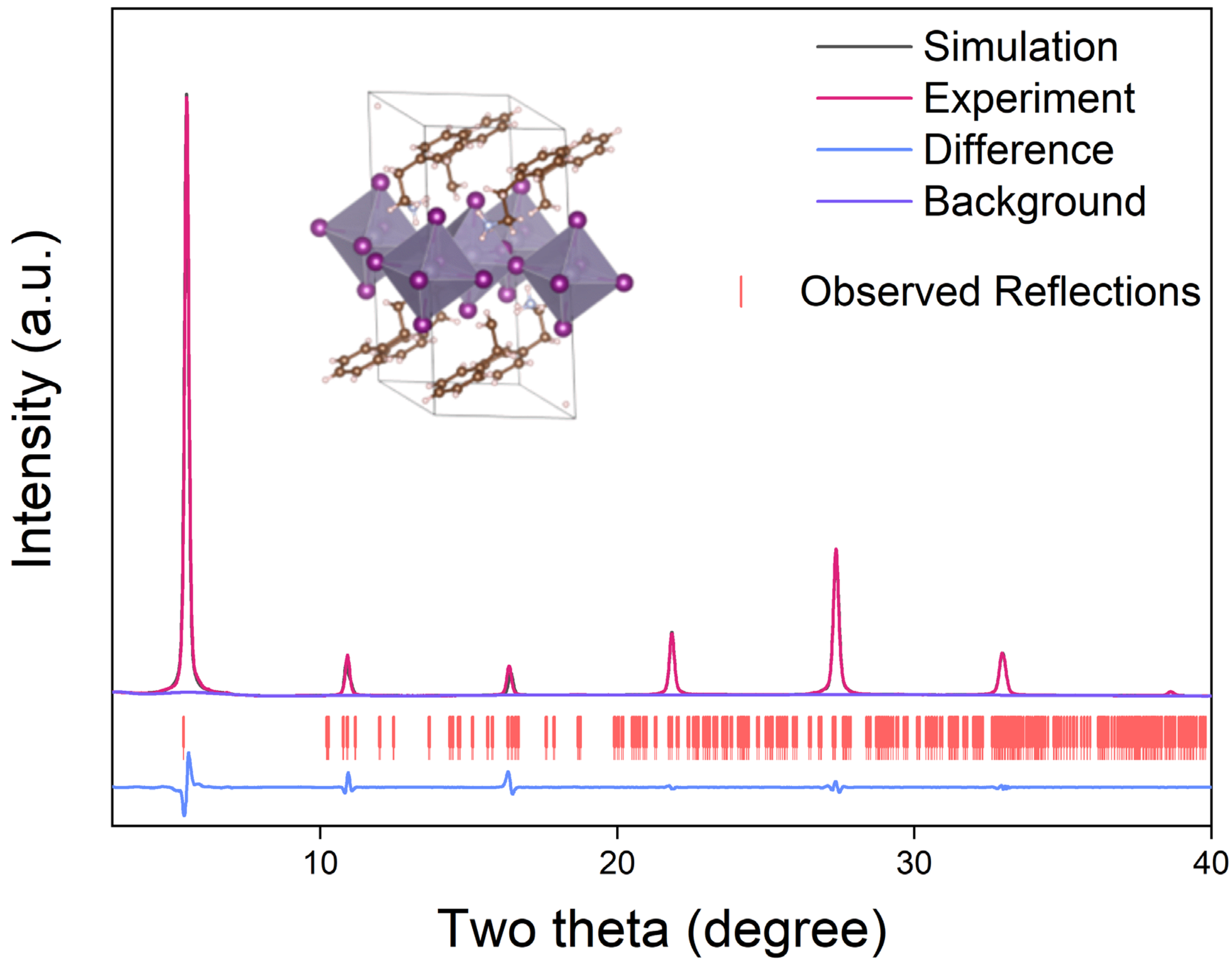

Figure S15. Refined XRD patterns with the satisfaction of Powder Refinement: R_{wp}=11.36% R_{wp}(w/o bck) = 13.57% R_p = 7.29%. Insert: the refined lattice structure.

The following are the refined lattice parameters of SnI_4PEA_2

Parameters	a	b	c	alpha	beta	gamma
value	8.65921	8.605976	16.377915	94.752	99.958	89.965

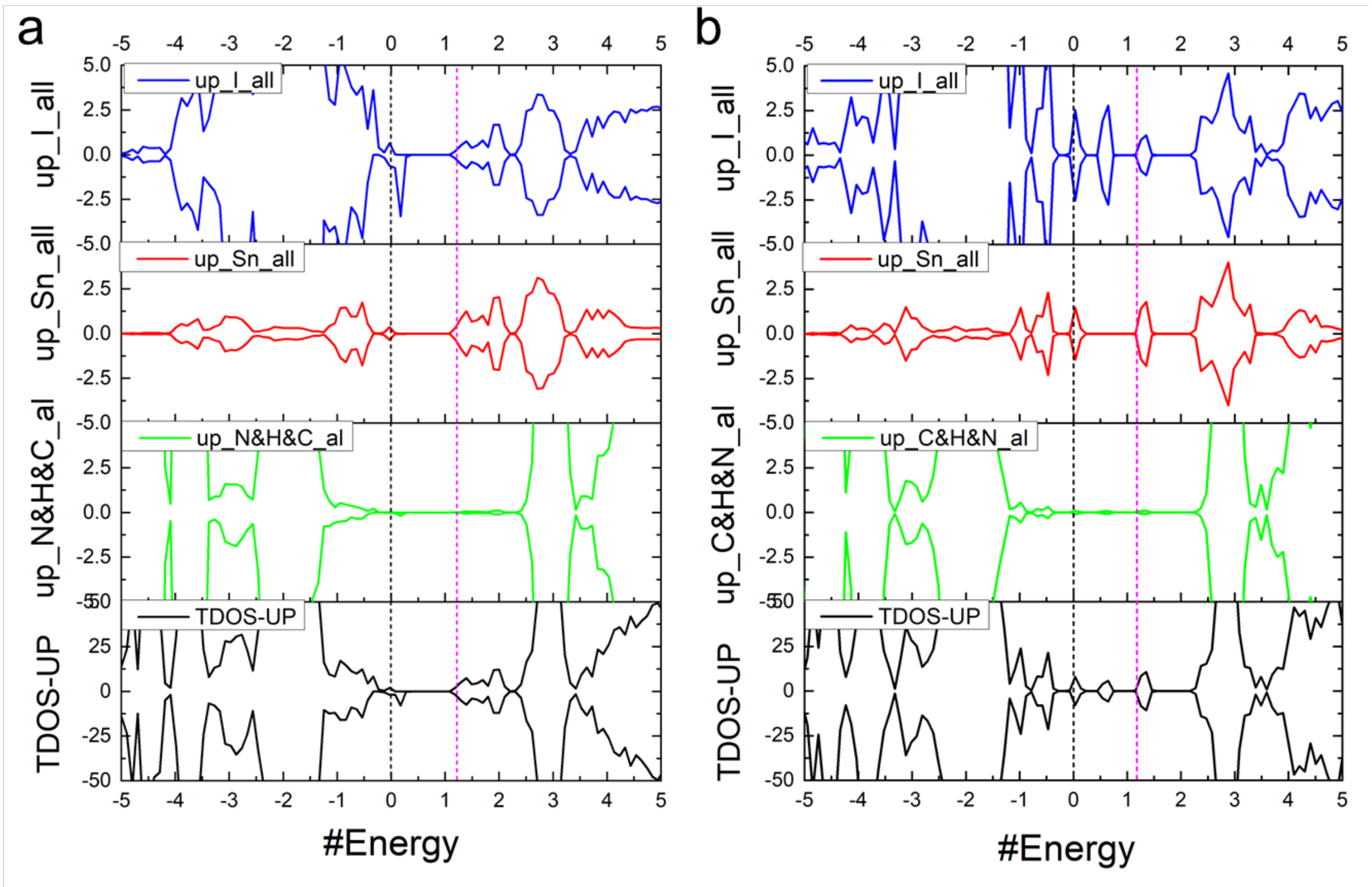

Figure S16. The calculated DOS of two interstitials (a) i_I (b) i_{Sn}.

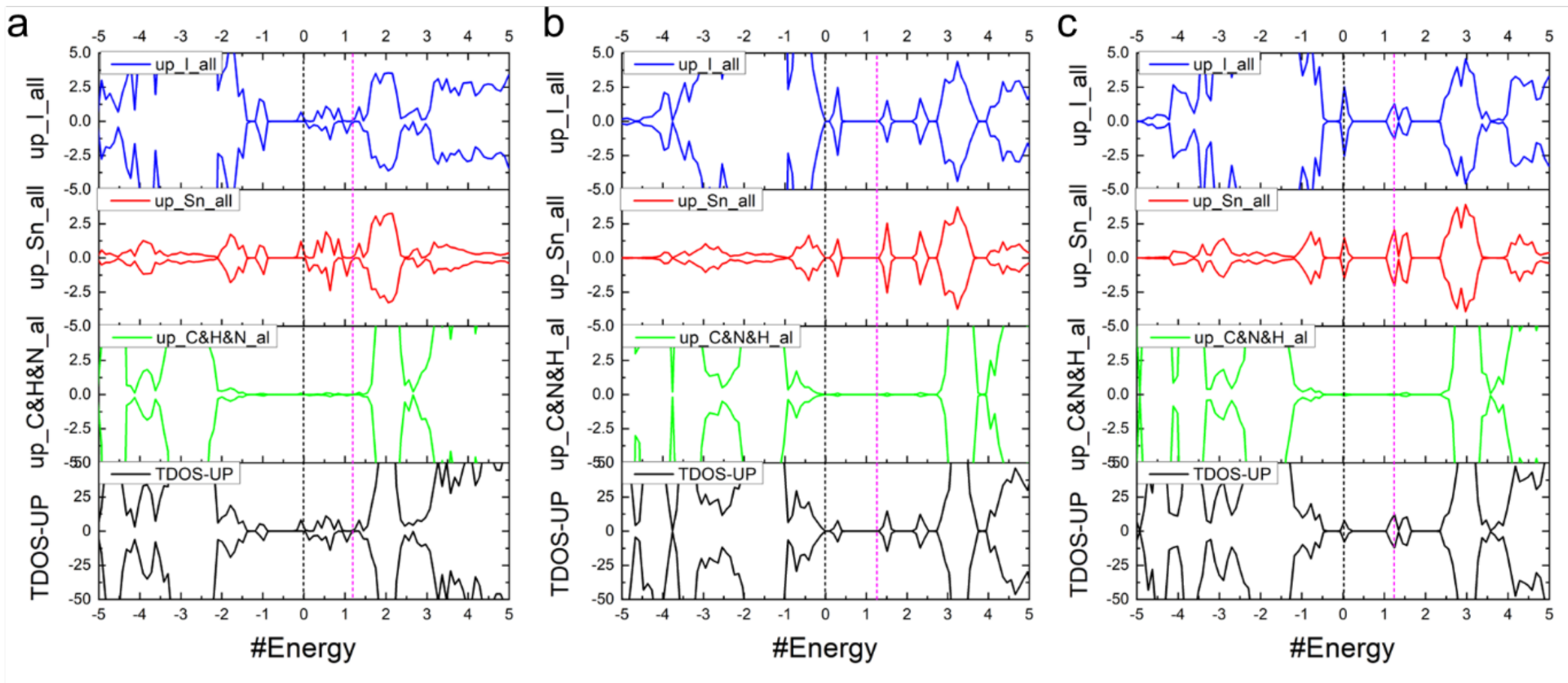

Figure S17. The calculated DOS of vacancies of (a)V_I, (b)V_{Sn}, and (c)V_{PEA}.

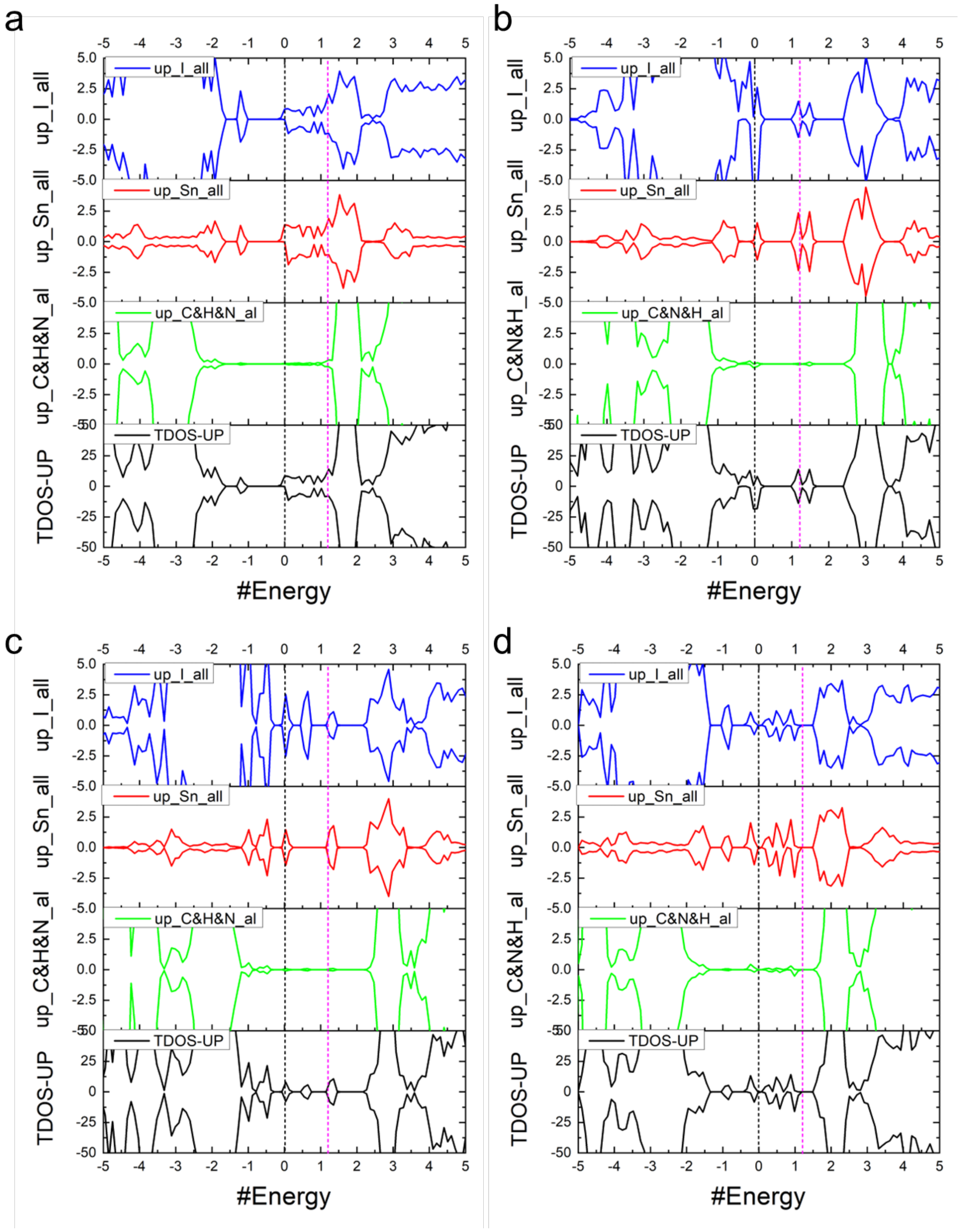

Figure S18. DOS of substitutions. Cation substitution (a) Sn_{PEA}. Anti-site substitutions (b) I_{PEA}, (c) I_{Sn}, (d) Sn_{I}

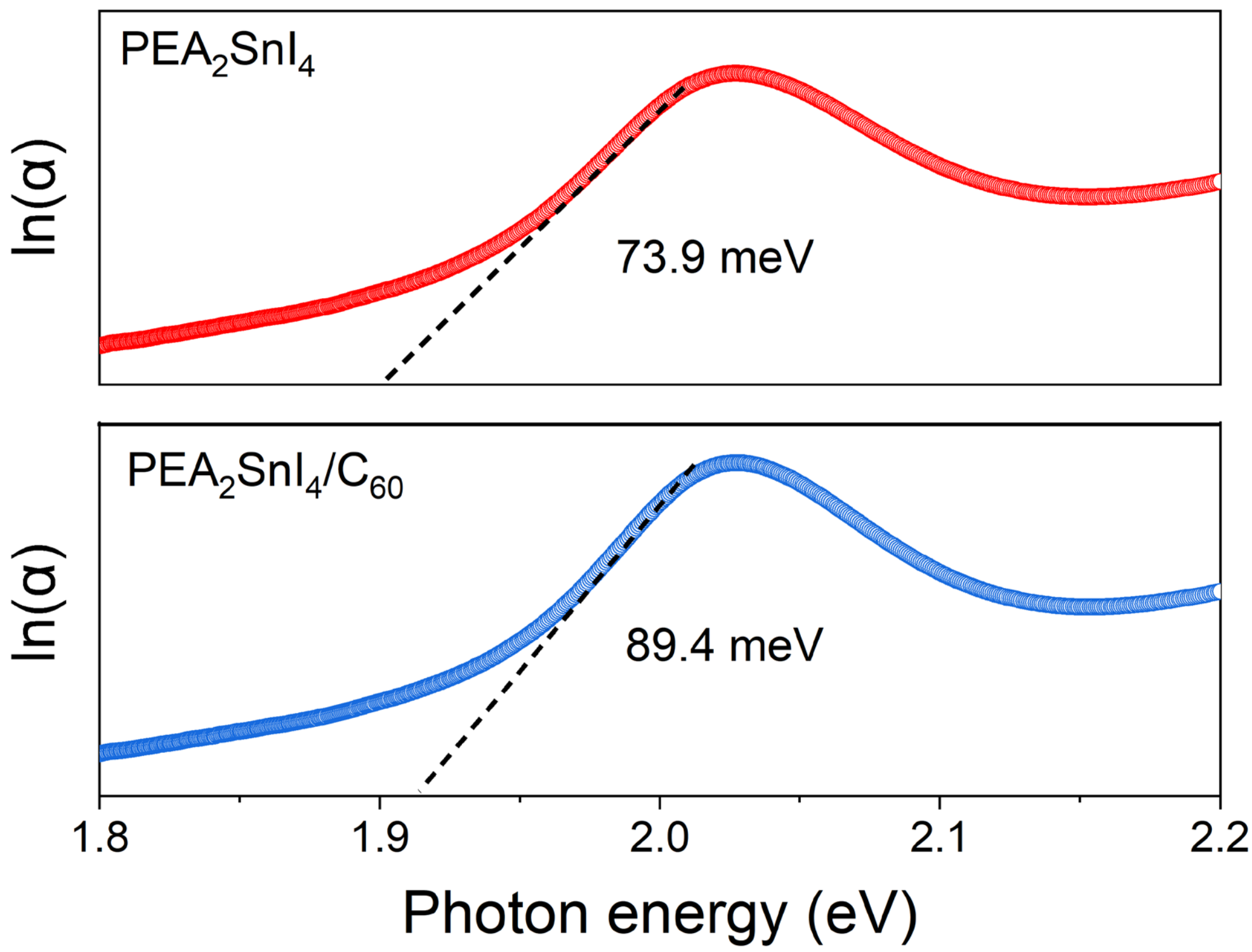

Figure S19. Urbach energy of PEA_2SnI_4 film and PEA_2SnI_4/C_{60} film.

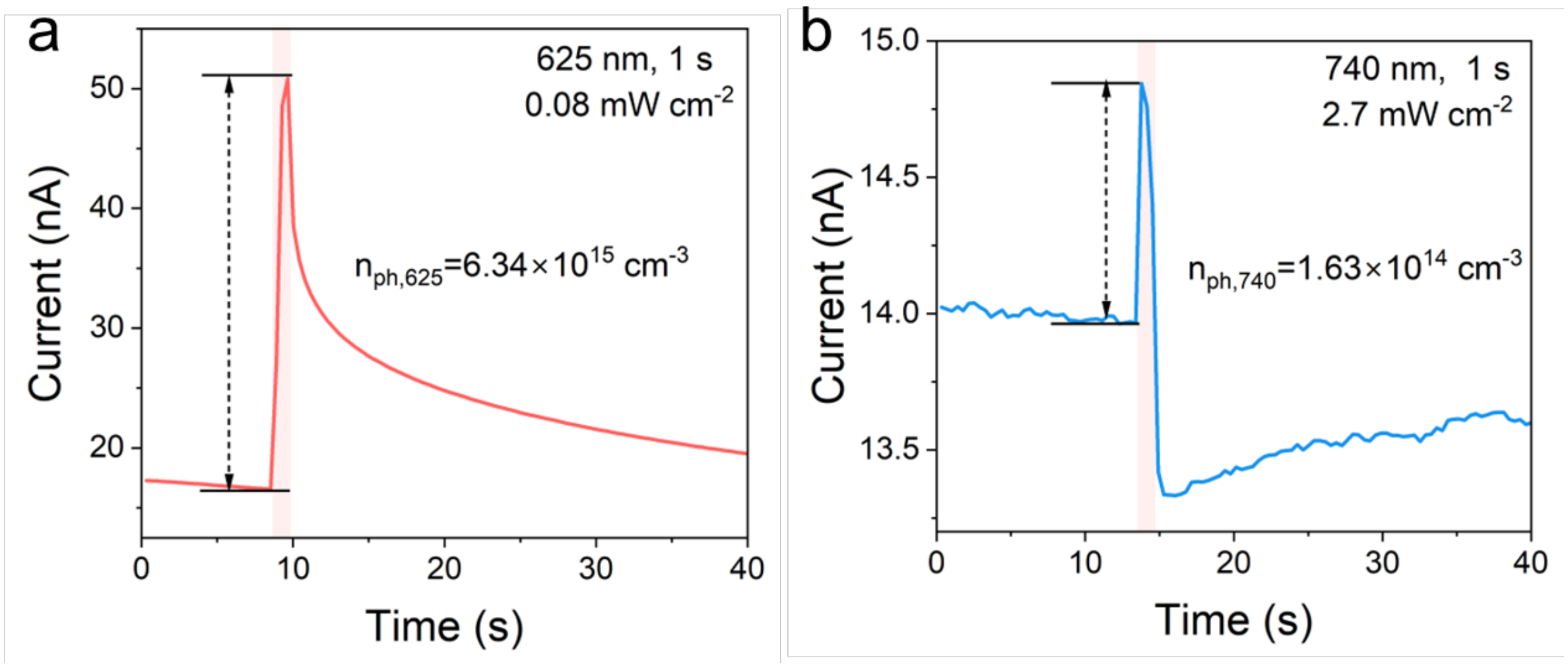

Figure S20. Effective photoexcited carrier populations of PEA_2SnI_4/C_{60} device under illumination with a single light pulse at (a) 625 nm and (b) 740 nm.

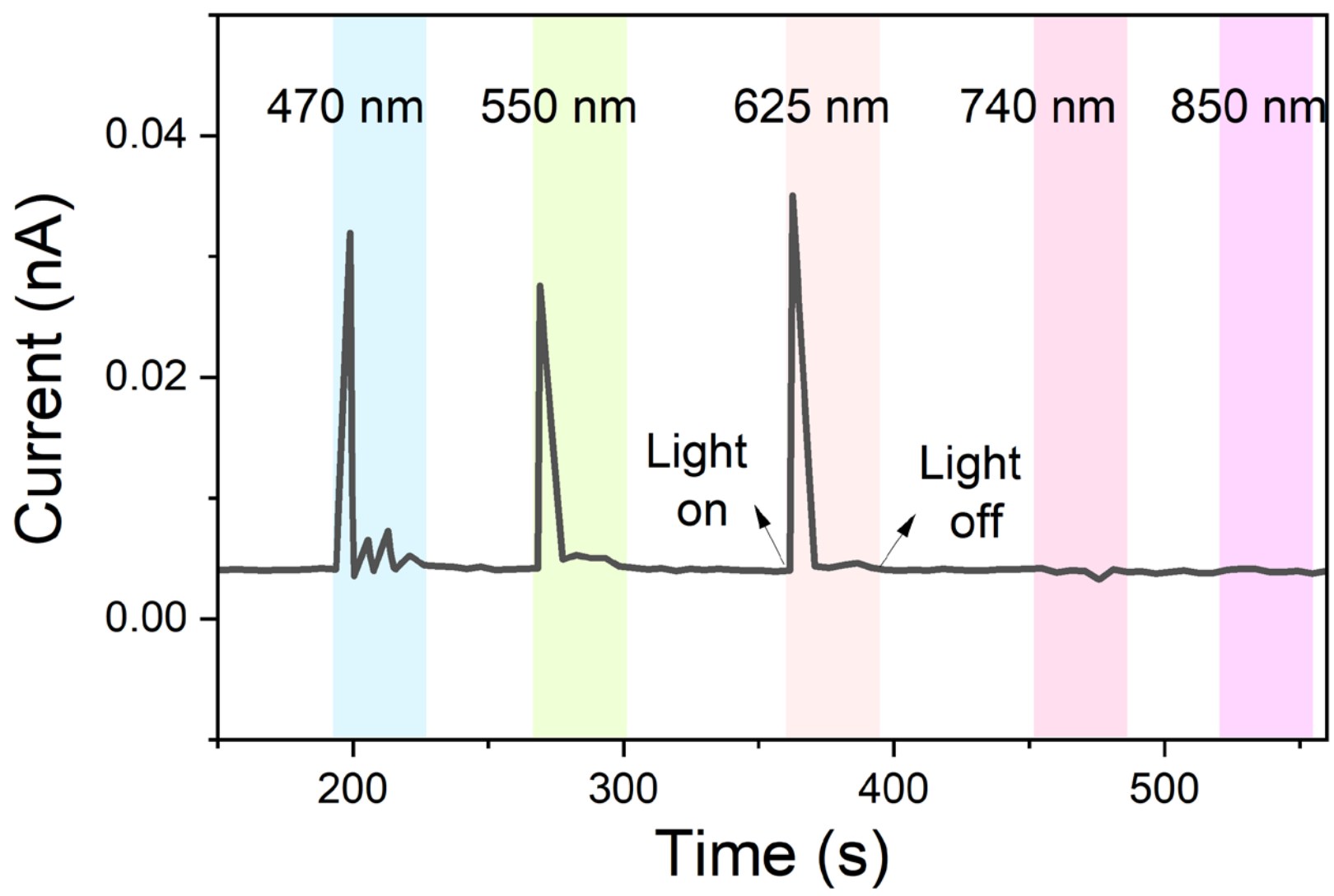

Figure S21. Light response of C_{60} layer (10 nm) under different light wavelength illuminations (470/550/625/740/850 nm). Light intensity: ~2.7 mW/cm^2, duration: 20 s. Bias: 30 V.

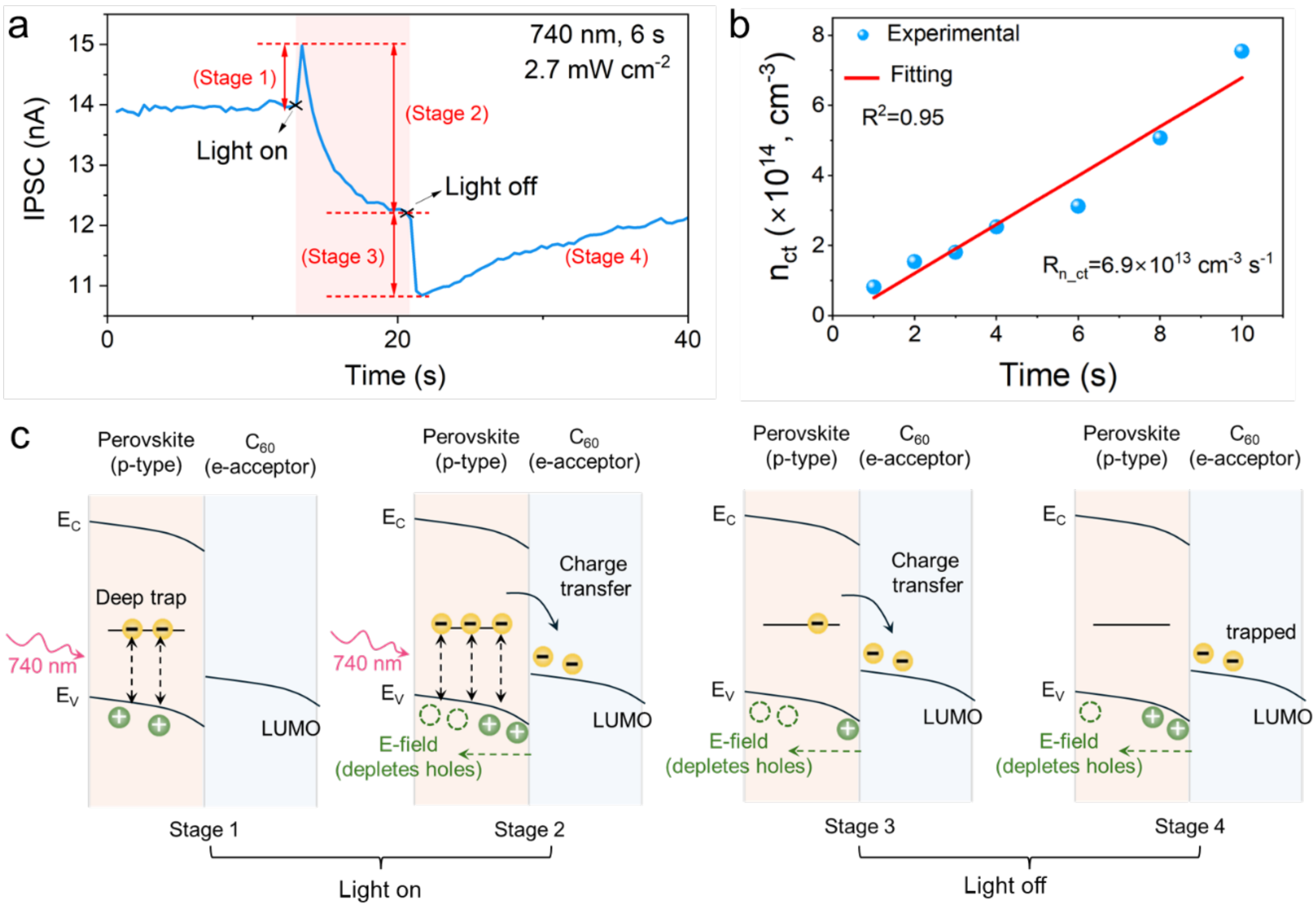

Figure S22. Carrier dynamics analysis of PEA_2SnI_4/C_{60} heterostructure under 740 nm light illumination. (a) Stages determination of photo-response under 740 nm single pulse (6 s). (b) Accumulated charge transfer populations (n_{ct}) under different light pulse widths (1 -10 s). The interfacial charge transfer rate (R_{n_ct}) is determined from the slope of the linear fitting. (c) Band-to-band diagram illustration of carrier dynamics for different stages. Upon 740 nm illumination, defect-assisted sub-bandgap absorption first generates a small population of mobile carriers, giving rise to a sharp transient current increase (stage 1). With continued illumination, interfacial charge transfer from the perovskite to C_{60} progressively dominates (stage 2). The electrons trapped in C_{60} establish a local electric field that depletes holes in the p-type perovskite channel, thereby reducing the channel current. After the light is switched off, the photogenerated carriers recombine rapidly, while the trapped electrons persist and continue to suppress the hole population, resulting in a further current drop (stage 3). The subsequent slow recovery originates from the gradual release or relaxation of trapped electrons, which weakens the interfacial field and restores the channel conductance (stage 4).

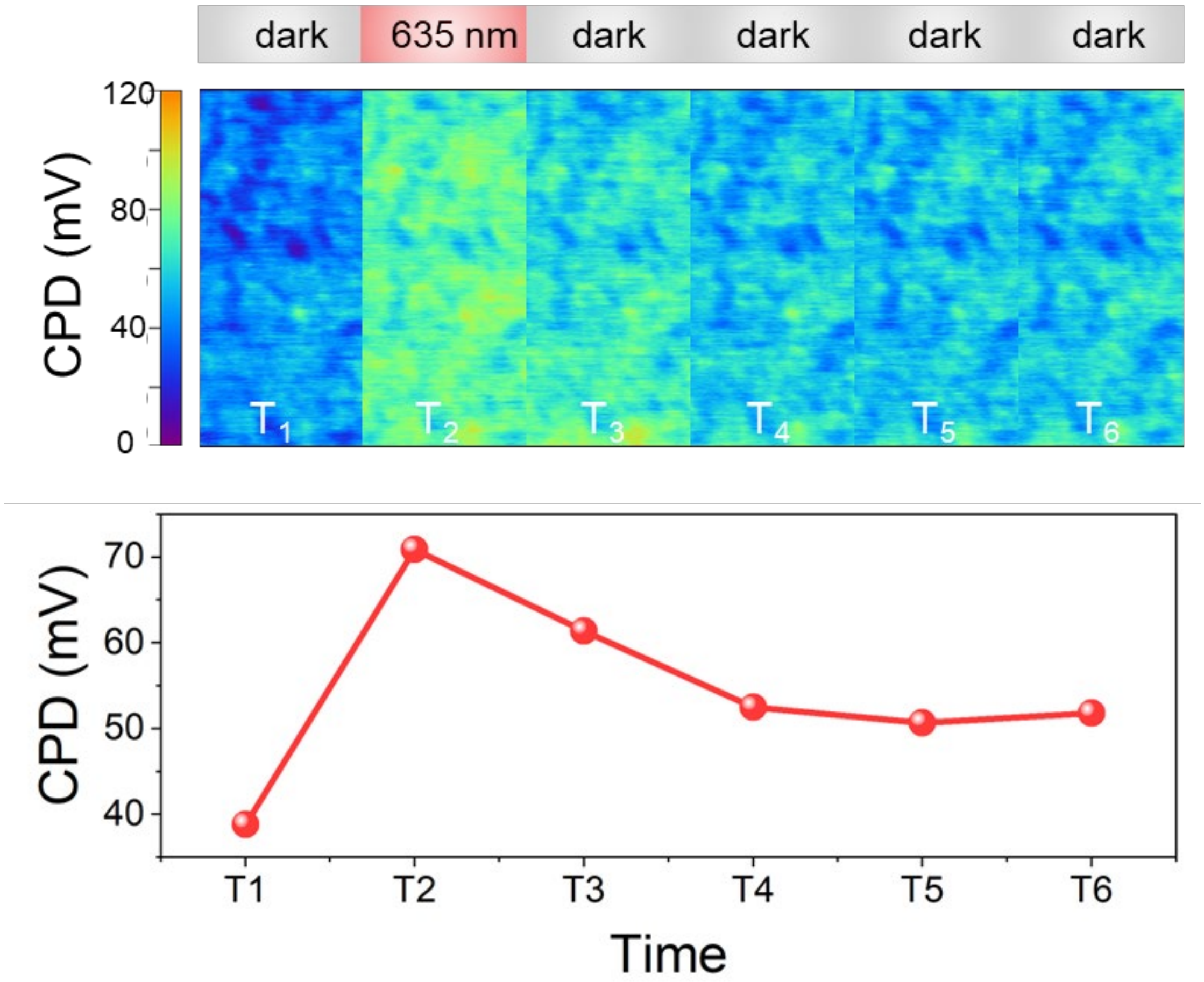

Figure S23. KPFM of PEA_2SnI_4/C_{60} device under 635 nm light illumination.

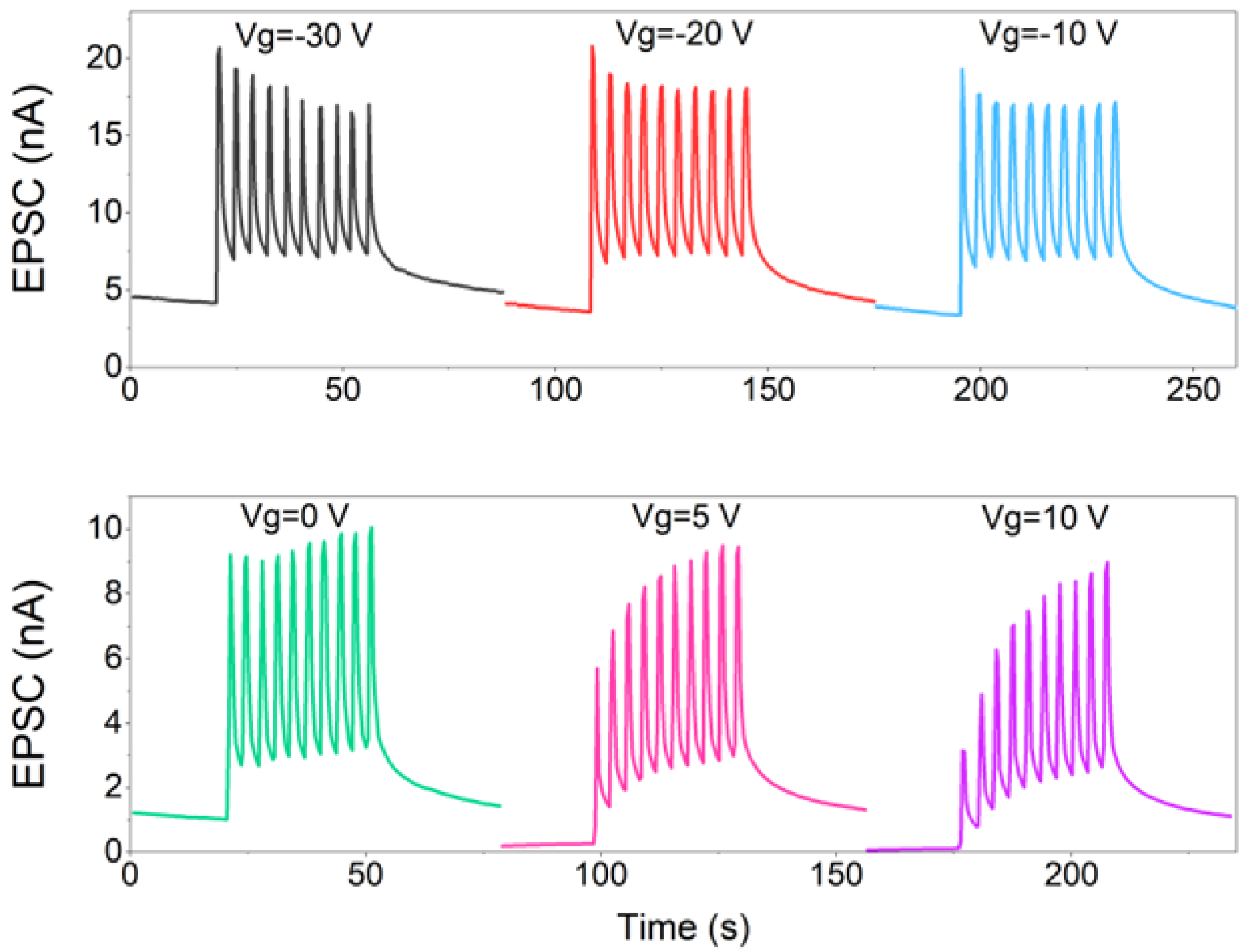

Figure S24. EPSC of the PEA_2SnI_4/C_{60} device at different V_G ranges from -30 V to 10 V under 625 nm illumination. Light intensity = 0.08 mW/cm^2, bias = 1 V.

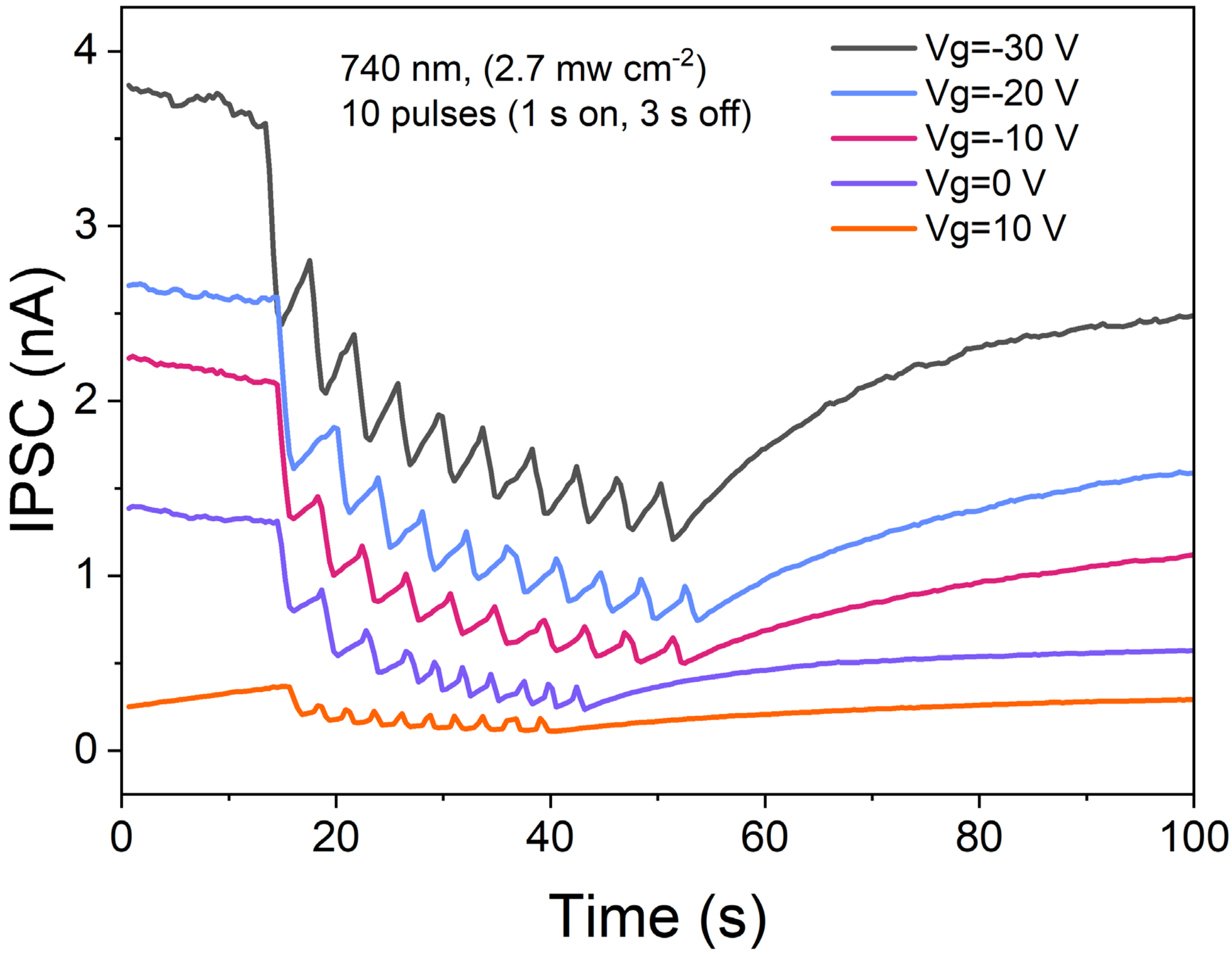

Figure S25. IPSC of the PEA_2SnI_4/C_{60} device at different V_G ranges from -30 V to 10 V under 740 nm illumination. Light intensity = 2.7 mW/cm^2, bias = 1 V.

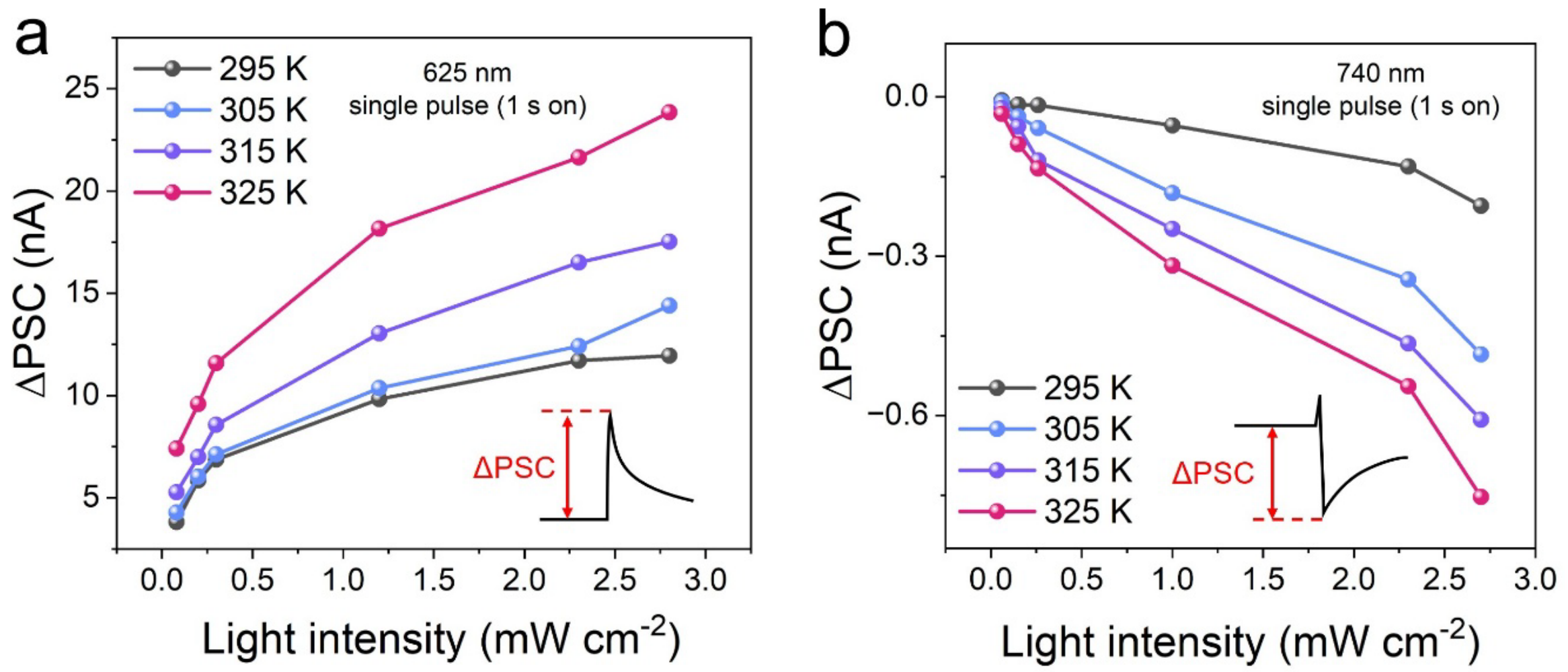

Figure S26. Temperature-dependent (295 K to 325 K) post-synaptic current (PSC) change of PEA_2SnI_4/C_{60} heterostructure under a single light pulse of (a) 625 nm (0.08 mW/cm^2) and (b) 740 nm (2.7 mW/cm^2), respectively. Bias: 1V.

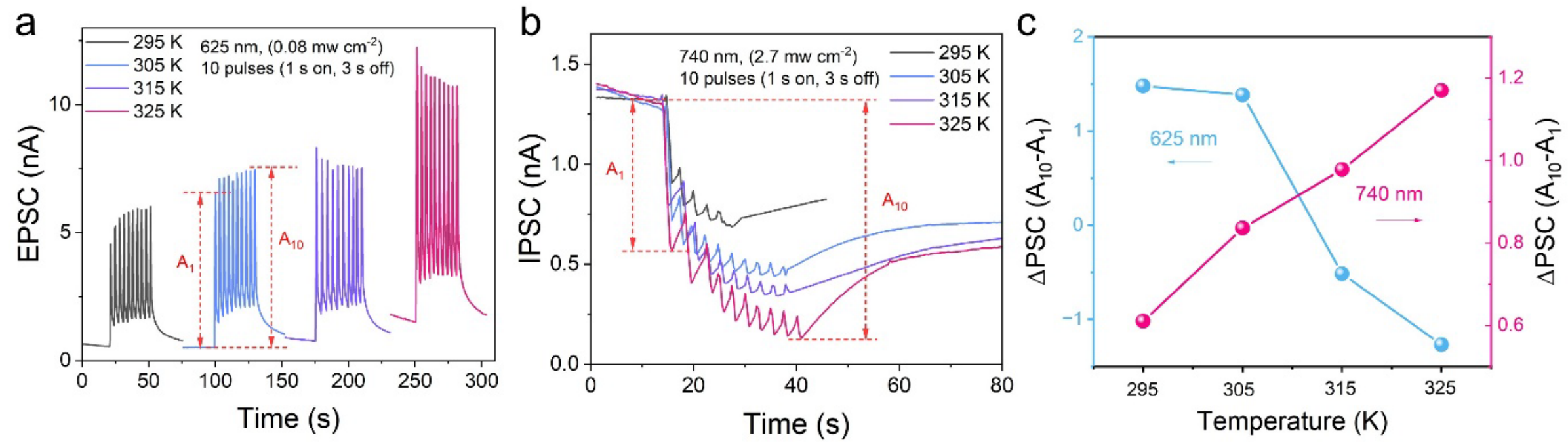

Figure S27. Temperature-dependent (295 K to 325 K) post-synaptic current change of PEA_2SnI_4/C_{60} heterostructure under 10 light pulses of (a) 625 nm (0.08 mW/cm^2) and (b) 740 nm (2.7 mW/cm^2), respectively. Bias: 1V. (c) Summary of temperature-dependent synaptic potentiation and depression change (A_{10}-A_1).

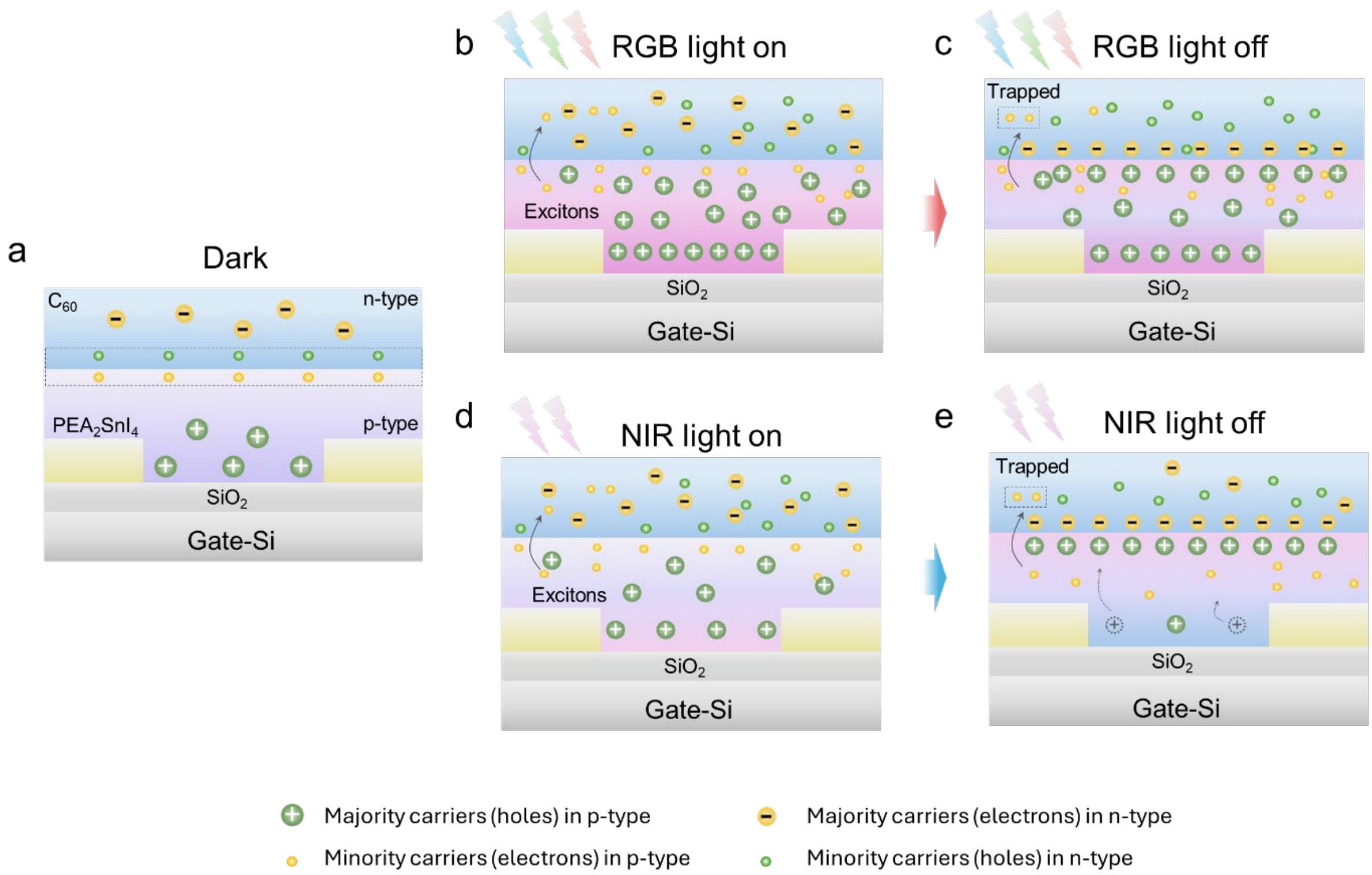

Figure S28. Schematic illustration of the proposed wavelength-dependent charge dynamics under visible and NIR illumination. (a) Carrier distribution of the p-type perovskite/C_{60} heterostructure under dark. (b) Under RGB/visible-light illumination, band-to-band excitation in the p-type perovskite generates abundant photogenerated carriers, leading to an excitatory photoresponse, while a small fraction of the photogenerated electrons transfers to the C_{60} layer at the same time. (c) After the visible light is turned off, a large population of carriers remains trapped in the perovskite channel and gradually recombines, giving rise to a persistent excitatory state. (d) Under NIR illumination, sub-bandgap absorption associated with deep-level states produces only a small number of photogenerated carriers, and the photogenerated electrons are efficiently transferred to and trapped in the C_{60} layer. (e) After the NIR light is turned off, the electrons are still trapped in the C_{60} layer, forming an interfacial potential barrier/electric field that depletes holes in the p-type perovskite channel and results in an inhibitory response.

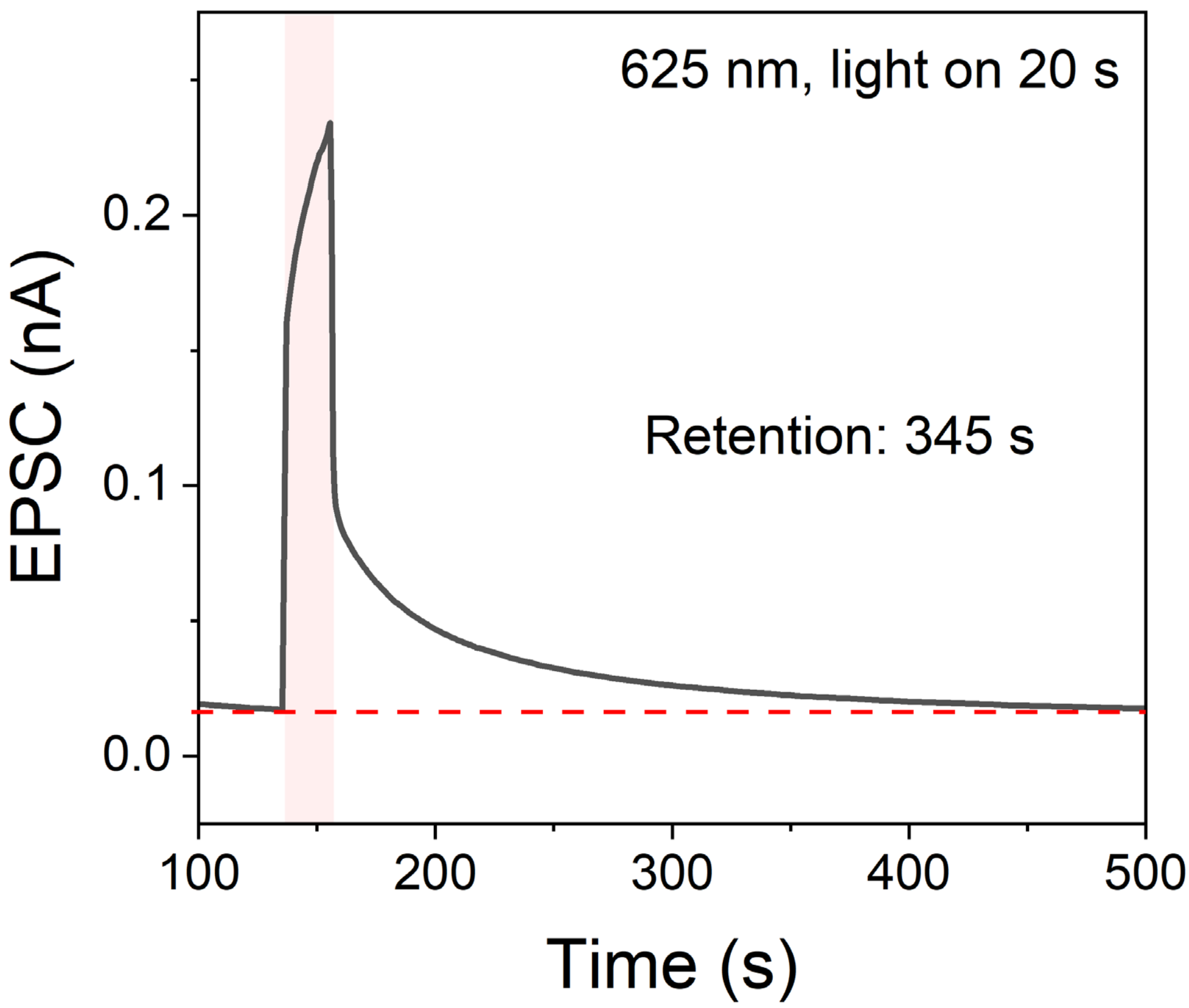

Figure S29. EPSC under a single 625 nm light pulse with duration of 20 s. The retention time is 345 s. Light intensity = 0.08 mW/cm^2, bias = 0.1 V.

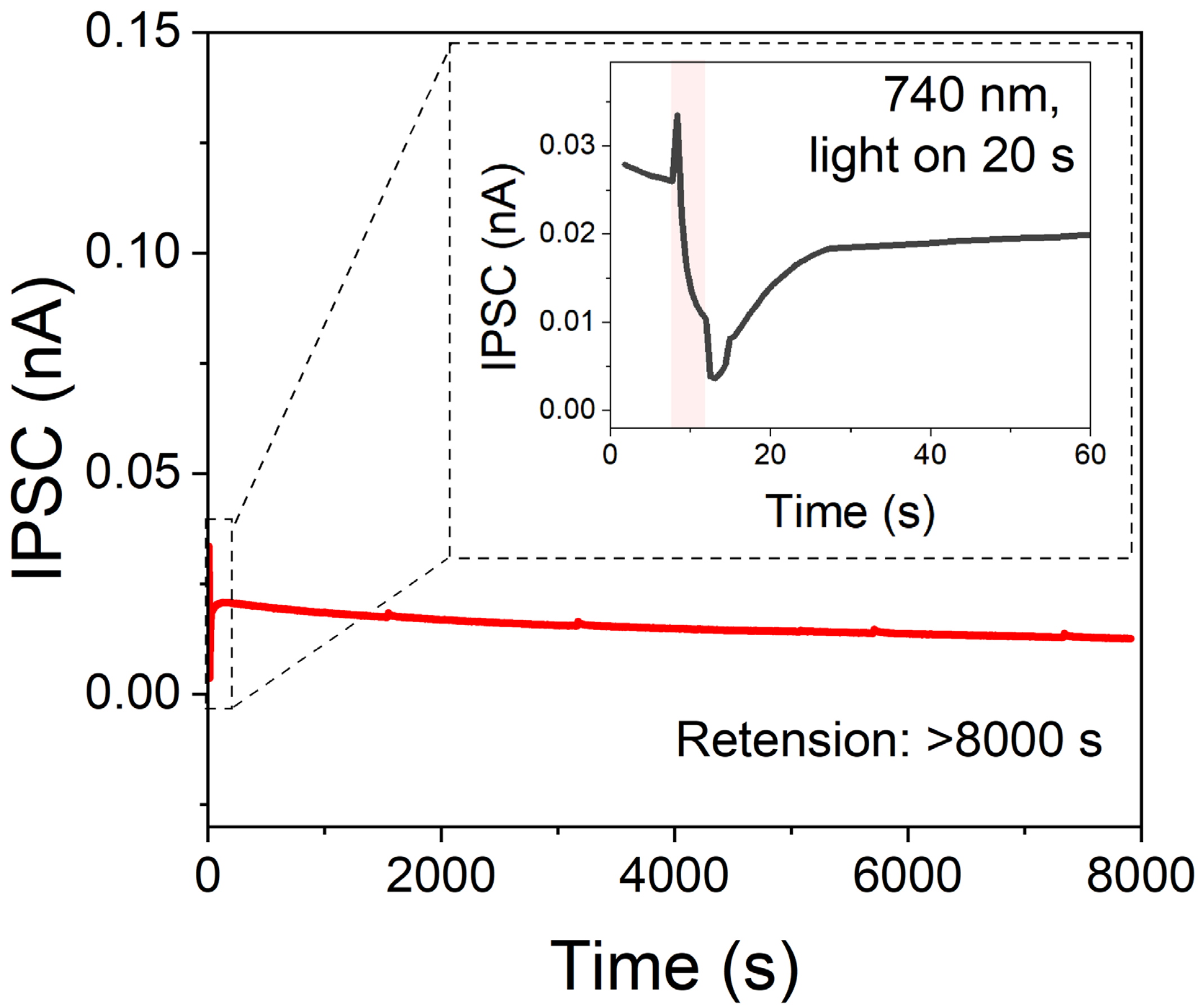

Figure S30. IPSC under a single 740 nm light pulse with duration of 20 s. The retention time is more than 8000 s, demonstrating long-term memory. Light intensity = 2.7 mW/cm^2, bias = 0.1 V.

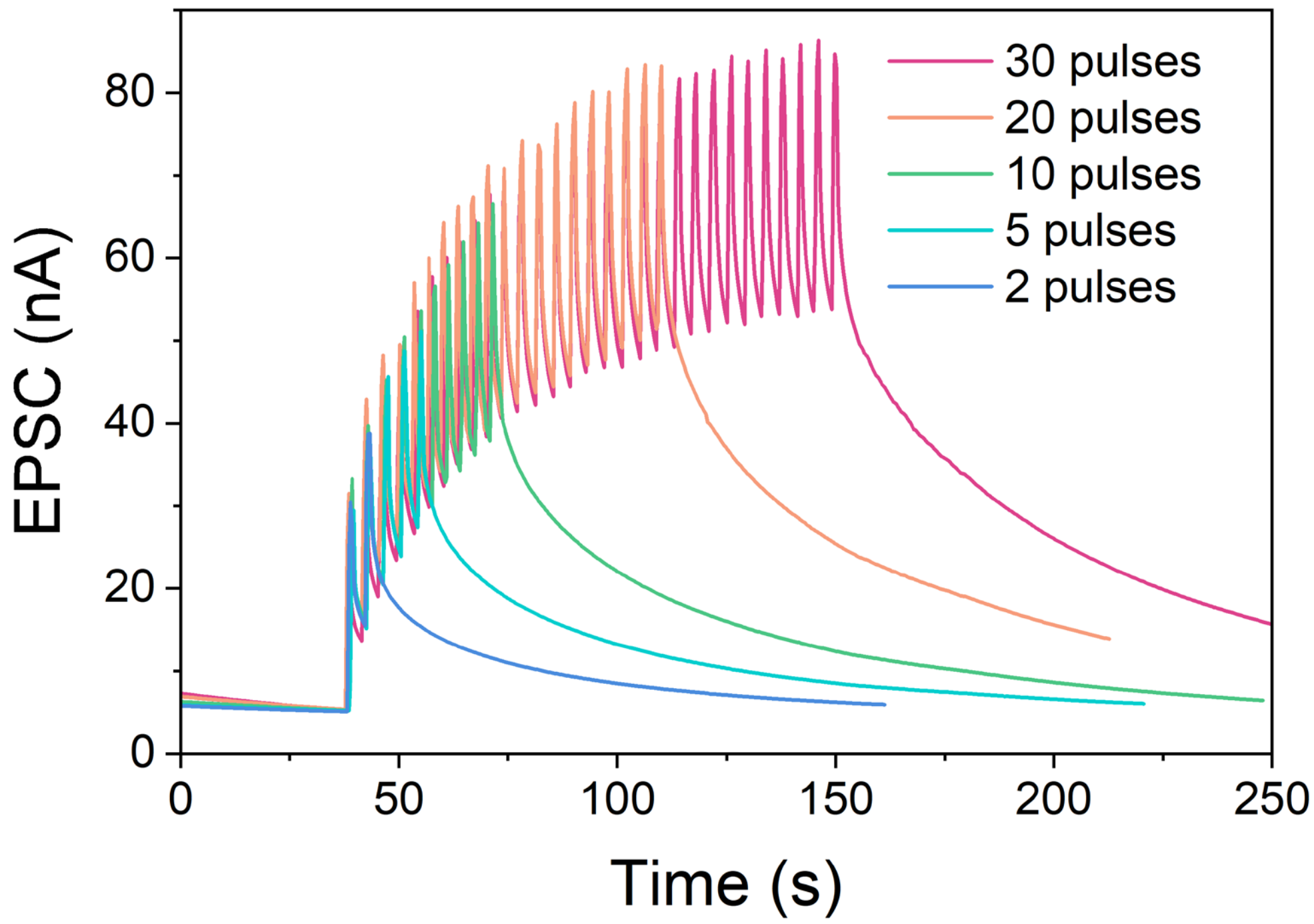

Figure S31. EPSC under 625 nm light pulses with different pulse numbers. Light intensity = 0.08 mW/cm^2, bias = 1 V. Light pulse: 2 s on, 1 s off.

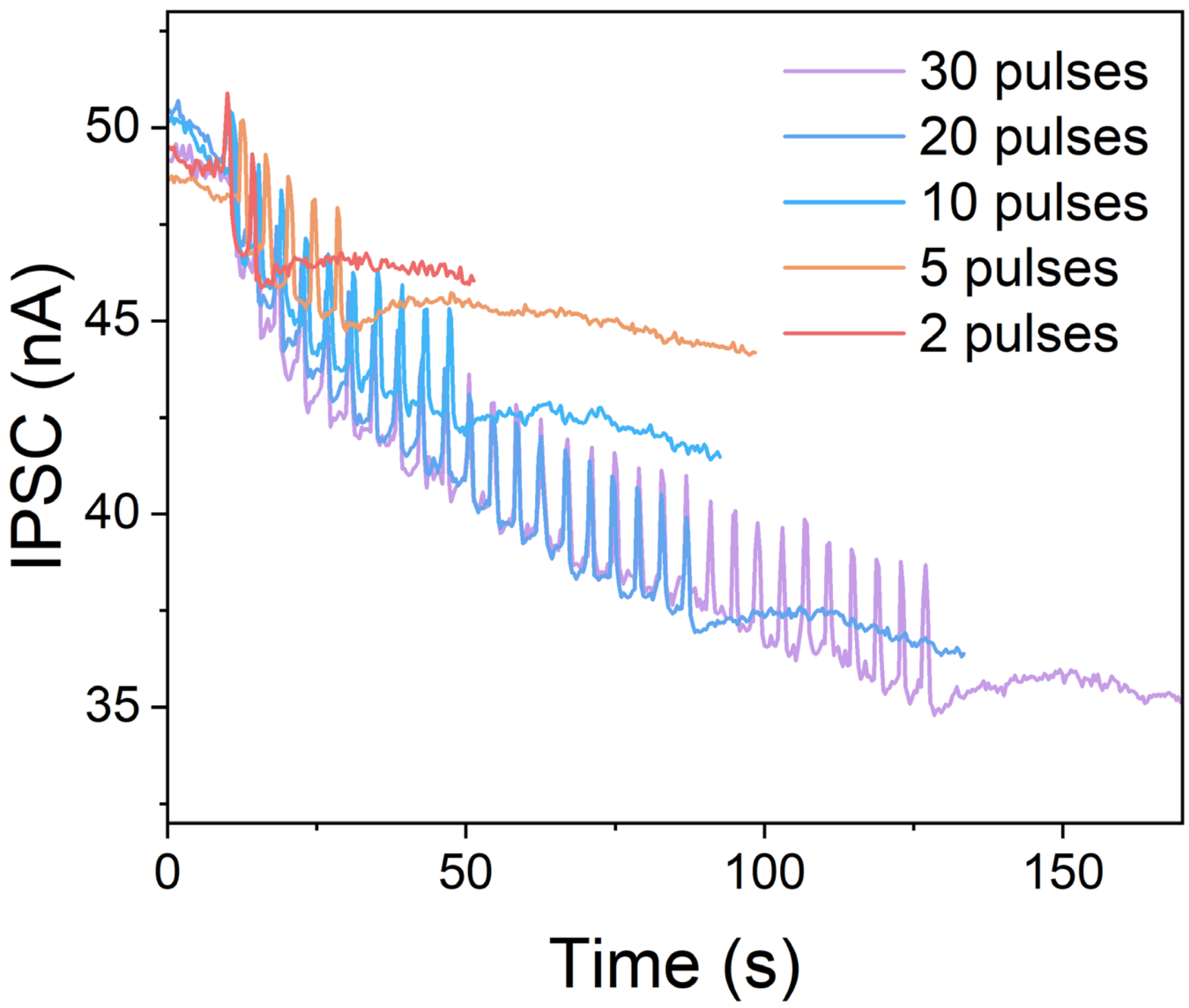

Figure S32. IPSC under 725 nm light pulses with different pulse numbers. Light intensity = 2.7 mW/cm^2, bias = 1 V. Light pulse: 2 s on, 1 s off.

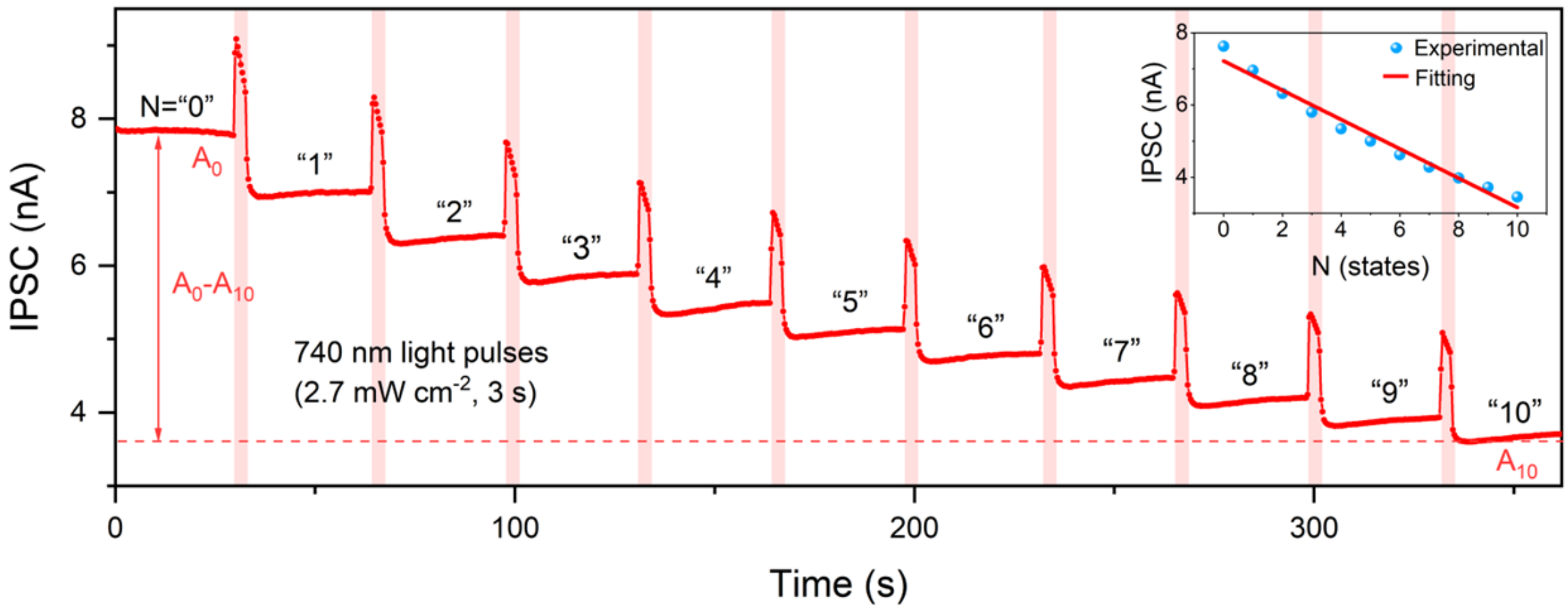

Figure S33. IPSC under ten light pulses of 740 nm (2.7 mW cm^{-2}) with 3 s on, 30 s off. Ten different states can be obtained. Bias = 1 V.

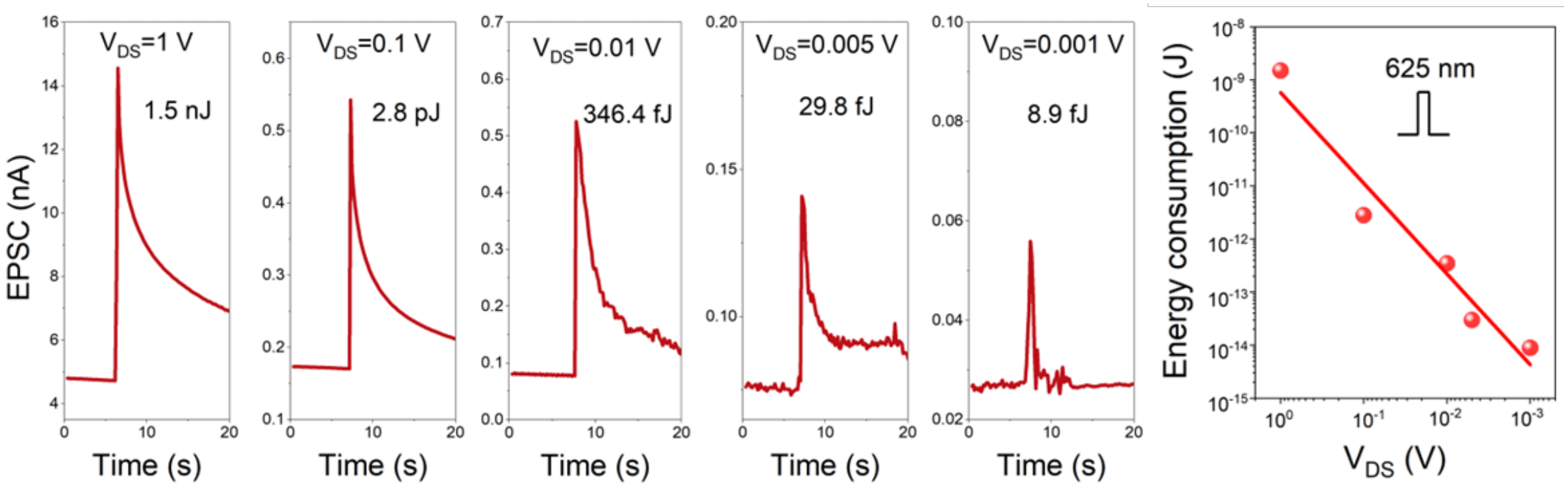

Figure S34. Energy consumption of PEA_2SnI_4/C_{60} device under 625 nm light pulse (0.08 mW/cm^2). With a decreased source drain bias: 1V, 0.1 V, 0.01 V, 0.005 V, 0.001 V, the energy consumption is reduced: 1.5 nJ, 2.8 pJ, 346.4 fJ, 29.8 fJ, 8.9 fJ, respectively. The lowest energy consumption is 8.9 fJ.

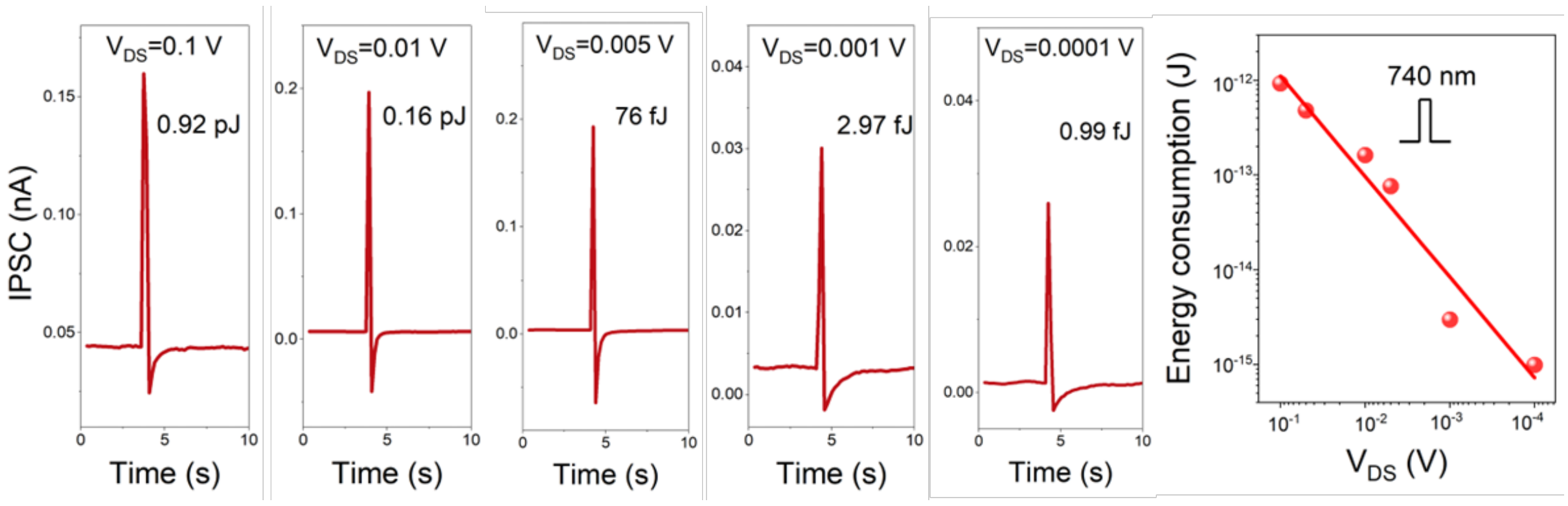

Figure S35. Energy consumption of PEA_2SnI_4/C_{60} device under 740 nm light pulse (2.7 mW/cm^2). With a decreased source drain bias: 0.1 V, 0.01 V, 0.005 V, 0.001 V, 0.0001 V, the energy consumption is reduced: 0.92 pJ, 0.16 pJ, 76 fJ, 2.97 fJ, 0.99 fJ, respectively. The lowest energy consumption is 0.99 fJ.

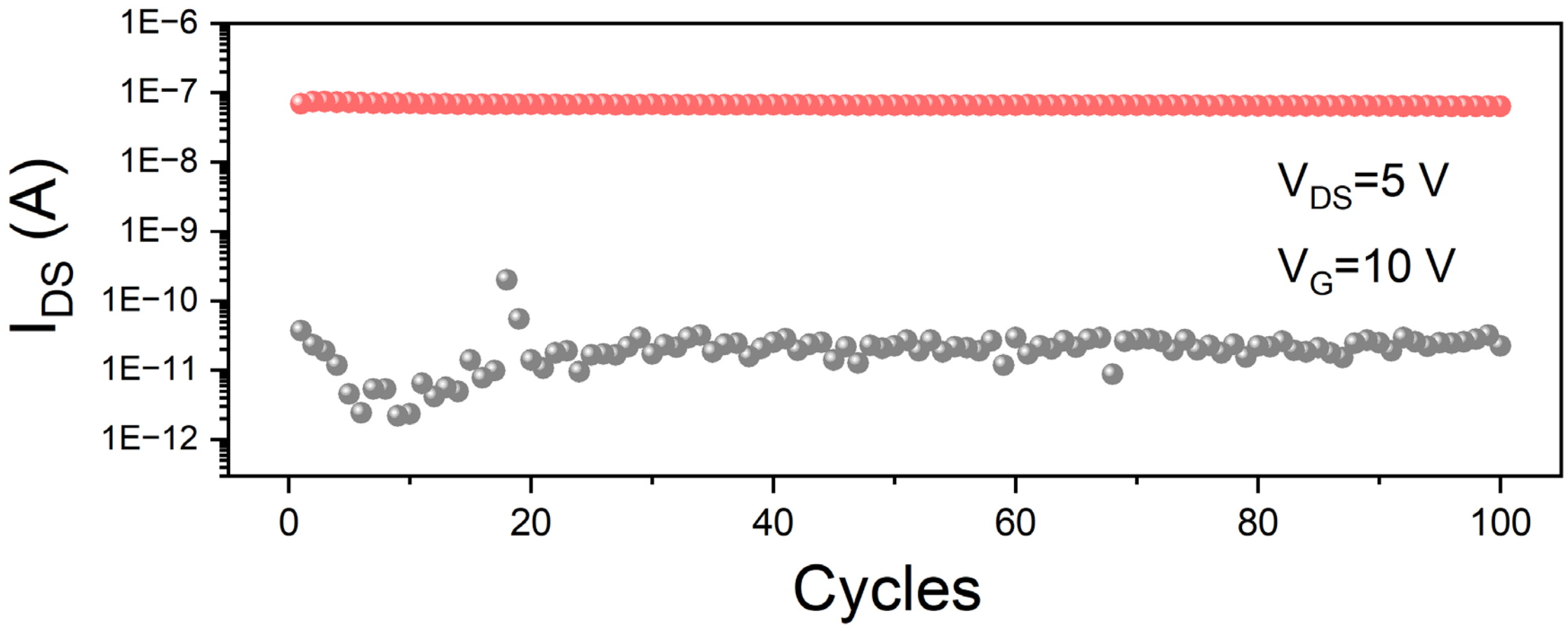

Figure S36. Electrical stability test under 100 cycles.

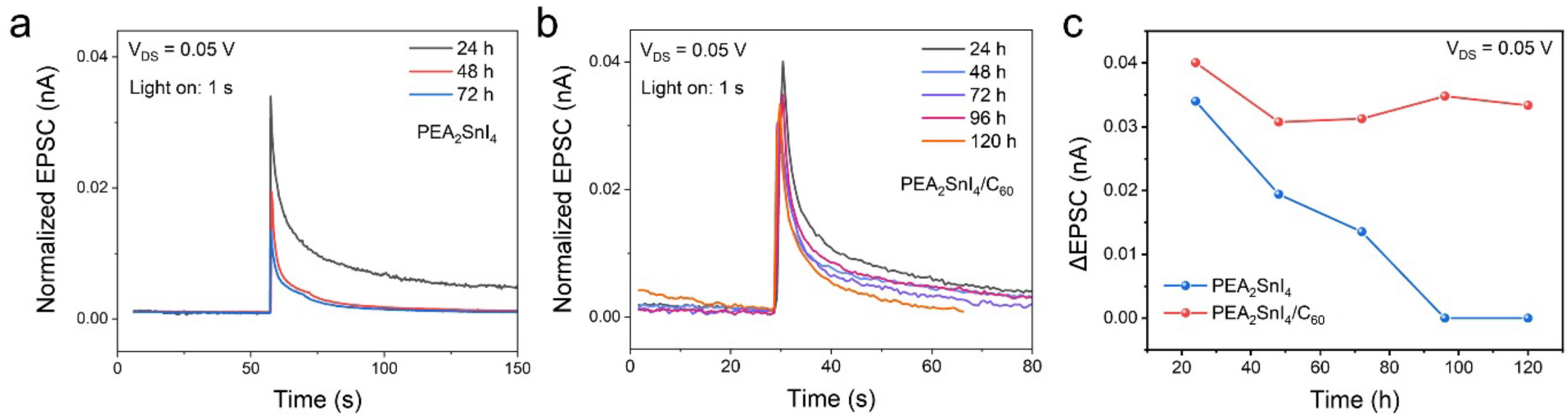

Figure S37. Optical EPSC stability test in 120 h. (a) ESPC stability of PEA_2SnI_4 device. (b) EPSC stability of PEA_2SnI_4/C_{60} device. (c) Statistical summary of stability in 120 h. Light source: 625 nm, 0.08 mW/cm^2. Bias = 0.05 V.

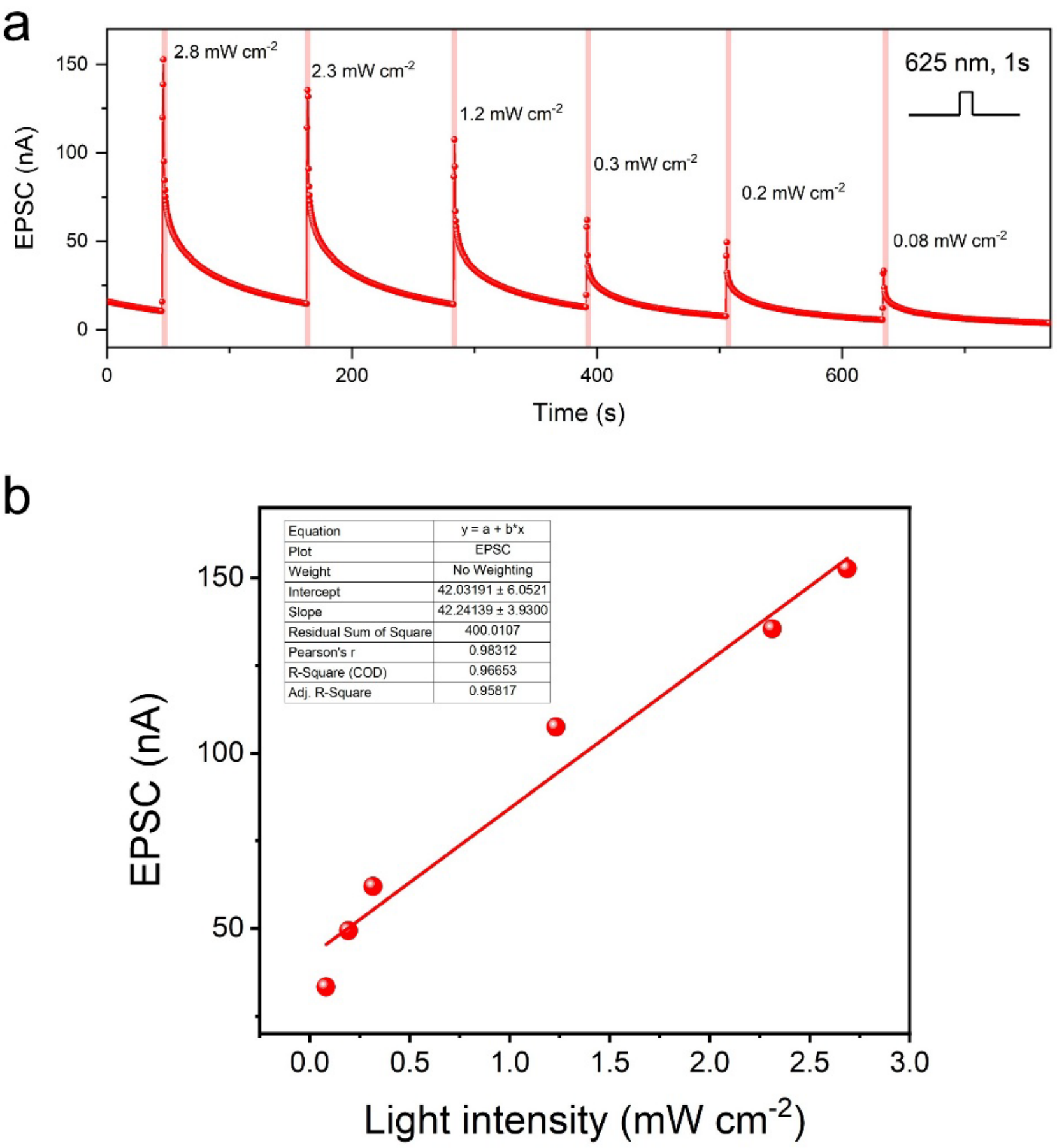

Figure S38. (a) EPSC under single 625 nm light pulse with different intensity ranges from 0.08 mW cm^{-2} to 2.8 mW cm^{-2}. (b) Linear fitting of EPSC peak with different light intensities. Bias = 1 V.

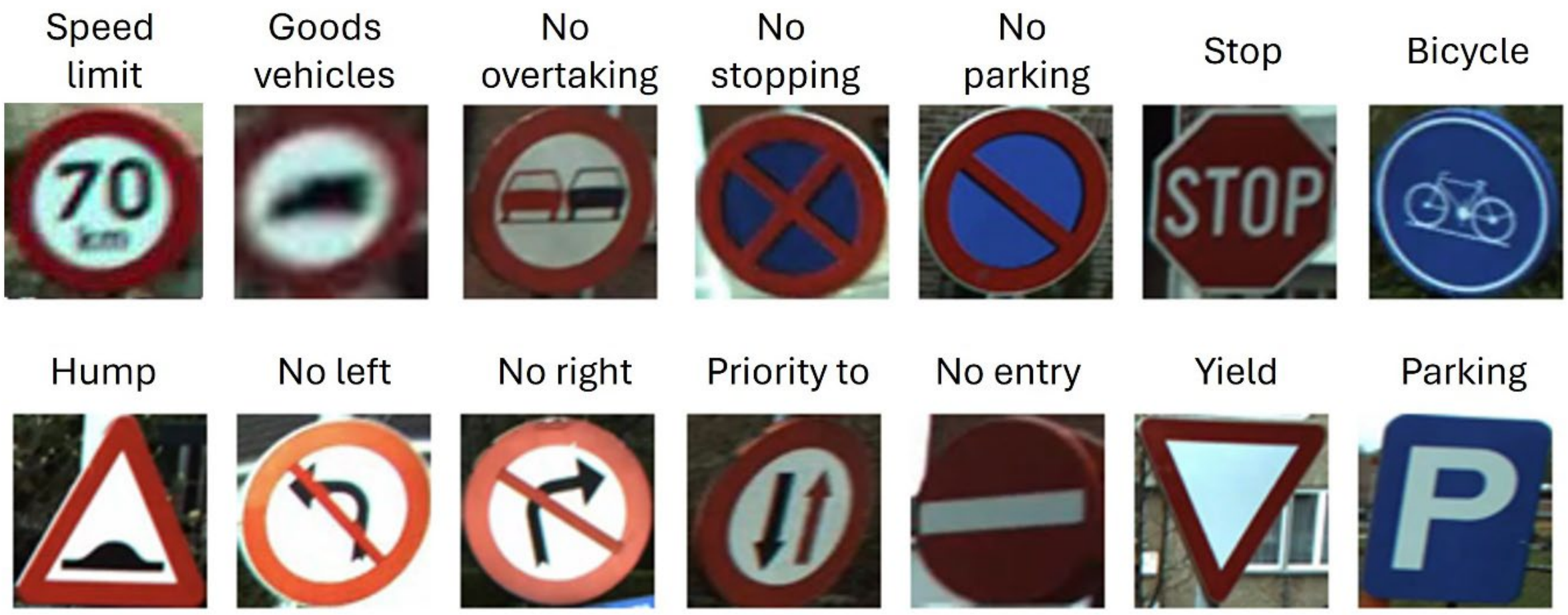

Figure S39. 14 types of traffic signs from the dataset CURE-TSR used for the recognition task. Source images are from the CURE-TSR dataset by Temel, Kwon, Prabhushankar and AlRegib, licensed under Creative Commons Attribution 4.0 International (CC BY 4.0).

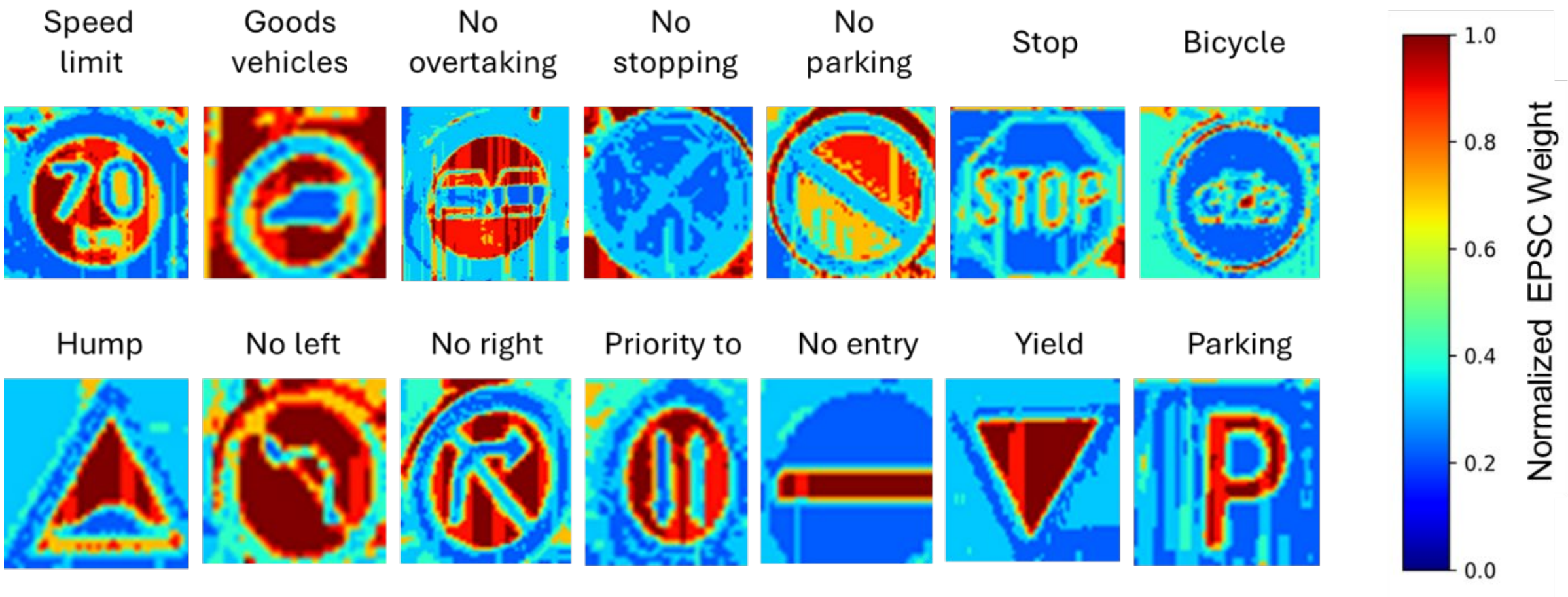

Figure S40. Attention map of different traffic signs with normalized EPSC weight.

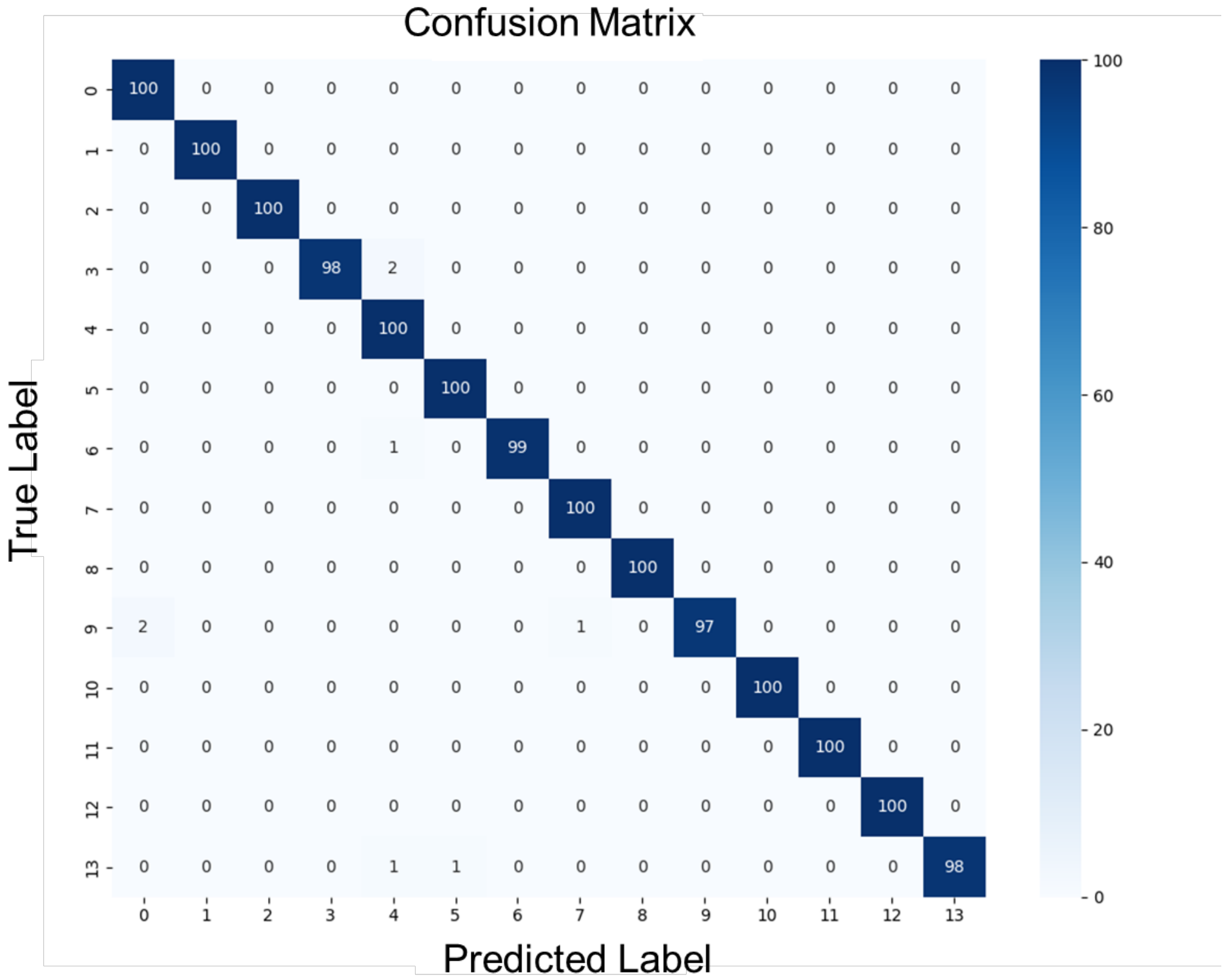

Figure S41. Confusion matrix of CNN model testing on ideal, challenge-free image testing sets.

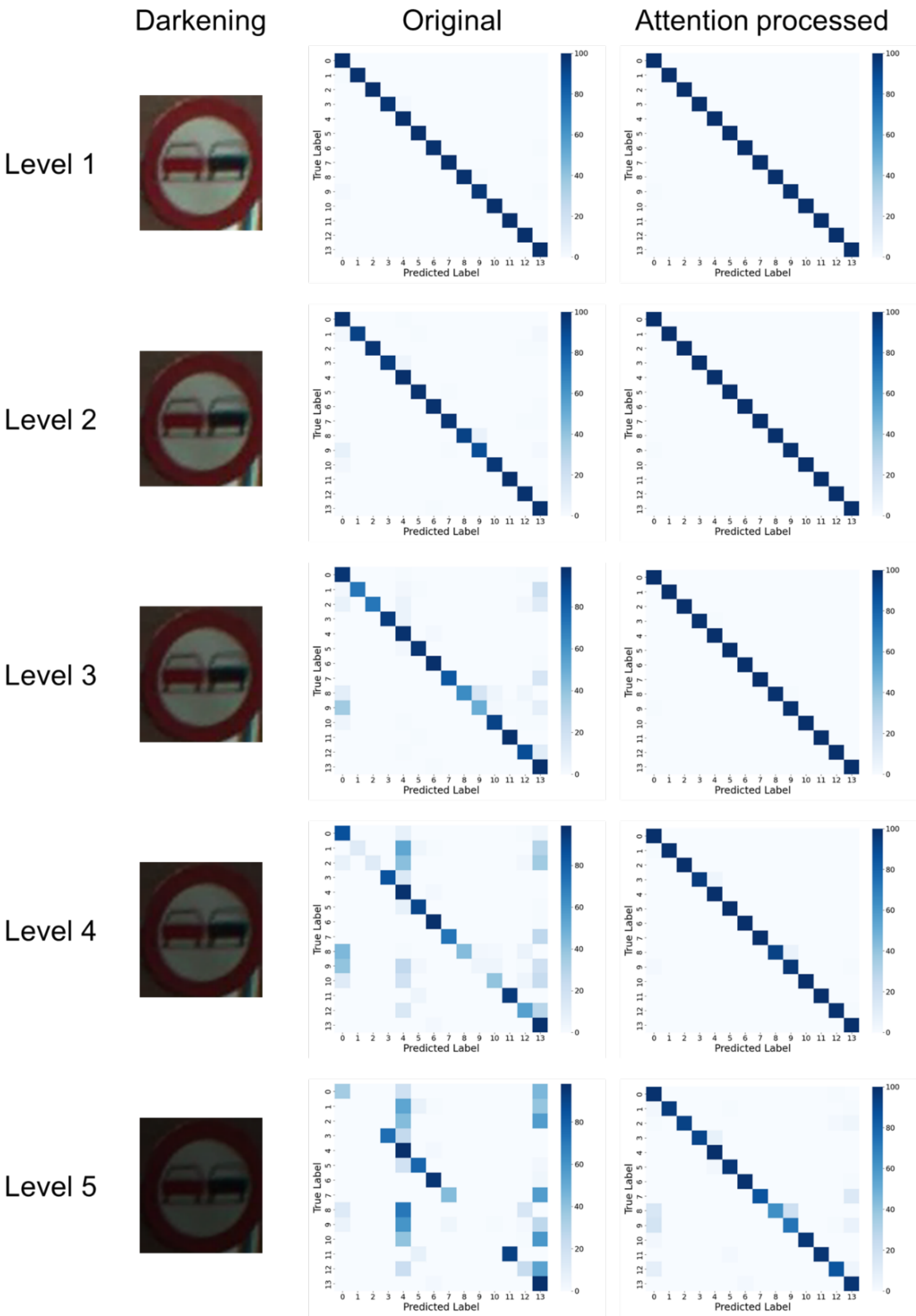

Figure S42. **Dark challenge environment testing.** Confusion matrixes of recognition task for original traffic signs and attention weight enhanced traffic signs under different level of dark challenges.

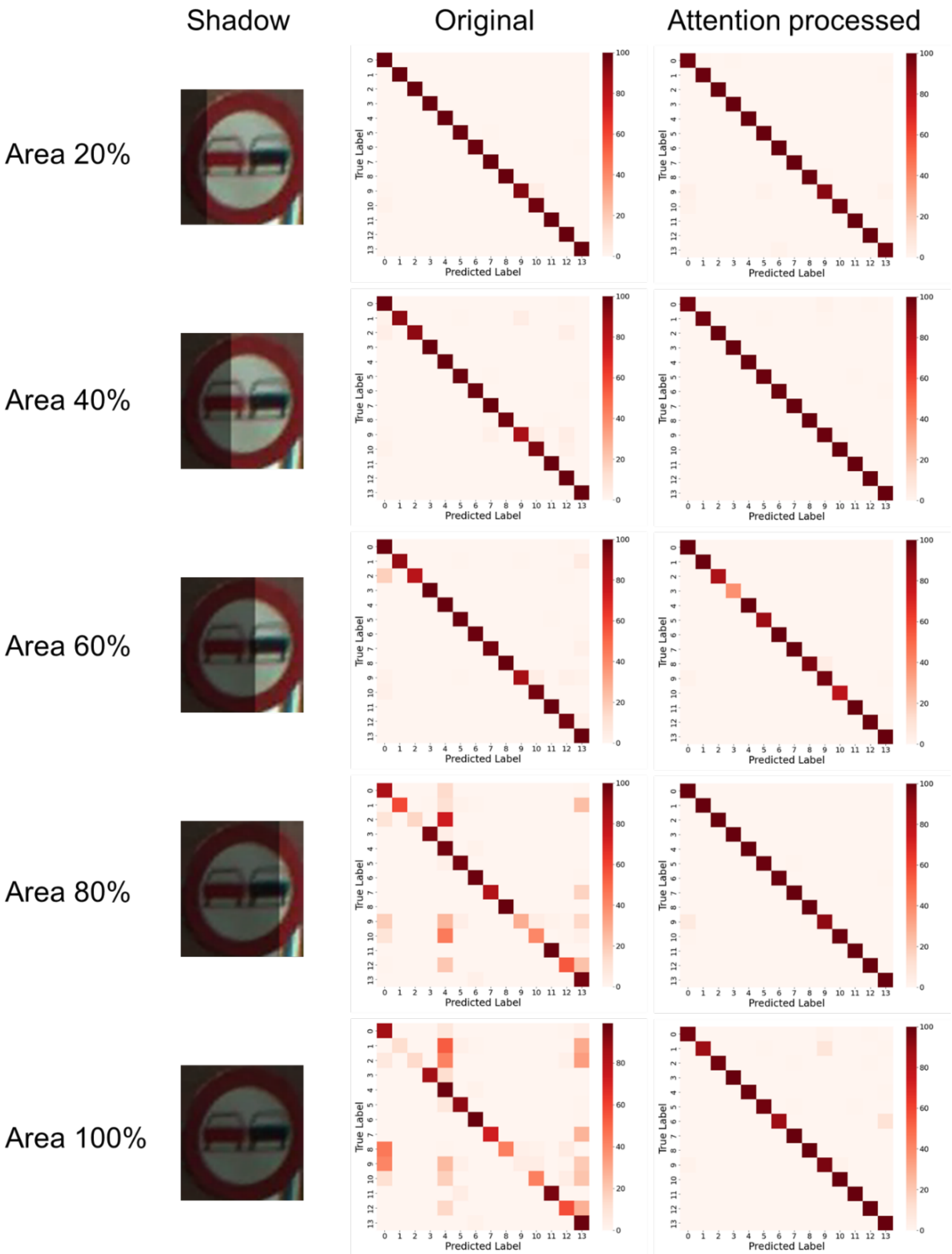

Figure S43. **Shadow challenge environment testing.** Confusion matrixes of recognition task for original traffic signs and attention weight enhanced traffic signs under different level of shadow challenges.

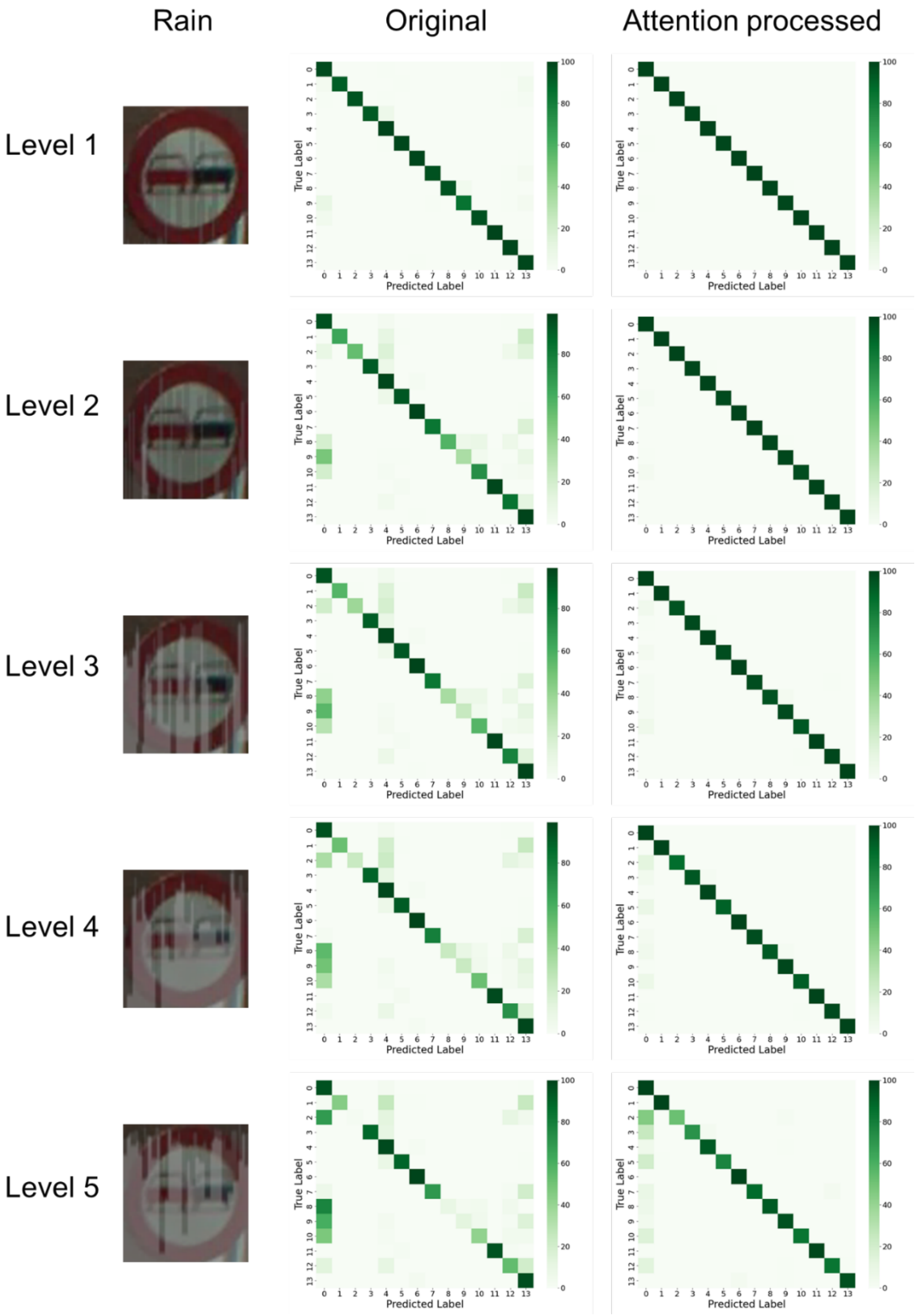

Figure S44. **Shadow challenge environment testing.** Confusion matrixes of recognition task for original traffic signs and attention weight enhanced traffic signs under different level of shadow challenges.

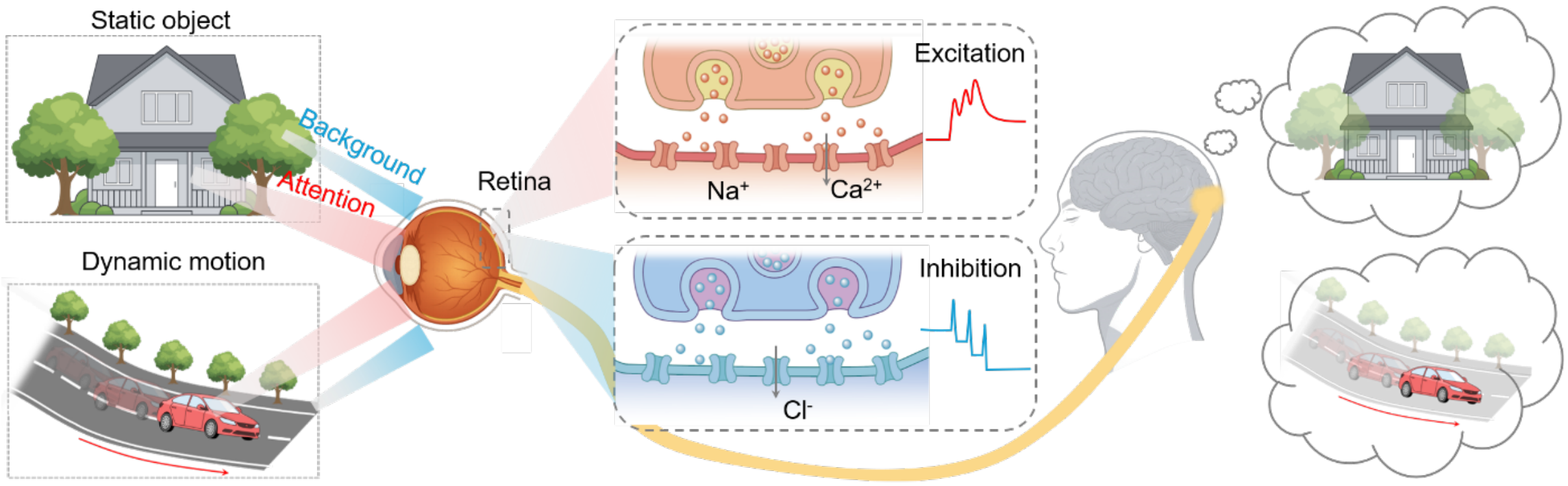

Figure S45. Schematic illustration of visual processing in the retina, where excitation enhances attention to salient features and inhibition suppresses background noise, enabling recognition of both static and dynamic scenes.

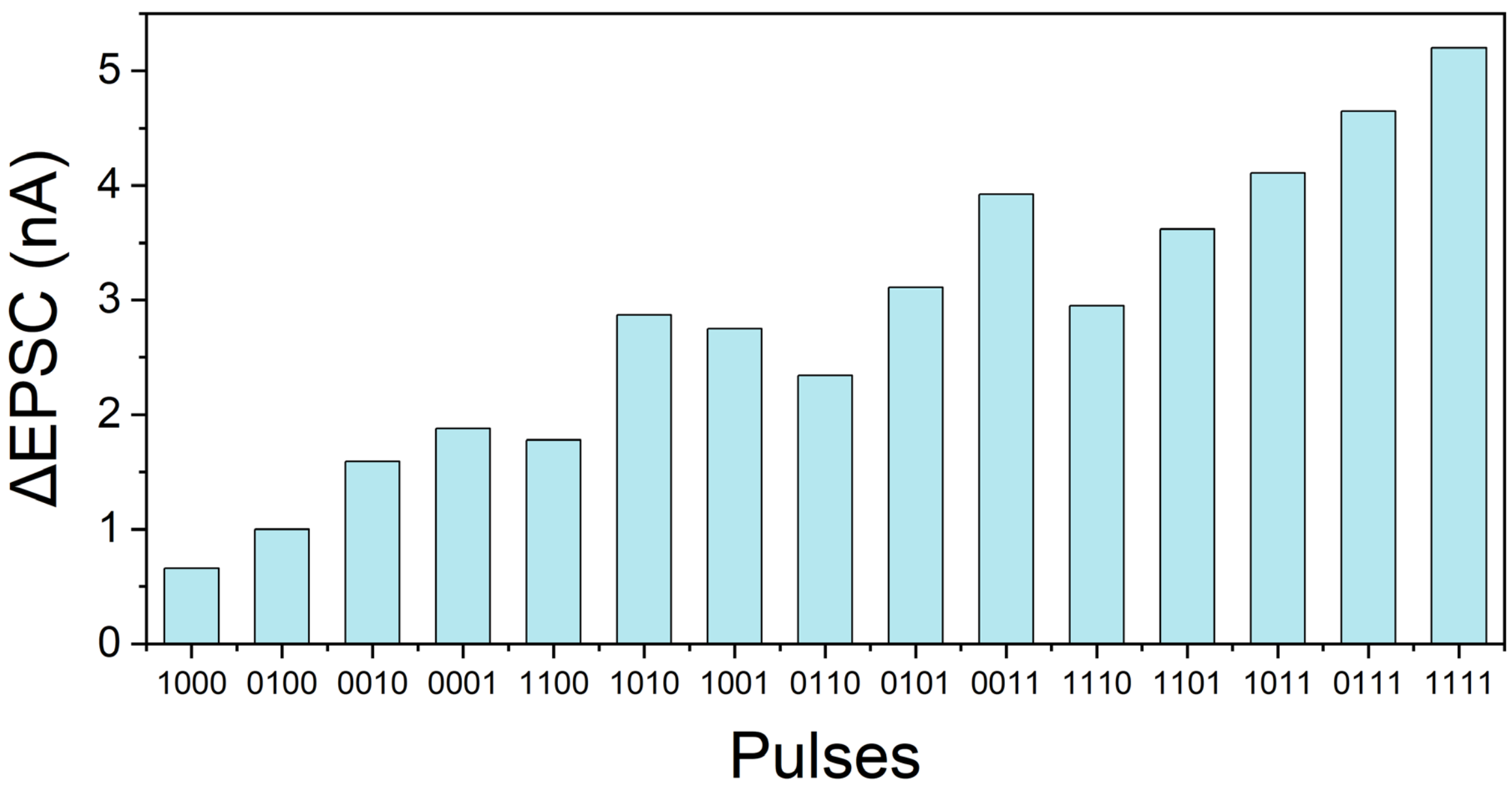

Figure S46. The final EPSC changes of different pulse streams, showing a clear separation.

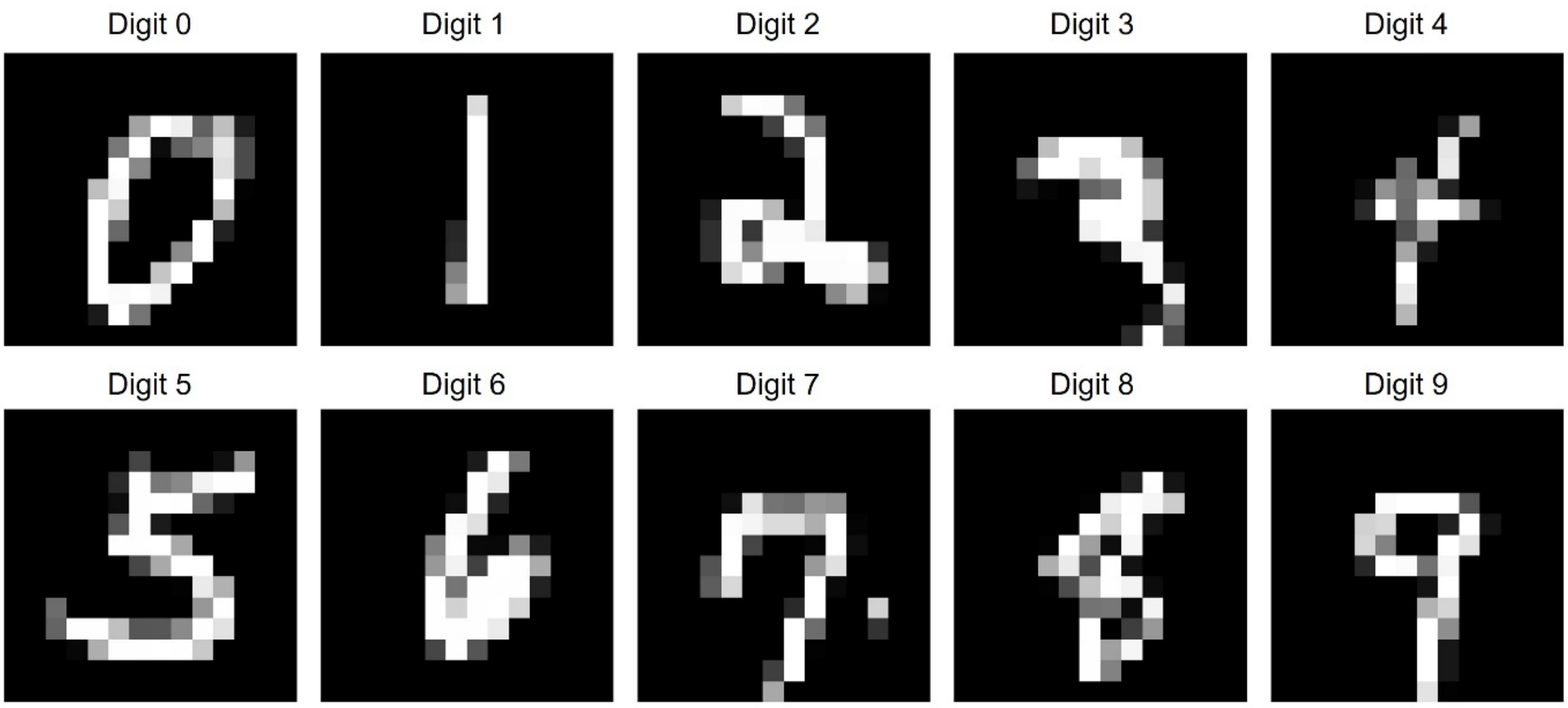

Figure S47. Digit number 0-9 images in 14 × 14 from MNIST dataset.

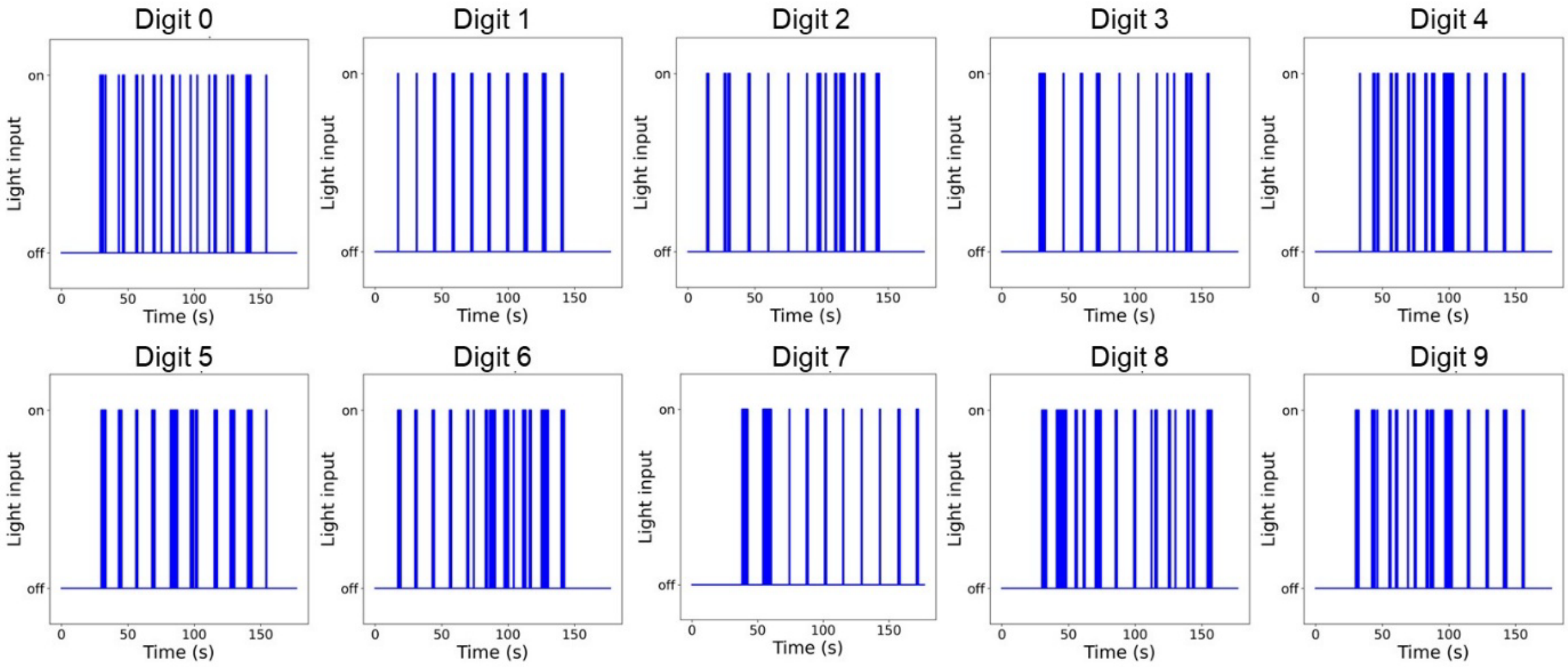

Figure S48. Coded light pulses for digits number 0-9.

REFERENCES

1. P. Slade, C. Atkeson, J. M. Donelan, H. Houdijk, K. A. Ingraham, M. Kim, K. Kong, K. L. Poggensee, R. Riener, M. Steinert, J. Zhang, S. H. Collins, On human-in-the-loop optimization of human-robot interaction. *Nature* **633**, 779–788 (2024).

2. C. A. Aubin, B. Gorissen, E. Milana, P. R. Buskohl, N. Lazarus, G. A. Slipher, C. Keplinger, J. Bongard, F. Iida, J. A. Lewis, R. F. Shepherd, Towards enduring autonomous robots via embodied energy. *Nature* **602**, 393–402 (2022).

3. H. Wang, L. Kong, Z. Fang, R. Zou, Z. Zhang, X. Zhao, Q. Zong, Z. Xie, Symbiotic energy paradigm for self-sustaining aerial robots. *Nat. Rev. Electr. Eng.* **2**, 302–319 (2025).

4. H. Huang, X. Liang, Y. Wang, J. Tang, Y. Li, Y. Du, W. Sun, J. Zhang, P. Yao, X. Mou, F. Xu, J. Zhang, Y. Lu, Z. Liu, J. Wang, Z. Jiang, R. Hu, Z. Wang, Q. Zhang, B. Gao, X. Bai, L. Fang, Q. Dai, H. Yin, H. Qian, H. Wu, Fully integrated multi-mode optoelectronic memristor array for diversified in-sensor computing. *Nat. Nanotechnol.* **20**, 93–103 (2025).

5. N. J. Tye, S. Hofmann, P. Stanley-Marbell, Materials and devices as solutions to computational problems in machine learning. *Nat. Electron.* **6**, 479–490 (2023).

6. F. Zhou, Y. Chai, Near-sensor and in-sensor computing. *Nat. Electron.* **3**, 664–671 (2020).

7. Z. Wang, T. Wan, S. Ma, Y. Chai, Multidimensional vision sensors for information processing. *Nat. Nanotechnol.* **19**, 919–930 (2024).

8. K. Roy, A. Jaiswal, P. Panda, Towards spike-based machine intelligence with neuromorphic computing. *Nature* **575**, 607–617 (2019).

9. L. Mennel, J. Symonowicz, S. Wachter, D. K. Polyushkin, A. J. Molina-Mendoza, T. Mueller, Ultrafast machine vision with 2D material neural network image sensors. *Nature* **579**, 62–66 (2020).

10. J. Chen, Z. Zhou, B. J. Kim, Y. Zhou, Z. Wang, T. Wan, J. Yan, J. Kang, J.-H. Ahn, Y. Chai, Optoelectronic graded neurons for bioinspired in-sensor motion perception. *Nat. Nanotechnol.* **18**, 882–888 (2023).

11. Y. Chai, In-sensor computing for machine vision. *Nature* **579**, 32–33 (2020).

12. Y. Hou, J. Li, J. Yoon, A. M. Knoepfel, D. Yang, L. Zheng, T. Ye, S. Ghosh, S. Priya, K. Wang, Retina-inspired narrowband perovskite sensor array for panchromatic imaging. *Sci. Adv.* **9**, eade2338 (2023).

13. Z. Long, X. Qiu, C. L. J. Chan, Z. Sun, Z. Yuan, S. Poddar, Y. Zhang, Y. Ding, L. Gu, Y. Zhou, W. Tang, A. K. Srivastava, C. Yu, X. Zou, G. Shen, Z. Fan, A neuromorphic bionic eye with filter-free color vision using hemispherical perovskite nanowire array retina. *Nat. Commun.* **14**, 1972 (2023).

14. R. A. John, Y. Demirağ, Y. Shynkarenko, Y. Berezovska, N. Ohannessian, M. Payvand, P. Zeng, M. I. Bodnarchuk, F. Krumeich, G. Kara, I. Shorubalko, M. V. Nair, G. A. Cooke, T. Lippert, G. Indiveri, M. V. Kovalenko, Reconfigurable halide perovskite nanocrystal memristors for neuromorphic computing. *Nat. Commun.* **13**, 2074 (2022).

15. H. Cui, Y. Xiao, Y. Yang, M. Pei, S. Ke, X. Fang, L. Qiao, K. Shi, H. Long, W. Xu, P. Cai, P. Lin, Y. Shi, Q. Wan, C. Wan, A bioinspired in-materia analog photoelectronic reservoir computing for human action processing. *Nat. Commun.* **16**, 2263 (2025).

16. F. Zhou, Z. Zhou, J. Chen, T. H. Choy, J. Wang, N. Zhang, Z. Lin, S. Yu, J. Kang, H. S. P. Wong, Y. Chai, Optoelectronic resistive random access memory for neuromorphic vision sensors. *Nat. Nanotechnol.* **14**, 776–782 (2019).

17. K. Chen, H. Hu, I. Song, H. B. Gobeze, W.-J. Lee, A. Abtahi, K. S. Schanze, J. Mei, Organic optoelectronic synapse based on photon-modulated electrochemical doping. *Nat. Photonics* **17**, 629–637 (2023).

18. B. Pradhan, S. Das, J. Li, F. Chowdhury, J. Cherusseri, D. Pandey, D. Dev, A. Krishnaprasad, E. Barrios, A. Towers, A. Gesquiere, L. Tetard, T. Roy, J. Thomas, Ultrasensitive and ultrathin

phototransistors and photonic synapses using perovskite quantum dots grown from graphene lattice. *Sci. Adv.* **6**, eaay5225 (2020).

19. L. Wang, H. Wang, J. Liu, Y. Wang, H. Shao, W. Li, M. Yi, H. Ling, L. Xie, W. Huang, Negative photoconductivity transistors for visuomorphic computing. *Adv. Mater.* **36**, e2403538 (2024).

20. Z. Wang, L. Lu, J. Meng, T. Wang, Emerging negative photoconductivity effect-based synaptic device for optoelectronic in-sensor computing. *Adv. Mater.* **37**, e2504710 (2025).

21. Y. Wang, Y. Zha, C. Bao, F. Hu, Y. Di, C. Liu, F. Xing, X. Xu, X. Wen, Z. Gan, B. Jia, Monolithic 2D perovskites enabled artificial photonic synapses for neuromorphic vision sensors. *Adv. Mater.* **36**, e2311524 (2024).

22. T. Liu, Z. Yuan, L. Wang, C. Shan, Q. Zhang, H. Chen, H. Wang, W. Wu, L. Huang, Y. Chai, X. Meng, Chelated tin halide perovskite for near-infrared neuromorphic imaging array enabling object recognition and motion perception. *Nat. Commun.* **16**, 4261 (2025).

23. L. Guo, H. Sun, L. Min, M. Wang, F. Cao, L. Li, Two-terminal perovskite optoelectronic synapse for rapid trained neuromorphic computation with high accuracy. *Adv. Mater.* **36**, e2402253 (2024).

24. F. Zhang, M. Shao, C. Wang, W. Wen, W. Shi, M. Qin, H. Huang, X. Wei, Y. Guo, Y. Liu, Photoinduced nonvolatile memory transistor based on lead-free perovskite incorporating fused π-conjugated organic ligands. *Adv. Mater.* **36**, e2307326 (2024).

25. Z. Guo, J. Zhang, B. Yang, L. Li, X. Liu, Y. Xu, Y. Wu, P. Guo, T. Sun, S. Dai, H. Liang, J. Wang, Y. Zou, L. Xiong, J. Huang, Organic high-temperature synaptic phototransistors for energy-efficient neuromorphic computing. *Adv. Mater.* **36**, e2310155 (2024).

26. Y. Lian, J. Han, M. Yang, S. Peng, C. Zhang, C. Han, X. Zhang, X. Liu, H. Zhou, Y. Wang, C. Lan, J. Gou, Y. Jiang, Y. Liao, H. Yu, J. Wang, Tunable bi-directional photoresponse in hybrid $PtSe_{2-x}$ thin films based on precisely controllable selenization engineering. *Adv. Funct. Mater.* **32**, 2205709 (2022).

27. B. Ouyang, J. Wang, G. Zeng, J. Yan, Y. Zhou, X. Jiang, B. Shao, Y. Chai, Bioinspired in-sensor spectral adaptation for perceiving spectrally distinctive features. *Nat. Electron.* **7**, 705–713 (2024).

28. C.-M. Yang, T.-C. Chen, D. Verma, L.-J. Li, B. Liu, W.-H. Chang, C.-S. Lai, Bidirectional all-optical synapses based on a 2D Bi_2O_2Se/graphene hybrid structure for multifunctional optoelectronics. *Adv. Funct. Mater.* **30**, 2001598 (2020).

29. Y. Wang, Y. Zhu, Y. Li, Y. Zhang, D. Yang, X. Pi, Dual-modal optoelectronic synaptic devices with versatile synaptic plasticity. *Adv. Funct. Mater.* **32**, 2107973 (2022).

30. B. Cai, Y. Huang, L. Tang, T. Wang, C. Wang, Q. Sun, D. W. Zhang, L. Chen, All-optically controlled retinomorphic memristor for image processing and stabilization. *Adv. Funct. Mater.* **33**, 2306272 (2023).

31. W. Bai, M. Liang, T. Xuan, T. Gong, L. Bian, H. Li, R.-J. Xie, Ligand engineering enables efficient pure red tin-based perovskite light-emitting diodes. *Angew. Chem. Int. Ed.* **135**, e202312728 (2023).

32. H. Zhou, H. Rao, Y. Jin, W. Zhu, Z. Deng, Y. Zhong, W. Sheng, G. Liu, L. Tan, Y. Chen, Controlled crystallization kinetics via conjugated organic spacers enables ordered epitaxial growth of two-step deposited tin-based perovskite solar cells. *Angew. Chem. Int. Ed.* **64**, e202513416 (2025).

33. S. Zhang, M. Qu, J. Duan, H. Dai, T. Xuan, R. Xie, H. Li, Improving the performance of pure-red 2D tin-based perovskite light-emitting diodes through N-Methylthiourea ligand engineering. *J. Mater. Chem. C* **12**, 14368–14375 (2024).

34. S. You, H. Zhu, Z. Shen, X. Wang, B. Shao, Q. Wang, J. Lu, Y. Yuan, B. D. Dou, E. M. Sanehira, T. Russell, A. Lorenz, Y. Dong, L. Chen, M. Casareto, N. Rolston, M. C. Beard, J. J. Berry, M. Freitag, Y. Yan, O. M. Bakr, K. Zhu, C_{60}-based ionic salt electron shuttle for high-performance inverted perovskite solar modules. *Science* **388**, 964–968 (2025).

35. Y.-T. Yang, Y.-H. Shih, Q.-G. Chen, C.-H. Chen, M.-H. Yu, C.-H. Nieh, B.-H. Lin, W.-C. Chen, W.-Y. Lee, C.-C. Chueh, Revealing the potential of perovskite transistors for dual-modulated synaptic behavior through heterojunction design. *ACS Energy Lett.* **9**, 4564–4571 (2024).

36. F. Yuan, X. Zheng, A. Johnston, Y.-K. Wang, C. Zhou, Y. Dong, B. Chen, H. Chen, J. Z. Fan, G. Sharma, P. Li, Y. Gao, O. Voznyy, H.-T. Kung, Z.-H. Lu, O. M. Bakr, E. H. Sargent, Color-pure red light-emitting diodes based on two-dimensional lead-free perovskites. *Sci. Adv.* **6**, eabb0253 (2020).

37. X. Huang, Q. Li, W. Shi, K. Liu, Y. Zhang, Y. Liu, X. Wei, Z. Zhao, Y. Guo, Y. Liu, Dual-mode learning of ambipolar synaptic phototransistor based on 2D perovskite/organic heterojunction for flexible color recognizable visual system. *Small* **17**, e2102820 (2021).

38. S. B. Anantharaman, J. Lynch, C. E. Stevens, C. Munley, C. Li, J. Hou, H. Zhang, A. Torma, T. Darlington, F. Coen, K. Li, A. Majumdar, P. J. Schuck, A. Mohite, H. Harutyunyan, J. R. Hendrickson, D. Jariwala, Dynamics of self-hybridized exciton–polaritons in 2D halide perovskites. *Light Sci. Appl.* **13**, 1 (2024).

39. E. Choi, Y. Zhang, A. M. Soufiani, M. Lee, R. F. Webster, M. E. Pollard, P. J. Reece, W. Lee, J. Seidel, J. Lim, J.-H. Yun, J. S. Yun, Exploration of sub-bandgap states in 2D halide perovskite single-crystal photodetector. *npj 2D Mater. Appl.* **6**, 43 (2022).

40. J. Zhang, X. Zhu, M. Wang, B. Hu, Establishing charge-transfer excitons in 2D perovskite heterostructures. *Nat. Commun.* **11**, 2618 (2020).

41. Y. Meng, F. Li, C. Lan, X. Bu, X. Kang, R. Wei, S. Yip, D. Li, F. Wang, T. Takahashi, T. Hosomi, K. Nagashima, T. Yanagida, J. C. Ho, Artificial visual systems enabled by quasi–two-dimensional electron gases in oxide superlattice nanowires. *Sci. Adv.* **6**, eabc6389 (2020).

42. J. Pan, Y. Wu, X. Zhang, J. Chen, J. Wang, S. Cheng, X. Wu, X. Zhang, J. Jie, Anisotropic charge trapping in phototransistors unlocks ultrasensitive polarimetry for bionic navigation. *Nat. Commun.* **13**, 6629 (2022).

43. T. Ochiai, T. Akazawa, Y. Miyatake, K. Sumita, S. Ohno, S. Monfray, F. Boeuf, K. Toprasertpong, S. Takagi, M. Takenaka, Ultrahigh-responsivity waveguide-coupled optical power monitor for Si photonic circuits operating at near-infrared wavelengths. *Nat. Commun.* **13**, 7443 (2022).

44. F. Zhou, I. Abdelwahab, K. Leng, K. P. Loh, W. Ji, 2D perovskites with giant excitonic optical nonlinearities for high-performance sub-bandgap photodetection. *Adv. Mater.* **31**, e1904155 (2019).

45. D. Meggiolaro, S. G. Motti, E. Mosconi, A. J. Barker, J. Ball, C. A. R. Perini, F. Deschler, A. Petrozza, F. De Angelis, Iodine chemistry determines the defect tolerance of lead-halide perovskites. *Energ. Environ. Sci.* **11**, 702–713 (2018).

46. D. J. Keeble, J. Wiktor, S. K. Pathak, L. J. Phillips, M. Dickmann, K. Durose, H. J. Snaith, W. Egger, Identification of lead vacancy defects in lead halide perovskites. *Nat. Commun.* **12**, 5566 (2021).

47. X. Zhang, M. E. Turiansky, J.-X. Shen, C. G. Van de Walle, Iodine interstitials as a cause of nonradiative recombination in hybrid perovskites. *Phys. Rev. B* **101**, 140101 (2020).

48. G. J. W. Aalbers, T. P. A. van der Pol, K. Datta, W. H. M. Remmerswaal, M. M. Wienk, R. A. J. Janssen, Effect of sub-bandgap defects on radiative and non-radiative open-circuit voltage losses in perovskite solar cells. *Nat. Commun.* **15**, 1276 (2024).

49. T. Lu, Z. Dang, Y. Luo, Y. Li, X. Rao, Z. Zhang, Y. Hu, Z. Chen, Z. Li, U. Petralanda, J. Xie, J. Yu, P. Gao, Lattice expansion of hybrid perovskite inhibits halogen interstitial generation and enhances solar cell performance. *Nat. Commun.* **16**, 8591 (2025).

50. O. J. Sandberg, C. Kaiser, S. Zeiske, N. Zarrabi, S. Gielen, W. Maes, K. Vandewal, P. Meredith, A. Armin, Mid-gap trap state-mediated dark current in organic photodiodes. *Nat. Photonics* **17**, 368–374 (2023).

51. N. Zarrabi, O. J. Sandberg, S. Zeiske, W. Li, D. B. Riley, P. Meredith, A. Armin, Charge-generating mid-gap trap states define the thermodynamic limit of organic photovoltaic devices. *Nat. Commun.* **11**, 5567 (2020).

52. R. S. Zucker, W. G. Regehr, Short-term synaptic plasticity. *Annu. Rev. Physiol.* **64**, 355–405 (2002).

53. J. Ko, C. Ock, H. Gim, K. Hong, Y. Lee, K. C. Kwon, Two-dimensional materials for artificial sensory devices: Advancing neuromorphic sensing technology. *npj 2D Mater. Appl.* **9**, 35 (2025).

54. R. Chen, H. Yang, R. Li, G. Yu, Y. Zhang, J. Dong, D. Han, Z. Zhou, P. Huang, L. Liu, X. Liu, J. Kang, Thin-film transistor for temporal self-adaptive reservoir computing with closed-loop architecture. *Sci. Adv.* **10**, eadl1299 (2024).

55. S. Stepney, Physical reservoir computing: A tutorial. *Nat. Comput.* **23**, 665–685 (2024).

56. Y. Zhong, J. Tang, X. Li, B. Gao, H. Qian, H. Wu, Dynamic memristor-based reservoir computing for high-efficiency temporal signal processing. *Nat. Commun.* **12**, 408 (2021).

57. H. Zhang, S. Wang, L. Wang, S. Li, H. Liu, X. Zhu, Y. Chen, G. Xu, M. Zhang, Q. Liu, R. Wang, K. Xiao, Bio-inspired retina by regulating ion-confined transport in hydrogels. *Adv. Mater.* **37**, e2500809 (2025).

58. J. Sun, S. Oh, Y. Choi, S. Seo, M. J. Oh, M. Lee, W. B. Lee, P. J. Yoo, J. H. Cho, J.-H. Park, Optoelectronic synapse based on IGZO-alkylated graphene oxide hybrid structure. *Adv. Funct. Mater.* **28**, 1804397 (2018).

59. Y. Wang, Z. Lv, J. Chen, Z. Wang, Y. Zhou, L. Zhou, X. Chen, S.-T. Han, Photonic synapses based on inorganic perovskite quantum dots for neuromorphic computing. *Adv. Mater.* **30**, 1802883 (2018).

60. J. J. Yu, L. Y. Liang, L. X. Hu, H. X. Duan, W. H. Wu, H. L. Zhang, J. H. Gao, F. Zhuge, T. C. Chang, H. T. Cao, Optoelectronic neuromorphic thin-film transistors capable of selective attention and with ultra-low power dissipation. *Nano Energy* **62**, 772–780 (2019).

61. N. Duan, Y. Li, H.-C. Chiang, J. Chen, W.-Q. Pan, Y.-X. Zhou, Y.-C. Chien, Y.-H. He, K.-H. Xue, G. Liu, T.-C. Chang, X.-S. Miao, An electro-photo-sensitive synaptic transistor for edge neuromorphic visual systems. *Nanoscale* **11**, 17590–17599 (2019).

62. K. Wang, S. Dai, Y. Zhao, Y. Wang, C. Liu, J. Huang, Light-stimulated synaptic transistors fabricated by a facile solution process based on inorganic perovskite quantum dots and organic semiconductors. *Small* **15**, e1900010 (2019).

63. L. Yin, C. Han, Q. Zhang, Z. Ni, S. Zhao, K. Wang, D. Li, M. Xu, H. Wu, X. Pi, D. Yang, Synaptic silicon-nanocrystal phototransistors for neuromorphic computing. *Nano Energy* **63**, 103859 (2019).

64. H.-L. Park, H. Kim, D. Lim, H. Zhou, Y.-H. Kim, Y. Lee, S. Park, T.-W. Lee, Retina-inspired carbon nitride-based photonic synapses for selective detection of UV light. *Adv. Mater.* **32**, e1906899 (2020).

65. L. Yin, W. Huang, R. Xiao, W. Peng, Y. Zhu, Y. Zhang, X. Pi, D. Yang, Optically stimulated synaptic devices based on the hybrid structure of silicon nanomembrane and perovskite. *Nano Lett.* **20**, 3378–3387 (2020).

66. Z. Liu, S. Dai, Y. Wang, B. Yang, D. Hao, D. Liu, Y. Zhao, L. Fang, Q. Ou, S. Jin, J. Zhao, J. Huang, Photoresponsive transistors based on lead-free perovskite and carbon nanotubes. *Adv. Funct. Mater.* **30**, 1906335 (2020).

67. T. Chen, X. Wang, D. Hao, S. Dai, Q. Ou, J. Zhang, J. Huang, Photonic synapses with ultra-low energy consumption based on vertical organic field-effect transistors. *Adv. Opt. Mater.* **9**, 2002030 (2021).

68. J. Shi, J. Jie, W. Deng, G. Luo, X. Fang, Y. Xiao, Y. Zhang, X. Zhang, X. Zhang, A fully solution-printed photosynaptic transistor array with ultralow energy consumption for artificial-vision neural networks. *Adv. Mater.* **34**, e2200380 (2022).

69. Y. Cao, X. Sha, X. Bai, Y. Shao, Y. Gao, Y.-M. Wei, L. Meng, N. Zhou, J. Liu, B. Li, X.-F. Yu, J. Li, Ultralow light-power consuming photonic synapses based on ultrasensitive

perovskite/indium-gallium-zinc-oxide heterojunction phototransistors. *Adv. Electron. Mater.* **8**, 2100902 (2022).

70. S. Wang, H. Chen, T. Liu, Y. Wei, G. Yao, Q. Lin, X. Han, C. Zhang, H. Huang, Retina-inspired organic photonic synapses for selective detection of SWIR light. *Angew. Chem. Int. Ed.* **62**, e202213733 (2023).

71. L. Xu, J. Liu, X. Guo, S. Liu, X. Lai, J. Wang, M. Yu, Z. Xie, H. Peng, X. Zou, X. Wang, R. Huang, M. He, Ultrasensitive dim-light neuromorphic vision sensing via momentum-conserved reconfigurable van der Waals heterostructure. *Nat. Commun.* **15**, 9011 (2024).

72. R. Yang, Y. Wang, S. Li, D. Hu, Q. Chen, F. Zhuge, Z. Ye, X. Pi, J. Lu, All-optically controlled artificial synapse based on full oxides for low-power visible neural network computing. *Adv. Funct. Mater.* **34**, 2312444 (2024).

73. Z. Zhao, Z. Hu, M. Deng, E. Hong, P. Wang, Z. Li, X. Fang, Bias-switchable photodetection and photosynapse dual-functional devices based on 2D perovskite/organic heterojunction for imaging-to-recognition conversion. *Adv. Mater.* **37**, e2416033 (2025).

74. T. Liu, H. Wang, C. Sun, Z. Yuan, X. Wang, L. Wang, J. Wang, S. Wang, Q. Zhang, L. Huang, W. Wu, L. Li, X. Meng, Suppression of tin oxidation via Sn→B bonding interactions for high-resolution lead-free perovskite neuromorphic imaging sensors. *Adv. Mater.* **37**, e2502015 (2025).

75. Z. Cheng, T. Wang, J. Zhu, Y. He, S. Liu, M.-Y. Li, H. Lu, X. Wen, J. Lee, S. Liu, S. Mao, All-inorganic lead-free $Cs_2AgBiBr_6$/ZnO artificial retina synapse based on photoelectric synergistic dual-mechanism for neuromorphic computing. *Small* **21**, e2411129 (2025).

76. Y. Chen, J. Xia, Y. Qu, H. Zhang, T. Mei, X. Zhu, G. Xu, D. Li, L. Wang, Q. Liu, K. Xiao, Ephaptic coupling in ultralow-power ion-gel nanofiber artificial synapses for enhanced working memory. *Adv. Mater.* **37**, e2419013 (2025).

77. D. Temel, G. Kwon, M. Prabhushankar, G. AlRegib, CURE-TSR: Challenging unreal and real environments for traffic sign recognition, Zenodo (2020); https://doi.org/10.5281/zenodo.3903066.

78. D. Temel, G. Kwon, M. Prabhushankar, G. AlRegib, CURE-TSR: Challenging unreal and real environments for traffic sign recognition. arXiv:1712.02463 [cs.CV] (2017).

79. G. Kresse, J. Hafner, Ab initio molecular dynamics for liquid metals. *Phys. Rev. B* **47**, 558–561 (1993).

80. G. Kresse, J. Hafner, Ab initio molecular-dynamics simulation of the liquid-metal–amorphous-semiconductor transition in germanium. *Phys. Rev. B* **49**, 14251–14269 (1994).

81. G. Kresse, J. Furthmüller, Efficiency of ab-initio total energy calculations for metals and semiconductors using a plane-wave basis set. *Comput. Mater. Sci.* **6**, 15–50 (1996).

82. G. Kresse, J. Furthmüller, Efficient iterative schemes for ab initio total-energy calculations using a plane-wave basis set. *Phys. Rev. B* **54**, 11169–11186 (1996).

83. J. P. Perdew, M. Ernzerhof, K. Burke, Rationale for mixing exact exchange with density functional approximations. *J. Chem. Phys.* **105**, 9982–9985 (1996).

84. Y. Hinuma, G. Pizzi, Y. Kumagai, F. Oba, I. Tanaka, Band structure diagram paths based on crystallography. *Comput. Mater. Sci.* **128**, 140–184 (2017).

85. A. Togo, K. Shinohara, I. Tanaka, Spglib: A software library for crystal symmetry search. *Sci. Technol. Adv. Mater. Methods* **4**, 2384822 (2024).

86. V. Wang, N. Xu, J.-C. Liu, G. Tang, W.-T. Geng, VASPKIT: A user-friendly interface facilitating high-throughput computing and analysis using VASP code. *Comput. Phys. Commun.* **267**, 108033 (2021).

87. S. Fu, X. Huang, G. Gao, P. St. Petkov, W. Gao, J. Zhang, L. Gao, H. Zhang, M. Liu, M. Hambsch, W. Zhang, J. Zhang, K. Li, U. Kaiser, S. S. P. Parkin, S. C. B. Mannsfeld, T. Zhu, H. I. Wang, Z. Wang, R. Dong, X. Feng, M. Bonn, Unveiling high-mobility hot carriers in a two-dimensional conjugated coordination polymer. *Nat. Mater.* **24**, 1457–1464 (2025).

88. J. Song, Y. Zhou, N. P. Padture, B. D. Huey, Anomalous 3D nanoscale photoconduction in hybrid perovskite semiconductors revealed by tomographic atomic force microscopy. *Nat. Commun.* **11**, 3308 (2020).

89. L. Bernstein, A. Sludds, C. Panuski, S. Trajtenberg-Mills, R. Hamerly, D. Englund, Single-shot optical neural network. *Sci. Adv.* **9**, eadg7904 (2023).

90. V. Tripathi, B. Murmann, An 8-bit 450-MS/s single-bit/cycle SAR ADC in 65-nm CMOS, in 2013 Proceedings of the ESSCIRC (ESSCIRC), Bucharest, Romania, 16–20 September 2013 (IEEE, 2013), pp. 117–120.

91. J. Song, J. Meng, C. Lu, T. Wang, C. Wan, H. Zhu, Q. Sun, D. W. Zhang, L. Chen, Self-powered optoelectronic synaptic device for both static and dynamic reservoir computing. *Nano Energy* **134**, 110574 (2025).

92. Y.-C. Yao, C.-J. Lee, Y.-J. Chen, J.-Z. Feng, H. Oh, C.-S. Lue, J.-K. Sheu, Y.-J. Lee, All-inorganic perovskite quantum-dot optical neuromorphic synapses for near-sensor colored image recognition. *Adv. Sci.* **12**, e2409933 (2025).

93. W. Liu, J. Wang, J. Guo, L. Wang, Z. Gu, H. Wang, H. Fang, Efficient carbon-based optoelectronic synapses for dynamic visual recognition. *Adv. Sci.* **12**, e2414319 (2025).

94. J. Chen, Y. Huang, H. Wen, Y. Wang, H. Li, X. Zheng, X. Wang, Z. Ma, T. Wang, S. Yan, K. Wang, L. Zhao, High sensitivity optoelectronic artificial synapse based on GaN porous nanocone array for neuromorphic computing. *Adv. Opt. Mater.* **13**, 01078 (2025).

95. Y. Yuan, Z. Xu, D. Zhang, C. Huang, W. Xu, Air-stable hydrogen-substituted graphdiyne/2D halide perovskite heterojunction for self-powered neuromorphic vision. *Adv. Funct. Mater.* **35**, 2504542 (2025).

96. W. Qiu, Y. Huang, L.-A. Kong, Y. Chen, W. Liu, Z. Wang, J. Sun, Q. Wan, J. H. Cho, J. Yang, Y. Gao, Optoelectronic In-Ga-Zn-O memtransistors for artificial vision system. *Adv. Funct. Mater.* **30**, 2002325 (2020).

97. C. Qian, S. Oh, Y. Choi, J.-H. Kim, J. Sun, H. Huang, J. Yang, Y. Gao, J.-H. Park, J. H. Cho, Solar-stimulated optoelectronic synapse based on organic heterojunction with linearly potentiated synaptic weight for neuromorphic computing. *Nano Energy* **66**, 104095 (2019).

98. J. Zhang, P. Guo, Z. Guo, L. Li, T. Sun, D. Liu, L. Tian, G. Zu, L. Xiong, J. Zhang, J. Huang, Retina-inspired artificial synapses with ultraviolet to near-infrared broadband responses for energy-efficient neuromorphic visual systems. *Adv. Funct. Mater.* **33**, 2302885 (2023).

99. J. Zhang, Z. Guo, T. Sun, P. Guo, X. Liu, H. Gao, S. Dai, L. Xiong, J. Huang, Energy-efficient organic photoelectric synaptic transistors with environment-friendly $CuInSe_2$ quantum dots for broadband neuromorphic computing. *SmartMat* **5**, e1246 (2024).

100. S. Das, V. Pal, S. Mukherjee, S. Das, C. S. Tiwary, S. K. Ray, Multi-wavelength optoelectronic synaptic transistors based on transition metal telluride-sulfide heterostructures. *Adv. Opt. Mater.* **12**, 2400037 (2024).

101. J.-L. Chen, T.-C. Chiang, P.-T. Liu, All-metal-oxide heterojunction optoelectronic synapses with multilevel memory for artificial visual perception applications. *Small* **21**, e2502271 (2025).

102. K. Chen, Q. Dai, Y. Tian, W. Chen, C. Ai, Y. Cao, X. Yuan, Q. Wang, Y. Shi, Y. Li, Ultra-low-power vertical organic synaptic phototransistors for neuromorphic vision preprocessing. *Adv. Funct. Mater.* **35**, 2508673 (2025).

103. Y. Rong, D. Yu, X. Zhang, T. Wang, J. Wang, Y. Li, T. Zhao, R. He, Y. Gao, C. Huang, S. Xiao, J. Qin, S. Bai, H. Zhu, A. Liu, Y. Chen, Q. Song, Perovskite thin-film transistors for ultra-low-voltage neuromorphic visions. *Adv. Sci.* **11**, e2410015 (2024).

104. J. Han, H. Mao, Y. Lin, K. Peng, L. Meng, Y. Ding, X. Shan, Y. Tao, J. Shi, Z. Wang, H. Zhu, X. Zhao, H. Xu, Y. Liu, Near-infrared optoelectronic memristor with all-optical modulation for microscopic biological motion recognition. *Adv. Mater.* **37**, e10185 (2025).

105. J. Han, M. He, M. Yang, Q. Han, F. Wang, F. Zhong, M. Xu, Q. Li, H. Zhu, C. Shan, W. Hu, X. Chen, X. Wang, J. Gou, Z. Wu, J. Wang, Light-modulated vertical heterojunction phototransistors with distinct logical photocurrents. *Light Sci. Appl.* **9**, 167 (2020).

106. Y.-X. Hou, Y. Li, Z.-C. Zhang, J.-Q. Li, D.-H. Qi, X.-D. Chen, J.-J. Wang, B.-W. Yao, M.-X. Yu, T.-B. Lu, J. Zhang, Large-scale and flexible optical synapses for neuromorphic computing and integrated visible information sensing memory processing. *ACS Nano* **15**, 1497–1508 (2021).

107. T. Ahmed, M. Tahir, M. X. Low, Y. Ren, S. A. Tawfik, E. L. H. Mayes, S. Kuriakose, S. Nawaz, M. J. S. Spencer, H. Chen, M. Bhaskaran, S. Sriram, S. Walia, Fully light-controlled memory and neuromorphic computation in layered black phosphorus. *Adv. Mater.* **33**, e2004207 (2021).

108. Y.-C. Mi, C.-H. Yang, L.-C. Shih, J.-S. Chen, All-optical-controlled excitatory and inhibitory synaptic signaling through bipolar photoresponse of an oxide-based phototransistor. *Adv. Opt. Mater.* **11**, 2300089 (2023).

109. S. Ge, F. Huang, J. He, Z. Xu, Z. Sun, X. Han, C. Wang, L.-B. Huang, C. Pan, Bidirectional photoresponse in perovskite-ZnO heterostructure for fully optical-controlled artificial synapse. *Adv. Opt. Mater.* **10**, 2200409 (2022).

110. L. Hu, J. Yang, J. Wang, P. Cheng, L. O. Chua, F. Zhuge, All-optically controlled memristor for optoelectronic neuromorphic computing. *Adv. Funct. Mater.* **31**, 2005582 (2021).
